# Surveillance of adverse drug reactions at an adverse drug reaction monitoring centre in Central India: a 7-year surveillance study

**DOI:** 10.1136/bmjopen-2021-052737

**Published:** 2021-10-03

**Authors:** Megha Sharma, Ruchi Baghel, Sunil Thakur, Sandeep Adwal

**Affiliations:** 1 Department of Global Public Health, Karolinska Institutet, Stockholm, Sweden; 2 Department of Pharmacology, RD Gardi Medical College, Ujjain, India; 3 Department of Pharmacology, Zydus Medical College and Hospital, Dahod, Gujarat, India

**Keywords:** clinical pharmacology, health & safety, risk management, adverse events, toxicity, pharmacology

## Abstract

**Objectives:**

To analyse and present the occurrence and severity of spontaneous adverse drug reaction (ADR) reports prospectively registered at an ADR monitoring centre (AMC) in Central India.

**Setting and data:**

The survey was conducted between 2013 and 2019 at an ADR Monitoring Centre in Central India. ADRs were recorded using the standard ‘Suspected ADR Reporting form’.

**Outcome measures:**

The causality of the ADRs were categorised using the WHO causality assessment scale to assess the relationship between a drug and the occurrence of an ADR.

**Results:**

Totally 1980 spontaneous ADRs were reported involving 960 patients and 1316 drugs prescriptions. The occurrence of ADRs was common among male patients (64%) and patients of age between 19 and 65 years (81%). Antimicrobials caused 29% ADRs, followed by drugs of antiretroviral therapy (19%). Zidovudine caused most ADRs (88%) followed by ethambutol and ciprofloxacin. The ADRs of skin and subcutaneous tissue disorders (28%) were most common among all system organ classes followed by gastrointestinal systems (18%). Four per cent of all reported ADRs were severe. A peak of ADR reports was attained in 2016 with 224 reports, which decreased to 127 in 2019.

**Conclusion:**

A high number of ADRs caused by antimicrobials is an alarming situation, which adds up to antimicrobial resistance. Judicious use of antimicrobials is yet again proven as need of the hour. Under-reporting of ADRs is evident in our study and is a major factor for the delay in the withdrawal of drugs responsible for causing ADRs. Interventions in terms of training and feedback are suggested to encourage and improve ADR reporting.

**Strengths and limitations of this study**

- This is first long-term study that presents the adverse drug reactions (ADRs) reported from Central India.
- The spontaneous self-reporting and voluntary reporting study design used is easy and low resource demanding.
- The data were collected based on spontaneous reporting rather than active surveillance, which would have been a better method to overcome the issue of under-reporting.
- There was no option to follow the patient or patient’s record to confirm the final outcome, if not mentioned.
- The space provided in ADR form is insufficient to record details of antibiotic susceptibility test reports, crucial to generate signals related to antibiotic resistance.

## Background

Pharmacovigilance is a process of postmarketing surveillance of drugs that continues throughout the drug lifecycle. It is essential in analysing and managing the risks associated with drugs that are available for the use of the general population.^1^ The results of pharmacovigilance certify the effectiveness and safety of drugs in terms of adverse drug reactions (ADRs). The occurrence of ADRs can prolong hospitalisation and thereby increase the cost of hospital stay and treatment and initiate the requirement of additional clinical investigations in severe cases. WHO defines ‘Pharmacovigilance’ as the science and activities relating to the detection, assessment, understanding and prevention of adverse effects or any other possible drug-related problems, including herbal medicines.^2^ The WHO Collaborating Centre (WHOCC) for International Drug Monitoring at Uppsala Monitoring Centre (UMC), Sweden, promotes pharmacovigilance at the national level. The national data from the participating countries are shared with the UMC and compiled to generate a global ADR) database. The ADR database is collected from various countries under the WHOCC ADR monitoring programme and compiled together to generate signals or flags. A signal or flag is a presumed risk associated with the use of a drug and is supported by reliable data sources. Based on the signals or flags, recommendations are made for regulatory interventions at the international level in the form of labelling revisions or banning the drug and communicating the evident risks to the policymakers. India is one of the global partners in the global programme and participates under the Ministry of Health and Family Welfare via the Pharmacovigilance Programme of India (PvPI).

The PvPI is a WHO initiative to scrutinise drug-induced mortality and morbidity in India. The PvPI is a collaborative project between WHO and Central Drugs Standard Control Organization (CDSCO), Ministry of Health and Family Welfare in India to prevent the ADRs. Collection, reporting and follow-up of the ADRs occurring among the patients are prime activities included under PvPI. ADR monitoring centres (AMCs) are basic units of the PvPI, and it works at the national level intending to identify, analyse, characterise and estimate the extent of the problem associated with drug use. The programme aims to improve the vigilance on drugs, enhance patient safety and achieve better health benefits.^3 4^ Reporting ADRs at an institutional level provides valuable information about potential problems during drug usage in healthcare settings. However, the reports of signals or flags generated at UMC are not publicised to the AMCs. Moreover, there is a wide gap between the occurrence and reporting of ADRs globally.^5 6^ A study conducted in Norway concludes that ADRs can be prevented by collecting reliable information about their frequencies and possible risk factors.^7^ Thus, it is of utmost importance to collect and analyse the prevalence of ADR to: (A) identify the potential risks with drugs, (B) support management of diseases and (C) rationalise the prescribing practices.

## Objectives

The study was conducted to analyse and present the occurrence and severity of spontaneous ADR reports registered at an AMC in Central India.

## Methods

### Study design and setting

This non-interventional survey was a part of PvPI and presents the data collected from one of the AMC under PvPI. All ADR reports received from the hospitals and healthcare facilities between July 2013 and December 2019; at the AMC – R. D. Gardi Medical College, Ujjain, in Central India; were included in the study. The Medical College, established in the year 2000, is the first private medical college in the Madhya Pradesh state of India. The institute is recognised as an AMC since 2012.^8^

### Data collection considerations and analysis

The Suspected ADR Reporting form, recommended by the CDSCO, was used as the data collection tool (online supplemental annexure I).^9 10^ The suspected ADRs were spontaneously reported by the healthcare professionals (HCPs), patients or patient’s relatives to our AMC either directly or through the patient safety pharmacovigilance associate. All ADRs reported between 2013 and 2019 were included in the study.

10.1136/bmjopen-2021-052737.supp1 Supplementary data

The data were recorded and computerised in Microsoft Excel, using the protocols and formats provided by the National Coordination Centre of PvPI at the AMC. The data were analysed anonymously at the group level. The analyses related to the patients’ demography, frequency and route of administration of ADR causative drugs, organ system involved and identification of the unlabelled ADRs were done using the established tools.^11^ Appropriate descriptive statistics were used to analyse the quantitative data.

The causality of the reported adverse reactions was categorised into certain, probable, possible, unassessable/unclassifiable, unlikely and conditional/unclassified using the WHO causality assessment scale.^12^ This scale is used for the assessment of the relationship between a drug and the occurrence of an ADR. A serious reaction was characterised as a fatal, life-threatening reaction that can prolong hospitalisation and cause a significant persistent disability that might result in a congenital anomaly and require an intervention to prevent permanent damage or death. ADRs are categorised by Hartwig *et al* in seven levels based on their severity. Levels 1 and 2 falls under the mild category, whereas levels 3 and 4 are under the moderate category and levels 5, 6 and 7 fall under the severe category.^13^ The causal relationship between the suspected drug and the reaction was established using WHO–UMC standardised case causality assessment criteria.^12^

### Statistical methods

The data were analysed with Excel and STATA V.13.1 (Stata Corp). The frequencies and percentage of categorical values were calculated. Sum, median, mean, range and SD were calculated for the continuous numerical values. Percentages were rounded to the closest whole number. The independent t-test was used for the comparison of normally distributed and continuous variables. The χ^2^ test was used for comparison of categorical values, and p values <0.001 were considered as significant.

### Patient and public involvement statement

Patients or the public (family members of the patients) who noticed the ADR were involved to report the ADRs in our research. The ADRs were recorded in agreement with the patient under patient and public involvement. The person reporting an ADR could directly contact the pharmacovigilance team member and give the details of the ADR. The protocol of the collaborative project (WHO Collaborating Centre for International Drug Monitoring at Uppsala Monitoring Centre, Sweden) was followed.

### Ethics statement

The personal identification variables were masked before analyses, and analyses were conducted in groups to maintain full confidentiality. Since the results do not contain any personal information, taking consent from the patients was exempted. This was a non-interventional study where no influence was imposed on the treatment or medical care. Treating consultants were not approached regarding the reported ADRs as this was not the aim of the study. Full confidentiality was maintained throughout the analyses, and the analyses were performed in groups to affirm patients’ confidentiality. Necessary communication was accomplished with PvPI for permission to use the data for publication.

## Results

**Figure 1:**
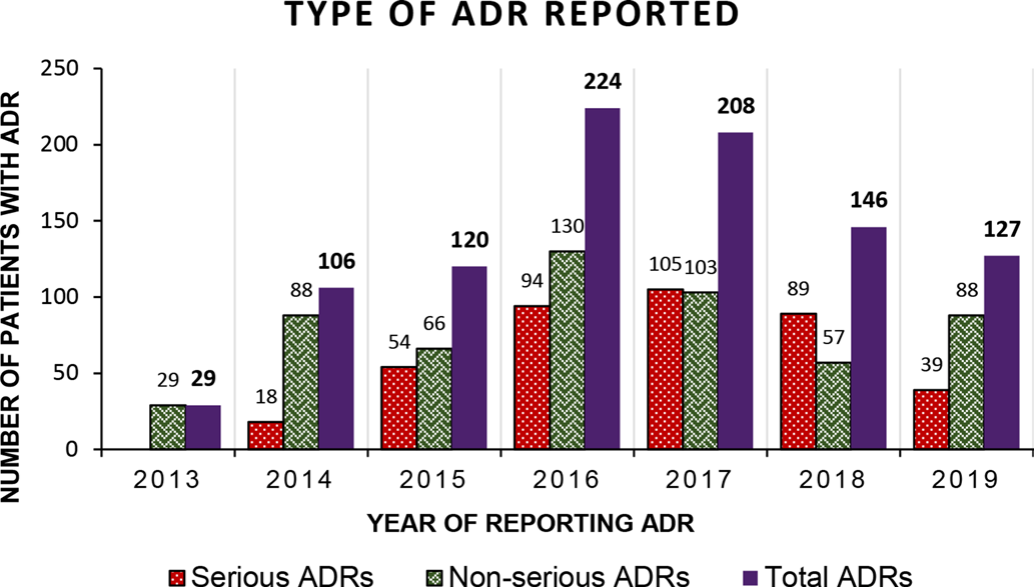
Frequency of type of ADRs reported per year at an AMC in Central India. ADRs, adverse drug reactions; AMC, ADR monitoring centre.

Spontaneous ADR reports were collected and analysed during 7 years at the AMC. A total of 1980 ADR reports were generated from 960 patients and were associated with 1316 prescriptions of drugs. The occurrence of ADRs dominated among males 64% (618) than female patients (table 1). Maximum number of ADRs were reported from the breadwinner group i.e., patients aged between 19 and 65 years (81%), and the overall mean age of the patients was 38.02848±0.5691212 years. A peak of ADR reports was attained in 2016 with 224 reports, which decreased to 127 in 2019. The highest number of ADRs were reported in the year 2016 (224), while the maximum number of serious ADRs were reported in the year 2017 (105, figure 1), and in the year 2018, the reports of serious ADRs exceeded the non-serious ADRs.

**Table 1:** Demographic parameter and route of drug administration among the patients developing ADRs at an AMC in Central India

Parameters	Number of Patients with ADRs, n (%)	P value
Total ADRs	**960**	
Age group (years)		
0–18	89 (9)	
19–65	773 (81)	0.0001
>65	98 (10)	
Sex		
Male	618 (64)	0.0001
Female	342 (36)	
Route of drug administration		
Oral	987 (75)	0.0001
Parenteral	308 (23)	
Topical	21 (2)	

**Figure 2:**
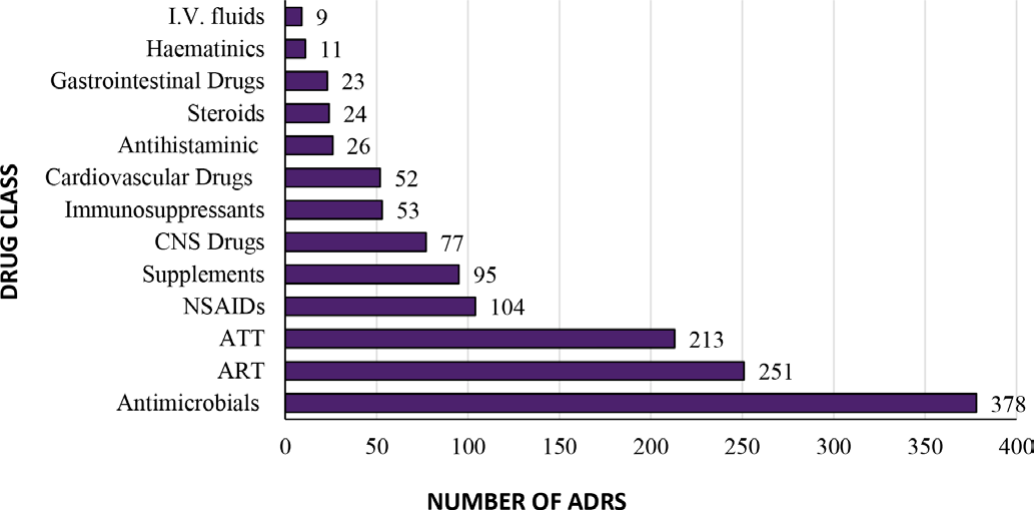
Occurrence of ADRs related to the pharmacological drug classes at an AMC in Central India. ADRs, adverse drug reactions; AMC, ADR monitoring centre; ART, antiretroviral therapy; ATT, antitubercular therapy; CNS, Central nervous system; NSAIDs, non-steroidal anti-inflammatory drugs.

**Figure 3:**
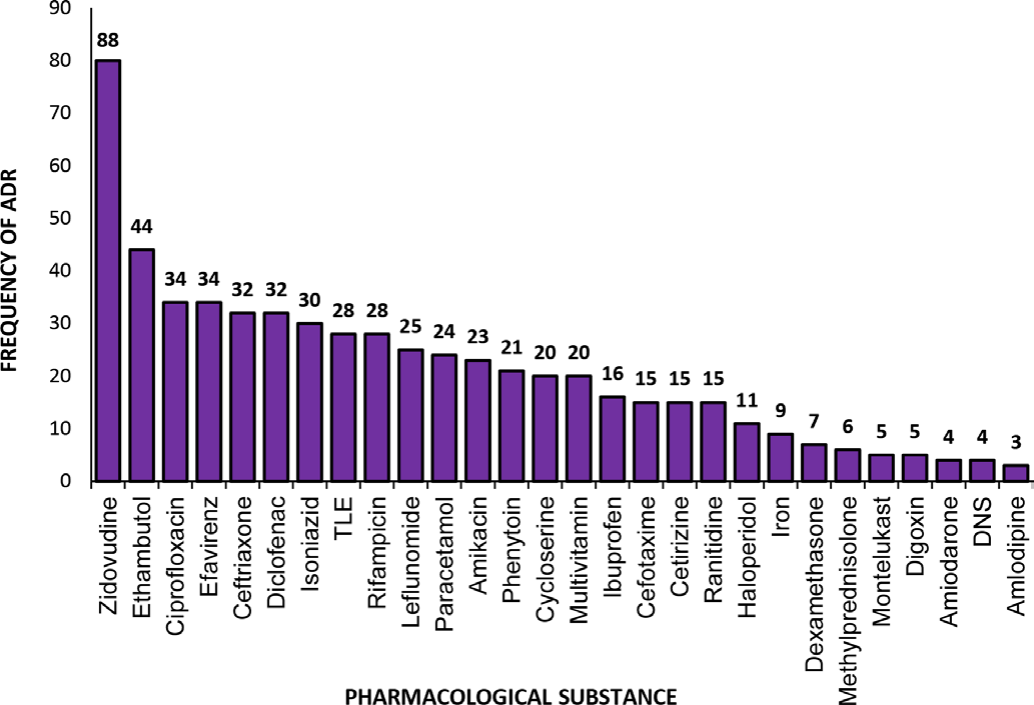
Pharmacological substances implicated in causing ADRs at an AMC in Central India. ADRs, adverse drug reactions; AMC, ADR monitoring centre; DNS, Dextrose Normal Saline; TLE, a combination of tenofovir, lamivudine and efavirenz.

ADRs, adverse drug reactions; AMC, ADR monitoring centre.;

Figure 2 summarises the number of ADRs associated with pharmacological drugs classes. In total, 1316 drugs were implicated in causing the ADRs, and antimicrobials were associated with the maximum number of the ADRs (29%) followed by the drugs of antiretroviral therapy (ART; 19%) and antitubercular therapy (ATT; 16%). The most common substances implicated for 88 ADRs were zidovudine followed by efavirenz (34 ADRs), ethambutol (44 ADRs) and ciprofloxacin (34 ADRs, figure 3).

Table 2 presents the types of ADRs reported in various system organ classes (SOCs). The ADRs of skin and subcutaneous tissue disorders (28%) were most common among all SOCs followed by gastrointestinal systems (18%).

**Table 2:** Frequency of ADR symptoms based on the involvement of organ system at an AMC in Central India

System organ class	ADR symptoms n (%) 1980 (100)	Most common ADRs (n)
Skin and subcutaneous tissue disorder	548 (28)	Rash (231), generalised pruritus (113), fixed drug eruption (64), burning sensation (19)
Gastrointestinal disorders	365 (18)	Vomiting (91), nausea (87), abdominal discomfort (71), diarrhoea (52)
Nervous system disorder	219 (11)	Dizziness (61), headache (50), paraesthesia (17), peripheral neuropathy (12)
General disorders	193 (10)	Multi-drug resistance (35), Shivering (33), Generalised oedema (21), Weakness generalised (18)
Psychiatric disorder	129 (7)	Anxiety (33), sedation (24), drowsiness (17), psychosis (9)
Musculoskeletal and connective tissue disorders	82 (4)	Myalgia (29), tremors (17), joint pain (7)
Respiratory disorder	64 (3.2)	Cough (18), breathing difficulty (16), dyspnoea (14)
Blood and lymphatic system disorder	60 (3)	Anaemia (47), thrombocytopaenia (6)
Cardiac disorder	45 (2)	Tachycardia (19), chest pain (16), bradycardia (5)
Hepatobiliary disorder	41 (2)	Hepatotoxicity (39)
Nutritional and metabolic disorder	40 (2)	Loss of appetite (28), pallor (3), weight gain (3)
Ear disorder	31 (2)	Impaired hearing (14), tinnitus (8), vertigo (8)
Vascular disorder	31 (2)	Hypotension (16), epistaxis (4), hypertension (3)
Eye disorder	26 (1)	Impaired vision (16), eye irritation (2), lacrimation (2)
Injury, poisoning and procedural complications	25 (1)	Injection site reaction (12), extravasation (6)
Renal disorder	25 (1)	Increased serum creatinine (6), haematuria (4), dysuria (3)
Endocrine disorders	23 (1)	Gynaecomastia (8), moon face (5), hypoglycaemia (3)
Immune system disorder	19 (1)	Allergic dermatitis (8), allergic reactions (4), anaphylactic reaction (4)
Electrolyte imbalance	7 (0.35)	Hypokalemia (2), hypocalcaemia (1), hyperuricaemia (2)
Reproductive and breast disorder	7 (0.35)	Vaginal haemorrhage (2), breast tenderness (1)

ADR, adverse drug reaction; AMC, ADR monitoring centre.;

Table 3 shows the list of drugs that were most frequently associated with the ADR symptoms. Leflunomide followed by gliclazide, and paracetamol caused rashes. A fixed-dose combination of antibiotics, cefoperazone with sulbactam was most commonly associated with generalised pruritus. Zidovudine caused anaemia, and the combination of tenofovir, lamivudine and efavirenz caused gynaecomastia. Antibiotics were tagged as suspected drugs for the ADRs in 204 reports. The most common indications for prescribing antibiotics among the 204 cases were multidrug-resistant tuberculosis (34 ADRs), bacterial infections (20 ADRs) and surgical prophylaxis (18 ADRs). Most patients were resistant to the fixed-dose combination of antibiotics, that is, ceftriaxone and sulbactam. In a majority of the cases (99%), antibiotics were prescribed either as postsurgical prophylaxis to the patients specified with a surgical procedure or to the trauma patients or patients with unclean and infected wounds as prophylaxis for suspicion of bacterial infection.

**Table 3:** Most frequently associated drugs with ADR symptoms at an AMC in Central India

ADR symptom (n)	Name of the drug, suspected for the ADR symptom (n)
Rash (231)	Leflunomide (40), gliclazide (21), paracetamol (20)
Generalised pruritus (113)	Cefoperazone with sulbactam (12), cefotaxime (8), levofloxacin (8), diclofenac (7)
Vomiting (91)	Tenofovir (22), quinine (19)
Nausea (87)	Zidovudine (23), TLE (18), quinine (11)
Abdominal pain (71)	Zidovudine (9), aspirin (8), leflunomide (4)
Dizziness (61)	TLE (21), ranitidine (7), quinine (6)
Anaemia (47)	Zidovudine (43)
Hepatotoxicity (39)	ATT (21)
Multidrug resistance (35)	Ceftriaxone with tazobactam (15), amikacin (9)
Anxiety (33)	TLE (12), quinine (7)
Myalgia (29)	Zidovudine (173)
Injection site reaction (12)	Artesunate (3), cefotaxime (3), ceftriaxone (2)
Gynaecomastia (8)	TLE (7)
Increased serum creatinine (6)	Tenofovir (5)

ADR, adverse drug reaction; AMC, ADR monitoring centre; ATT, antitubercular treatment; TLE, a combination of tenofovir, lamivudine and efavirenz.

Table 4 shows the severity and causality assessment of reported ADRs. Four per cent of all reported ADRs were severe, and the rest were mild to moderate. Overall, 26 drugs were associated with 36 serious ADRs. Electrolyte imbalance and reproductive and breast disorders caused seven ADR cases, while immune system disorder caused 19 ADR cases. The outcome was fatal in 12 ADR episodes, while 13 recovered with sequelae, 774 cases (55%) either recovered (521) or were recovering (253) at the time of ADR reporting and 90 were not having any sign of recovery until the ADR was reported.

**Table 4:** Assessment of severity and causality of the ADRs reported at an AMC in Central India

Type of severity (Hartwig Scale)^8^	ADR, n (%)
Total	960
Mild	564 (59)*
Moderate	360 (37.5)
Severe	36 (4)
Causality assessment (WHO Scale)^9^	
Certain	93 (5)
Probable/likely	846 (43)
Possible	951 (48)
Unlikely	22 (1)
Inaccessible/unclassifiable	68 (3)

n=number of ADRs.

*Statistically significant p value >0.0001.

ADRs, adverse drug reactions.

## Discussion

There is a wide gap between the occurrence and reporting of ADRs in India and worldwide. The present study illustrates the ADRs reported at an AMC in Central India for the first time and present a list of the causative drugs for the burden of ADRs. The study highlighted antimicrobials as the most common causative drugs for a high burden of ADRs followed by the ART drugs and the ATT drugs at the AMC. The occurrence of ADRs was more common in male patients (64%) and among patients of the breadwinner age group (19–65 years, 81%). Zidovudine was the agent that caused most ADRs (88) followed by ethambutol, ciprofloxacin and efavirenz.

Reporting of spontaneous ADRs is a collaborative study conducted with the help of inputs from healthcare providers or a person close to the patients. The maximum number of ADRs were reported in the year 2016 (224). The number of reported ADRs decreased after achieving a peak in the year 2016. The maximum number of serious ADRs were reported in 2017 and the non-serious ADRs in 2016. Although the data collection started in July 2013, yet the number of reported ADRs is lower for half a year. These low numbers might be due to the initial stage of the study when the collaborators were being sensitised for data collection. However, the reduction in the number of reported ADRs from 2016 onwards must act as a stimulus to plan future studies to identify the factors responsible for under-reporting including qualitative method of data collection. Extensive efforts are needed for the corrective measures to improve the reporting. In our study, significantly more ADRs were documented in the male patients than the female patients, which are consistent with the earlier studies,^14 15^ whereas few studies have also reported female preponderance.^16 17^ However, the gender impact on the ADR occurrence cannot be explained here and might be an incidental finding and has no effect on the occurrence of reporting of the ADRs. In our study, the ADRs were predominantly reported in patients of age group between 19 and 65 years, which is also similar to a study conducted by Daulat *et al*^14^ and Thakare *et al*.^17^ This is because it is a wide age group range, and this is likely the major population that attends hospital more frequently and receives drug therapy.

The results based on pharmacovigilance data are crucial to generate signals and alerts. However, signals are generated based on a substantial number of reports after following the protocol. Yet, the frequencies of ADRs reported by some of the drugs in the present study are alarming. The pharmacological drug classes implicated for causing ADRs shows that antimicrobials including antibiotics caused the maximum number of ADRs (29%). This might be because antimicrobials are one of the most prescribed drug classes specifically in LMICs.^18 19^ It is also documented that this class is prescribed for unindicated conditions providing an opportunity to develop antimicrobial resistance.^15 20–22^ The ADR reports associated with antibiotics can facilitate the development of appropriate policies and guidelines for the judicious use of antibiotics and thereby combat antimicrobial resistance.^23^ A fixed-dose combination of cefoperazone with sulbactam was most commonly associated with generalised pruritus. Among 35 cases of multidrug resistance, most cases were encountered with a fixed-dose combination of ceftriaxone and sulbactam followed by amikacin. Judicious selection of antibiotics based on the type of infection, local resistance pattern and prescribing guidelines is recommended.^24^ However, in our study, the antibiotics were prescribed empirically as prophylaxis in a majority of the cases for suspicion of bacterial infection.

Initiating the treatment by prescribing antibiotics empirically with an intent to send samples for antibiotic susceptibility test (AST) and to modify the treatment based on the laboratory results is a standard procedure. However, the poor practice of sending samples for AST is a global issue and has also been reported earlier from the study settings.^24 25^ Moreover, prescribing broad-spectrum antibiotics empirically and for unindicated conditions is one of the major causes of developing antibiotic resistance and has also been reported from the setting.^18 24 26^ It is noteworthy that in the present study, the AST samples were sent mostly when the patient do not respond to the prescribed antibiotics. A few ADR reports specify that antibiotic prescriptions were modified based on the AST reports. The most common shifts were made from the combination of ceftriaxone with tazobactam to meropenem or linezolid. However, the availability of too few records limits us to comment any further. Therefore, we suggest that every attempt should be made to encourage maximum use of laboratories to narrow down the antibiotic spectrum and minimise the risk of antibiotic resistance.

Besides antibiotics, the other commonly prescribed pharmacological drug classes implicated for ADRs were ART, ATT, non-steroidal anti-inflammatory drugs (NSAIDs), Central nervous system (CNS) and Cardiovascular system (CVS) drugs in descending order of causing ADRs. The ART and ATT drugs, despite their efficacy, are continuously found associated with a wide range of potential adverse effects.^27^ The occurrence of ADRs related to ART and ATT drugs may result in poor compliance with the treatment that can lead to the development of resistance and treatment failure including virologic failures.^28–30^ No common drug class could be implicated for causing serious ADRs events.

In the present study, the most common drugs causing rash were leflunomide followed by gliclazide and paracetamol. While zidovudine was most notorious for already labelled as associated ADRs such as anaemia, myalgia, nausea and abdominal pain. Anaemia is found to be associated with both the dose and stage of disease, and myopathy is the most significant yet manageable type of late adverse reaction.^31^

The combination of tenofovir, lamivudine and efavirenz was most commonly associated with vomiting, dizziness, anxiety and gynaecomastia. Among these ADRs, association with dizziness draws attention. It has been found that those patients who did not report such ADRs to the attending physicians end up having severe diseases. The debilitating nature of the symptoms and awareness levels of the patients about, what should report to the attending doctor, plays an important role in reporting ADRs. Therefore, increasing awareness of patients is of utmost importance via asking direct questions about the ADRs caused by the flagged drugs.^32 33^ In addition, a plan could be developed to disseminate the findings of the pharmacovigilance surveillance to the prescribers so that they can avoid prescribing the drugs responsible for ADRs, wherever possible. In cases where it is not possible to avoid the drug, for example, in ART, the prescribers can counsel patients based on the associations found via pharmacovigilance. This will help to improve patient’s compliance and will motivate patients to seek early advice in case of occurrence of ADRs.

Polypharmacy is prevalent globally, and the occurrence of ADR is directly proportional to the number of prescribed drugs; thus, polypharmacy must be discouraged.^34 35^ However, in the present study single drug was attributed for most of the ADRs (73%). It was observed that in most of the reports the drug that was most suspected of being associated with the specific ADR was reported and not the whole treatment. NSAIDs are often a constituent of many rational and irrational fixed-dose combinations, and this could be a cause of the high incidence of the ADRs related to the NSAIDs.^36^ This was reflected in our study where besides antimicrobials and ART drugs, the antiepileptics and NSAIDs also caused substantial ADRs.^19–22 37–41^ In the present study, the most prescribed pharmacological substances among the patients having ADRs were zidovudine, ethambutol and ciprofloxacin, while in other studies, ceftriaxone, zidovudine and phenytoin sodium were the most prescribed drugs.^37–41^ Zidovudine and ethambutol are part of multidrug ATT and ART regimes, respectively. Factors including the dose of the drugs, drug–drug interactions as well as genetic differences of the study population could also affect the number of ADRs caused by drugs.^42^

Most of the patients had mild ADRs in our study, as observed in other Indian studies.^15 17^ ADRs of skin and subcutaneous tissue disorders (28%) were most common among all SOCs. Skin rashes were the most common complaints followed by gastrointestinal complaints (18%), nervous system disorders (11%), general disorders (10%) and psychiatric disorders (7%). Skin and subcutaneous tissues are among the most targeted organs for ADRs. The disorders are reported more frequently because these develop within 1 week of drug administration and can easily be spotted when the patients are still under the close supervision of the healthcare providers.^15 43 44^ Timely recognition of skin ADRs is crucial for its classification, management and causality assessment.

The causality assessment was conducted based on the WHO probability scale. According to the assessment, most cases could be categorised as ‘possible’ (48) followed by ‘probable’ (43). ADRs are rarely categorised as ‘certain’ as it requires to rechallenge the patient with the same causative drug, which is not ethical, hence the assessment frequently goes to the possible and probable category.^15 45^ Though 26 drugs were associated with serious, life-threatening ADRs, yet none of the drug classes could be implicated for serious ADRs. This was because of insufficient data due to the under-reporting of ADRs and the wide range of drugs prescribed. An adequate ADR reporting is required to preserve the clinical information that could prevent significant damage to patients, plan and implement measures to reduce their impact on public health and consequently minimise the burden on the healthcare system. Under-reporting is a common challenge in pharmacovigilance. This was clearly reflected in our study; when other AMCs contributed with an average of 212 ADR reports per month, our AMC reported an average of 19 ADRs per month in 2016 which dropped to 11 in 2019.^46^

Early identification and management of ADRs will improve patient safety and quality of life and reduce the cost of treatment. Labelling of new ADRs will help to prepare the prescribing policies and modify the prescribing patterns. Reduction in prescribing of the flagged drugs will reduce the prevalence of ADRs. We believe that disseminating results to the prescribers through feedback might reduce prescribing of causative drugs and improve reporting of ADRs. A knowledge, attitude and practice-based study conducted in Norway found that ADRs can be prevented by collecting reliable information about their frequencies and possible risk factors.^7^ Therefore, we propose to develop and implement strategies for the community but specifically for the HCWs to improve reporting and monitoring of ADRs. We also propose to disseminate the study results in the form of a feedback method to improve reporting. This might also help in the reduction of prescription of the drugs causing most of the ADRs. The methods of the feedback study could be shared and tested at other similar settings as well.

## Conclusion

The maximum number of ADRs were reported from the breadwinner group (males and 19–65 years). Antimicrobials caused the maximum number of ADRs. This highlights an alarming situation in addition to antimicrobial resistance. Prudent use of antimicrobials is once again proven as need of the hour. ADRs of skin and subcutaneous tissue disorders were most frequent among all SOCs. Most of the ADRs were mild and belonged to the ‘possible’ group of probability scale. However, under-reporting of ADRs at our AMC is evident and a major factor for the pause in performing severity and causality assessment. Thus, the prevalence of ADRs reported in the present study can be perceived as the tip of an iceberg.

To deal with the problem of under-reporting, we suggest continuous training programmes for HCPs, seeking the collaboration of nearby healthcare facilities and most importantly giving feedback to the HCWs to impart confidence, awareness and knowledge about the ADR causing drugs and will encourage reporting ADR events.

### Future implications

Our results indicate the need to develop and test a model of motivational educational- approach focusing on healthcare workers and the community to increase ADR reporting in the settings associated with our AMC. Neither the global pharmacovigilance nor the PvPI has designed a plan to analyse and provide feedback on the ADR data to the prescribers. Providing reliable and balanced information and a valid assessment of the risk–benefit profile of the marketed drugs to the HCPs at a local level is essential. This knowledge might improve patients’ safety while prescribing drugs, support patient care and public health programmes in the settings. Therefore, we propose to develop a model with a strategy to provide feedback and knowledge to improve ADR reporting. Furthermore, providing feedback to the prescribers and sensitising them about the flagged drugs will be an encouragement for reporting ADRs. HCPs will be assured that reporting ADRs has no legal implications.

Modification of the organisational structure, training of HCPs and availability of resources can improve early detection of ADRs. We also suggest planning and start an ADR awareness and training campaign in collaboration with HCPs of the hospitals in the periphery, district and private hospitals to motivate them to report ADRs. However, the long-term sustainability of these measures is most crucial as the effectiveness of interventions diminishes with time. The use of a digital platform such as Short Message Service (SMS) and e-mails might play an important role in reminding and keeping them motivated and sensitised to report ADRs timely.

## Supplementary Material

Author's manuscript

## Data Availability

Data are available on reasonable request. As per the protocol of the institutional ethics committee to maintain full confidentiality, the data can be made available through a request form addressing, The Chairman, Ethics Committee, R.D. Gardi Medical College, Agar Road, Ujjain, Madhya Pradesh, India 456006 (email: iecrdgmc@yahoo.in, uctharc@sancharnet.in), giving all details of the article. Quote the IEC approval number: 68/2019 along with the request.
