# Supplementary material for: Surveillance of adverse drug reactions at an adverse drug reaction monitoring centre in Central India: a 7-year surveillance study
Source: BMJ Open. 2021 Oct 3;11(10):e052737. doi: 10.1136/bmjopen-2021-052737 (PMC8491296; doi:10.1136/bmjopen-2021-052737)
Supplement: Supplementary data [file bmjopen-2021-052737supp001.pdf]

ANNEXURE – I

Version-1.3

SUSPECTED ADVERSE DRUG REACTION REPORTING FORM

For VOLUNTARY reporting of Adverse Drug Reaction by Healthcare Professionals

INDIAN PHARMACOPOEIA COMMISSION (National Coordination Centre-Pharmacovigilance Programme of India)
Ministry of Health & Family Welfare, Government of India Sector-23, Raj Nagar, Ghaziabad-201002

A. PATIENT INFORMATION

1. Patient Initials ________	2. Age at the time of Event or Date of Birth ____________	3. M ☐ F ☐ Other ☐<br>4. Weight________Kgs

Reg. No. /IPD No. /OPD No. /CR No. :
AMC Report No. :
Worldwide Unique No. :

B. SUSPECTED ADVERSE REACTION

5. Event/Reaction start date (dd/mm/yyyy)
6. Event/Reaction stop date (dd/mm/yyyy)
6 (A). Onset Lag Time
7. Describe Event/Reaction with treatment details, if any

12. Relevant tests/ laboratory data with dates

13. Relevant medical/medication history (e.g. allergies, race, pregnancy, smoking, alcohol use, hepatic/renal dysfunction, past surgery etc.)

14. Seriousness of the reaction: No ☐ if Yes ☐(please tick anyone)
☐ Death (dd/mm/yyyy) ☐ Congenital-anomaly
☐ Life threatening ☐ Disability
☐Hospitalization/Prolonged ☐ Other Medically important

15. Outcomes
☐ Recovered ☐ Recovering ☐ Not recovered
☐ Fatal ☐ Recovered with sequelae ☐ Unknown

C. SUSPECTED MEDICATION(S)

S.No	8. Name (Brand/Generic)	Manufacturer (if known)	Batch No. / Lot No.	Exp. Date (if known)	Dose used	Route used	Frequency (OD, BD etc.)	Therapy dates		Indication	Causality Assessment
								Date started	Date stopped		
i											
ii											
iii											
iv*											

S.No as per C	9. Action Taken (please tick)						10. Reaction reappeared after reintroduction (please tick)			
	Drug withdrawn	Dose increased	Dose reduced	Dose not changed	Not applicable	Unknown	Yes	No	Effect unknown	Dose (if reintroduced)
i										
ii										
iii										
iv										

11. Concomitant medical product including self-medication and herbal remedies with therapy dates (Exclude those used to treat reaction)

S.No	Name (Brand/Generic)	Dose used	Route used	Frequency (OD, BD, etc.)	Therapy dates		Indication
					Date started	Date stopped	
i							
ii							
iii*							

Additional Information:

D. REPORTER DETAILS

16. Name and Professional Address:_______________________
____
Pin:___________E-mail_____________________________
Tel. No. (with STD code)___________________________
Occupation:__________________ Signature:_______________

17. Date of this report (dd/mm/yyyy):

Sig. and Name of Receiver-

Confidentiality: The patient's identity is held in strict confidence and protected to the fullest extent. Submission of a report does not constitute an admission that medical personnel or manufacturer or the product caused or contributed to the reaction. Submission of an ADR report does not have any legal implication on the reporter.

*use separate page for more information

Sharma M, et al. BMJ Open 2022; 11:e052737. doi: 10.1136/bmjopen-2021-052737
