# Supplementary material for: Surveillance of adverse drug reactions at an adverse drug reaction monitoring centre in Central India: a 7-year surveillance study
Source: BMJ Open. 2021 Oct 3;11(10):e052737. doi: 10.1136/bmjopen-2021-052737 (PMC8491296; doi:10.1136/bmjopen-2021-052737)
Supplement: Author's manuscript [file bmjopen-2021-052737.draft_revisions.pdf]

BMJ Open is committed to open peer review. As part of this commitment we make the peer review history of every article we publish publicly available.

When an article is published we post the peer reviewers' comments and the authors' responses online. We also post the versions of the paper that were used during peer review. These are the versions that the peer review comments apply to.

The versions of the paper that follow are the versions that were submitted during the peer review process. They are not the versions of record or the final published versions. They should not be cited or distributed as the published version of this manuscript.

BMJ Open is an open access journal and the full, final, typeset and author-corrected version of record of the manuscript is available on our site with no access controls, subscription charges or pay-per-view fees (http://bmjopen.bmj.com).

If you have any questions on BMJ Open's open peer review process please email info.bmjopen@bmj.com

BMJ Open

Surveillance of adverse drug reactions at an adverse drug reaction monitoring centre in Central India: A seven-year surveillance study

Journal:	*BMJ Open*
Manuscript ID	bmjopen-2021-052737
Article Type:	Original research
Date Submitted by the Author:	26-Apr-2021
Complete List of Authors:	Sharma, Megha; Karolinska Institutet, Department of Global Public Health; RD Gardi Medical College, Department of Pharmacology<br>Baghel, Ruchi; RD Gardi Medical College, Department of Pharmacology<br>Thakur, Sunil; RD Gardi Medical College, Department of Pharmacology<br>Adwal, Sandeep; Zydus Medical College and Hospital, Department of Pharmacology
Keywords:	CLINICAL PHARMACOLOGY, Health & safety < HEALTH SERVICES ADMINISTRATION & MANAGEMENT, Risk management < HEALTH SERVICES ADMINISTRATION & MANAGEMENT, Adverse events < THERAPEUTICS, Toxicity < THERAPEUTICS, Pharmacology < TROPICAL MEDICINE

SCHOLARONE™
Manuscripts

I, the Submitting Author has the right to grant and does grant on behalf of all authors of the Work (as defined in the below author licence), an exclusive licence and/or a non-exclusive licence for contributions from authors who are: i) UK Crown employees; ii) where BMJ has agreed a CC-BY licence shall apply, and/or iii) in accordance with the terms applicable for US Federal Government officers or employees acting as part of their official duties; on a worldwide, perpetual, irrevocable, royalty-free basis to BMJ Publishing Group Ltd ("BMJ") its licensees and where the relevant Journal is co-owned by BMJ to the co-owners of the Journal, to publish the Work in this journal and any other BMJ products and to exploit all rights, as set out in our licence.

The Submitting Author accepts and understands that any supply made under these terms is made by BMJ to the Submitting Author unless you are acting as an employee on behalf of your employer or a postgraduate student of an affiliated institution which is paying any applicable article publishing charge ("APC") for Open Access articles. Where the Submitting Author wishes to make the Work available on an Open Access basis (and intends to pay the relevant APC), the terms of reuse of such Open Access shall be governed by a Creative Commons licence – details of these licences and which Creative Commons licence will apply to this Work are set out in our licence referred to above.

Other than as permitted in any relevant BMJ Author's Self Archiving Policies, I confirm this Work has not been accepted for publication elsewhere, is not being considered for publication elsewhere and does not duplicate material already published. I confirm all authors consent to publication of this Work and authorise the granting of this licence.

For peer review only

Surveillance of adverse drug reactions at an adverse drug reaction monitoring centre in Central India: A seven-year surveillance study

Authors: Megha Sharma[#*1,2], Ruchi Baghel*[1], Sunil Thakur[1], Sandeep Adwal[3]

[1]Department of Global Public Health- Health Systems and Policy: Medicines, Karolinska Institutet, Stockholm, Sweden. 171 77

[2]Department of Pharmacology, R. D. Gardi Medical College, Ujjain, Madhya Pradesh, India. 456006

[3]Department of Pharmacology, Zydus Medical College and Hospital, Nimnaliya, Dahod, Gujarat. 389151

*Contributed equally

[#] Corresponding author

Email:

Megha Sharma: megha.sharma@ki.se

Ruchi Baghel: drruchibaghel@gmail.com

Sunil Thakur: sunilcsthakur@gmail.com

Sandeep Adwal: adwalsandeep@yahoo.co.in

Corresponding author

[#] Associate Professor, Department of Pharmacology

R. D. Gardi Medical College Ujjain, (MP), India

ZIP/PIN 456006

Keywords: Pharmacovigilance, Adverse drug reactions, ADR Monitoring Centre, spontaneous ADRs, Antimicrobials, Antiretroviral therapy, Antitubercular therapy, India

For peer review only - http://bmjopen.bmj.com/site/about/guidelines.xhtml

Abstract

Objectives

To analyse and present the occurrence and severity of spontaneous ADRs reports prospectively registered at an AMC in Central India.

Setting and data

The survey was conducted between 2013 and 2019 at an ADR Monitoring Centre in Central India. ADRs were recorded using the standard 'Suspected ADR Reporting form'.

Outcome measures: The causality of the ADRs were categorized using WHO causality assessment scale to assess the relationship between a drug and occurrence of an ADR.

Results

Totally 1980 spontaneous ADRs were reported involving 960 patients and 1316 drugs prescriptions. The occurrence of ADRs was common among male patients (64%) and patients of age between 19-65 years (81%). Antimicrobials caused 29% ADRs, followed by drugs of Antiretroviral therapy (19%). Zidovudine caused most ADRs (88%) followed by ethambutol and ciprofloxacin. The ADRs of skin and subcutaneous tissue disorders (28%) were most common among all system-organ classes followed by gastrointestinal systems (18%). Four percent of all reported ADRs were severe. A peak of ADR reports was attained in 2016 with 224 reports which decreased to 127 in 2019.

Conclusion

A high number of ADRs caused by antimicrobials is an alarming situation, which adds up to antimicrobial resistance. Judicious use of antimicrobials is yet again proven as need of the hour. Under-reporting of ADRs is evident in our study and is a major factor for the delay in withdrawal of drugs responsible for causing ADRs. Interventions in terms of training and feedback are suggested to encourage and improve ADR reporting.

For peer review only - http://bmjopen.bmj.com/site/about/guidelines.xhtml

Article summary

Strengths and limitations of this study

- This is the first long term study that presents the ADRs reported at an AMC in Central India.
- This study presents a list of the drugs most notoriously causing ADRs.
- The results of the study will be used to provide feedback and training to improve the under-reporting of ADRs.
- The precise incidence of suspected ADRs due to a particular drug could not be estimated due to under-reporting.
- The data was collected based on spontaneous reporting rather than active surveillance, which would have been a better method.

Word count 3134

For peer review only - http://bmjopen.bmj.com/site/about/guidelines.xhtml

Background

Pharmacovigilance is a process of post-marketing surveillance of drugs–that continues throughout the drug lifecycle. It is essential in analysing and managing the risks associated with drugs that are available for the use of the general population [1]. The results of pharmacovigilance certify the effectiveness and safety of drugs in terms of adverse drug reactions (ADRs). Occurrence of ADRs can prolong hospitalization and thereby increase cost of hospital stay and treatment and initiate the requirement of additional clinical investigations in severe cases. World health organization (WHO) defines 'Pharmacovigilance' as the science and activities relating to the detection, assessment, understanding, and prevention of adverse effects or any other possible drug-related problems, including herbal medicines [2]. The WHO Collaborating Centre (WHOCC) for International Drug Monitoring at Uppsala Monitoring Centre (UMC), Sweden; promotes pharmacovigilance at the national level. The national data from the participating countries are shared with the UMC and compiled to generate a global adverse drug reaction (ADR) database. The ADR database is collected from various countries under the WHOCC ADR monitoring program and compiled together to generate signals or flags. A signal or flag is a presumed risk associated with the use of a drug and is supported by reliable data sources. Based on the signals or flags, recommendations are made for regulatory interventions at the international level in the form of labelling revisions or banning the drug and communicating the evident risks to the policymakers. India is one of the global partners in the global program and participates under the Ministry of family welfare via the Pharmacovigilance Programme of India (PvPI).

The PvPI is a WHO initiative to scrutinize drug-induced mortality and morbidity in India. The PvPI is a collaborative project between WHO and Central Drugs Standard Control Organization (CDSCO), Ministry of Health and Family Welfare in India to prevent the ADRs. Collection, reporting and follow-up of the adverse drug reactions (ADRs) occurring among the

For peer review only - http://bmjopen.bmj.com/site/about/guidelines.xhtml

patients are prime activities included under PvPI. ADR Monitoring Centres (AMCs) are basic units of the PvPI, and it works at the national level intending to identify, analyse, characterize, and estimate the extent of the problem associated with drug use. The program aims to improve the vigilance on drugs, enhance patient safety and achieve better health benefits [3,4]. Reporting ADRs at an institutional level provide valuable information about potential problems during drug usage in healthcare settings. However, the reports of signals or flags generated at UMC are not publicized to the AMCs. Moreover, there is a wide gap between the occurrence and reporting of ADRs globally [5,6]. A study conducted in Norway concludes that the ADRs can be prevented by collecting reliable information about their frequencies and possible risk factors. [7] Thus, it is of utmost importance to collect and analyse the prevalence of ADR to; a) identify the potential risks with drugs, b) support management of diseases and, c) rationalise the prescribing practices.

Objectives

The study was conducted to analyse and present the occurrence and severity of spontaneous ADRs reports registered at an AMC in Central India.

Methods

Study design and setting

This non-interventional survey was a part of PvPI and presents the data collected from one of the AMC under PvPI. All ADR reports received from the hospitals and healthcare facilities between July 2013 and December 2019, at the AMC- R. D. Gardi Medical College, Ujjain in Central India were included in the study. The Medical College, established in the year 2000, is the first private medical college in the Madhya Pradesh state of India. The institute is recognised as an AMC since 2012 [8].

For peer review only - http://bmjopen.bmj.com/site/about/guidelines.xhtml

Data collection considerations and analysis

The Suspected ADR Reporting form, recommended by the CDSCO was used as the data collection tool (Annexure I) [9,10]. The suspected ADRs were spontaneously reported by the Health Care Professionals (HCPs), non-HCPs or patients; to our AMC either directly to the authors or through a Patient Safety Pharmacovigilance Associate (ST). All ADRs reported between 2013 and 2019 were included in the study.

The data was recorded and computerised in Microsoft Excel, using the protocols and formats provided by the National Coordination Centre of PvPI at the AMC. The data were analysed anonymously, at the group level. The analyses related to the patients' demography, frequency, and route of administration of ADR causative drugs, organ system involved and identification of the unlabelled ADRs were done using the established tools [11]. Appropriate descriptive statistics were used to analyse the quantitative data.

The causality of the reported adverse reactions was categorized into certain, probable, possible, un-assessable/unclassifiable, unlikely, conditional/unclassified using the WHO causality assessment scale [12]. This scale is used for the assessment of the relationship between a drug and the occurrence of an ADR. A serious reaction was characterized as a fatal, life threatening reaction that can prolong hospitalization, and cause a significant persistent disability, might result in a congenital anomaly, and require an intervention to prevent permanent damage or death. ADRs are categorised by Hartwig SC et. al. in seven levels based on their severity. Level 1 and 2 falls under the mild category whereas level 3 and 4 under the moderate category and level 5, 6 and 7 fall under the severe category [13]. The causal relationship between the suspected drug and the reaction was established using WHO–UMC standardized case causality assessment criteria [12].

For peer review only - http://bmjopen.bmj.com/site/about/guidelines.xhtml

Statistical methods

The data were analysed with Excel, and STATA V.13.1 (Stata Corp, College Station, Texas, USA). The frequencies and percentage of categorical values were calculated. Sum, median, mean, range and SD were calculated for the continuous numerical values. percentages were rounded to the closest whole number. The independent t-test was used for comparison of normally distributed and continuous variables. The $\chi 2$ test was used for comparison of categorical values and p values <0.001 were considered as significant.

Patient and Public Involvement statement

Patients or the public (the patient or family members or a healthcare worker who noticed the ADR) WERE involved to report the ADRs in our research. The ADRs were recorded with the consent of the patient under Patient and Public Involvement. The person reporting an ADR could directly contact the pharmacovigilance team member and give the details of the ADR. The protocol of the collaborative project (WHO Collaborating Centre for International Drug Monitoring at Uppsala Monitoring Centre, Sweden) was followed.

Ethics statement

The study was approved by the Institutional Ethics Committee of Ruxmaniben Deepchand Gardi Medical College, Ujjain, with approval number IEC Ref. No -68/2019. Consent was taken from the patients, prior to data collection. Moreover, the personal identification variables were masked before analyses and analyses were conducted in groups to maintain full confidentiality. Therefore, the results do not contain any personal information. This was a non-interventional study where no influence was imposed on the treatment or medical care. Treating consultants were not approached regarding the reported ADRs as this was not the aim of the

For peer review only - http://bmjopen.bmj.com/site/about/guidelines.xhtml

study. Full confidentiality was maintained throughout the analyses and the analyses were performed in groups to affirm patients' confidentiality.

Results

Spontaneous ADR reports were collected and analysed during seven years at the AMC. A total of 1980 ADR reports were generated from 960 patients and were associated with 1316 prescriptions of drugs. The occurrence of ADRs dominated among males 64% (618) than female patients (Table 1). The maximum number of ADRs were reported were from the breadwinner group patients ageing between 19 and 65 years (81%), and the mean age of the patients was 38.02848 ± 0.5691212. A peak of ADR reports was attained in 2016 with 224 reports which decreased to 127 in 2019. The highest number of ADRs were reported in the year 2016 (224) while the maximum number of serious ADRs were reported in the year 2017 (105, Figure 1) and in the year 2018, the reports of serious ADRs exceeded the non-serious ADRs.

Table.1 Demographic parameter and route of drug administration among the patients developing ADRs at an AMC in Central India

Parameters	Number of Patients with ADRs, n (%)	P-value
**Total ADRs**	**960**	
**Age group**		
**0-18** years	89 (9)	
**19-65** years	773 (81)	0.0001
**>65** years	98 (10)	
**Sex**		
**Male**	618 (64)	0.0001
**Female**	342 (36)	
**Route of drug administration**		
**Oral**	987 (75)	0.0001
**Parenteral**	308 (23)	
**Topical**	21 (2)	

ADR=Adverse Drug Reaction, AMC=ADR Monitoring Centre, n=number

Figure 1 Frequency of type of ADRs reported per year at an AMC in Central India

For peer review only - http://bmjopen.bmj.com/site/about/guidelines.xhtml

Figure 2 summarises the number of ADRs associated with pharmacological drugs classes. Totally, 1316 drugs were implicated in causing the ADRs and antimicrobials were associated with the maximum number of the ADRs (29%) followed by the drugs of Antiretroviral therapy (ART, 19%) and Antitubercular therapy (ATT, 16%). The most common substances implicated for 88 ADRs were zidovudine followed by efavirenz (34 ADRs), ethambutol (44 ADRs) and ciprofloxacin (34 ADRs, Figure 3).

Figure 2 Occurrence of ADRs related to the pharmacological drug classes at an AMC in Central India

Figure 3 Pharmacological substances implicated in causing ADRs at an AMC in Central India

Table 2 presents the types of ADRs reported in various system organ classes (SOC). The ADRs of skin and subcutaneous tissue disorders (28%) were most common among all SOCs followed by gastrointestinal systems (18%). Table 3 shows the severity and causality assessment of reported ADRs. Four percent of all reported ADRs were severe and the rest were mild to moderate. Totally, 26 drugs were associated with 36 serious ADRs. Electrolyte imbalance, and reproductive and breast disorders caused seven ADR cases, while immune system disorder caused 19 ADR cases. The outcome was fatal in 12 ADR episodes, while 13 recovered with sequelae, 774 cases (55%) either recovered (521) or were recovering (253) at the time of ADR reporting and 90 were not having any sign of recovery till the ADR was reported.

Table 2 Frequency of ADR symptoms based on the involvement of organ system at an AMC in Central India

SOC (System Organ Class)	ADR symptoms n (%) 1980 (100)	Most common ADRs (n)
Skin and Subcutaneous Tissue Disorder	548 (28)	Rash (231), Generalized Pruritis (113), Fixed drug eruption (64), Burning sensation (19)
Gastrointestinal Disorders	365 (18)	Vomiting (91), Nausea (87), Abdominal discomfort (71), Diarrhoea (52)
Nervous System Disorder	219 (11)	Dizziness (61), Headache (50), Paraesthesia (17), Peripheral

For peer review only - http://bmjopen.bmj.com/site/about/guidelines.xhtml

		neuropathy (12)
General Disorders	193 (10)	Multidrug resistance (35), Shivering (33), Generalized oedema (21), Weakness generalized (18)
Psychiatric Disorder	129 (7)	Anxiety (33), Sedation (24), Drowsiness (17), Psychosis (9)
Musculoskeletal and Connective Tissue Disorders	82 (4)	Myalgia (29), Tremors (17), Joint pain (7)
Respiratory Disorder	64 (3%)	Cough (18), Breathing difficulty (16), Dyspnoea (14)
Blood and Lymphatic System Disorder	60 (3%)	Anaemia (47), Thrombocytopenia (6)
Cardiac Disorder	45 (2%)	Tachycardia (19), Chest pain (16), Bradycardia (5)
Hepatobiliary Disorder	41 (2%)	Hepatotoxicity (39)
Nutritional and Metabolic Disorder	40 (2%)	Loss of appetite (28), Pallor (3), Weight gain (3)
Ear Disorder	31 (2%)	Impaired hearing (14), Tinnitus (8), Vertigo (8)
Vascular Disorder	31(2%)	Hypotension (16), Epistaxis (4), Hypertension (3)
Eye Disorder	26(1%)	Impaired vision (16), Eye irritation (2), Lacrimation (2)
Injury, Poisoning and Procedural Complications	25(1%)	Injection site reaction (12), Extravasation (6)
Renal Disorder	25(1%)	Increased serum creatinine (6), Haematuria (4), Dysuria (3)
Endocrine Disorders	23(1%)	Gynaecomastia (8), Moon face (5), Hypoglycaemia (3)
Immune System Disorder	19 (1%)	Allergic dermatitis (8), Allergic reactions (4), Anaphylactic reaction (4)
Electrolyte Imbalance	7(0.35%)	Hypokalemia (2), Hypocalcaemia (1), Hyperuricaemia (2)
Reproductive and Breast Disorder	7(0.35%)	Vaginal haemorrhage (2), Breast tenderness (1)

ADR=Adverse Drug Reaction, AMC=ADR Monitoring Centre, n=number

Table 3 Assessment of Severity and Causality of the ADRs reported at an AMC in Central India

**Type of Severity (HARTWIG SCALE)[8]**	**ADR n (%)**
**Total**	960
Mild	564 (59) *
Moderate	360 (37.5)
Severe	36 (4)
**Causality Assessment (WHO Scale)[9]**	
Certain	93 (5)
Probable/Likely	846 (43)
Possible	951 (48)
Unlikely	22 (1)
**Inaccessible** / Unclassifiable	68 (3)

*statistically significant p value>0.0001; ADR=Adverse Drug Reaction, n=number of ADRs

Discussion:

There is a wide gap between the occurrence and reporting of ADRs, not only in India but also worldwide. The present study illustrates the ADRs reported at an AMC in Central India for the first time and present a list of the causative drugs for the burden of ADRs. The study highlighted antimicrobials as the most common causative drugs for a high burden of ADRs followed by

For peer review only - http://bmjopen.bmj.com/site/about/guidelines.xhtml

the ART drugs and the ATT drugs at the AMC. The occurrence of ADRs was more common in male patients (64%) and among patients of the breadwinner age group (19-65 years, 81%). Zidovudine was the agent that caused most ADRs (88) followed by ethambutol, ciprofloxacin and efavirenz.

Reporting of spontaneous ADRs is a collaborative study conducted with the help of inputs from healthcare providers or a person close to the patients. The maximum number of ADRs were reported in the year 2016 (224). The number of reported ADRs decreased after achieving a peak in the year 2016. The maximum number of serious ADRs were reported in 2017 and the non-serious ADRs in 2016. Although the data collection started in July 2013, yet the number of reported ADRs is lower for half a year. These low numbers might be due to the initial stage of the study when the collaborators were being sensitized for data collection. However, the reduction in the number of reported ADRs from 2016 onwards was a stimulus for us to identify the factors responsible for under-reporting and plan for the corrective measure to improve the reporting. In our study, significantly more ADRs were documented in the male patients than the female patients which are consistent with the earlier studies [14,15] whereas, few studies have also reported female preponderance [16,17]. However, the gender impact on the ADR occurrence cannot be explained here and might be an incidental finding and has no effect on the occurrence of reporting of the ADRs. In our study, the ADRs were predominantly reported in patients of age group between 19-65 years which is also similar to a study conducted by Daulat et al [14] and Thakare et al [17]. This is because it is a wide age group range, and this is likely the major population that attends hospital more frequently and receives drug therapy.

In our study, the pharmacological class of drugs implicated in causing ADRs shows that antimicrobials caused the maximum number of ADRs (29%). This might be because antimicrobials are one of the most prescribed drug classes specifically in LMICs [18,19]. It is also documented that this class is prescribed for unindicated conditions providing an opportunity to

For peer review only - http://bmjopen.bmj.com/site/about/guidelines.xhtml

develop antimicrobial resistance [15,20,21,22]. The other commonly prescribed pharmacological class of drugs implicated for ADRs in our study were ART, ATT, non-steroidal anti-inflammatory drugs (NSAIDs), CNS and CVS drugs in descending order. The ART and ATT drugs, despite their efficacy, are continuously found associated with a wide range of potential adverse effects [23]. The occurrence of ADRs related to ART and ATT drugs may result in poor compliance with the treatment that can lead to the development of resistance and treatment failure including virologic failures [24,25,26]. No common drug class could be implicated for causing serious ADRs events.

Polypharmacy is prevalent globally and the occurrence of ADR is directly proportional to the number of prescribed drugs thus, polypharmacy must be discouraged [27,28]. However, in the present study single drug was attributed for most of the ADRs (73%). It was observed that in most of the reports the drug that was most suspected of being associated with the specific ADR was reported and not the whole treatment. NSAIDs are often a constituent of many rational and irrational fixed-dose combinations and this could be a cause of the high incidence of the ADRs related to the NSAIDs [29]. This was reflected in our study where besides antimicrobials and ART drugs the antiepileptics and NSAIDs also caused substantial ADRs [19-23, 30-34]. In the present study, the most prescribed pharmacological substances among the patients having ADRs were zidovudine, ethambutol and ciprofloxacin while in other studies ceftriaxone, zidovudine, and phenytoin sodium were the most prescribed drugs [30-34]. Zidovudine and Ethambutol are part of multidrug ATT and ART regimes, respectively. Factors including the dose of the drugs, drug-drug interactions as well as genetic differences of the study population could also affect the number of Adverse drug reactions caused by drugs [35].

Most of the patients had mild ADRs in our study, as observed in other Indian studies [15,17]. ADRs of skin and subcutaneous tissue disorders (28%) were most common among all SOCs. Skin rashes were the most common complaints of followed by gastrointestinal complaints

For peer review only - http://bmjopen.bmj.com/site/about/guidelines.xhtml

(18%), nervous system disorders (11%), general disorders (10%) and psychiatric disorders (7%). Skin and subcutaneous tissues are among the most targeted organs for ADRs. The disorders are reported more frequently because these develop within one week of drug administration and can easily be spotted when the patients are still under the close supervision of the healthcare providers [15,36,37]. Timely recognition of skin ADRs is crucial for its classification, management, and causality assessment.

The causality assessment was conducted based on the WHO probability scale. According to the assessment, most cases could be categorized as 'Possible' (48) followed by 'Probable' (43). ADRs are rarely categorised as 'Certain' as it requires to re-challenge the patient with the same causative drug, which is not ethical, hence the assessment frequently goes to the Possible and Probable category [15,38]. Though 26 drugs were associated with serious, life-threatening ADRs, yet none of the drug classes could be implicated for serious ADRs. This was because of insufficient data due to under-reporting of ADRs, and the wide range of drugs prescribed. An adequate ADR reporting is required to preserve the clinical information that could prevent significant damage to patients, plan and implement measures to reduce their impact on public health and consequently minimize the burden on the health care system. Under-reporting a challenge in pharmacovigilance when other AMC contributed with 212 ADRs per month our AMC reported an average of 19 ADRs per month in 2016 while dropped to 11 in 2019 [39].

Early identification and management of ADRs will not only improve patient safety, quality of life but also reduce the cost of treatment. Labelling of new ADRs will help to prepare the prescribing policies and modify the prescribing patterns. Reduction in prescribing of the flagged drugs will reduce the prevalence of ADRs. We believe that disseminating results to the prescribers through feedback might reduce prescribing of causative drugs and improve reporting of ADRs. A knowledge, attitude and practice-based study conducted in Norway found that ADRs can be prevented by collecting reliable information about their frequencies

For peer review only - http://bmjopen.bmj.com/site/about/guidelines.xhtml

and possible risk factors [7]. Therefore, we propose to develop and implement strategies for the community and specifically HCWs to encourage reporting and monitoring of ADRs.

Strengths and limitations of this study

The strength of our study is that this is the first long term ADR data reported from an AMC in Central India. This study is used to implicate drugs most notoriously causing ADR and dissemination of the results of this study will help in the reduction of prescription of these drugs. The study is used to form a feedback plan to improve under-reporting. Our study has few limitations, the data was collected based on spontaneous reporting rather than active surveillance, which would have been a better method. Another limitation is that we could not estimate the precise incidence of suspected ADRs attributable to a particular drug. However, this was due to fewer reported cases of ADRs.

Conclusion

The maximum number of ADRs were reported from the breadwinner group (males and 19-65 years). Antimicrobials caused the maximum number of ADRs. This highlights an alarming situation in addition to the antimicrobial resistance. Prudent use of antimicrobials is once again proven as need of the hour. ADRs of skin and subcutaneous tissue disorders were most frequent among all SOCs. Most of the ADRs were mild and belonged to the 'Possible' group of probability scale. However, under-reporting of ADRs at our AMC is evident and a major factor for the pause in performing severity and causality assessment. Thus, the prevalence of ADRs reported in the present study can be perceived as the tip of an iceberg.

To deal with the problem of under-reporting we suggest continuous training programmes for HCPs, seeking the collaboration of nearby health care facilities, and most importantly giving

view only

feedback to the HCWs to impart confidence, awareness and knowledge about the ADR causing drugs and will encourage reporting ADR events.

Future implications

Our results indicate the need to develop and test a model of motivational educational- approach focusing on health care workers and community to increase ADR reporting in the settings associated with our AMC. Neither the global pharmacovigilance nor the PvPI has designed a plan, to analyse and to provide feedback on the ADR data to the prescribers. Providing reliable and balanced information, and a valid assessment of the risk-benefit profile of the marketed drugs to the healthcare professionals at a local level is essential. This knowledge might improve patients' safety while prescribing drugs, support patient care and public health programs in the settings. Therefore, we propose to develop a model with a strategy to provide feedback and knowledge to improve ADR reporting. Furthermore, providing feedback to the prescribers and sensitizing about the flagged drugs will be an encouragement for reporting ADRs. HCPs will be assured that reporting ADRs has no legal implications.

Modification of the organizational structure, training of HCPs and availability of resources can improve early detection of ADRs. We also suggest planning and start an ADR awareness and training campaign in collaboration with HCPs of the hospitals in the periphery, district, and private hospitals to motivate them to report ADRs. However, the long-term sustainability of these measures is most crucial as the effectiveness of interventions diminishes with time. The use of a digital platform such as SMS and E-mails might play an important role in reminding and keeping them motivated and sensitized to report ADRs timely.

Acknowledgement

For peer review only - http://bmjopen.bmj.com/site/about/guidelines.xhtml

The authors acknowledge the active support of the consultants and physicians in data collection. The Authors extend thanks to the IPC PvPI, Ghaziabad for encouraging and permitting the publication. Thanks are due to the management of the AMC-RDGMC for their support. The authors also acknowledge Mr Amit Pawar for his assistance during the study.

Author Contributions MS and RB conceptualized the study and analyses. ST collected the data. MS and RB analysed the data and drafted the manuscript. All authors contributed to and approved the present form of the manuscript.

Funding statement This research received no specific grant from any funding agency in the public, commercial, or not-for-profit sectors

Competing interest The authors declare no competing interests.

Data Sharing statement As per the protocol of the institutional ethics committee to maintain full confidentiality, the data can be made available through a request form addressing, The Chairman, Ethics Committee, R.D. Gardi Medical College, Agar Road, Ujjain, Madhya Pradesh, India 456006 (email: iecrdgmc@yahoo.in, uctharc@sancharnet.in), giving all details of the article. Quote the IEC approval number: 68/2019 along with the request.

Licence statement

The Corresponding Author has the right to grant on behalf of all authors and does grant on behalf of all authors, an exclusive licence on a worldwide basis to the BMJ Publishing Group Ltd (“BMJ”), and its Licensees to permit this article (if accepted) to be published in The BMJ’s editions and any other BMJ products and to exploit all subsidiary rights.

Research Checklist is not included in the cover letter because the study presents results of a survey, therefore as per protocol the checklist is not required.

References

For peer review only - http://bmjopen.bmj.com/site/about/guidelines.xhtml

1. Vivekanandan K, Thota P, Janarthanan VV, Singh GN. Pharmacovigilante's in the pharmacovigilance programme of India: Ideal qualities and skills. J Young Pharm 2016;8:291-2. (assessed 08 April 2021)
2. Nirojini PS, Yemineni R, Nadenla RR. Monitoring and Reporting of Adverse Drug Reactions in A South Indian Tertiary Care Hospital. Int J Pharm Sci Rev Res. 2014;24(1):259-62.
3. Datta S, Sengupta S. An evaluation of knowledge, attitude, and practice of adverse drug reaction reporting in a tertiary care teaching hospital of Sikkim. Perspect Clin Res 2015;6:200-6.
4. Mittal N, Mittal R, Gupta MC. An overview of the pharmacovigilance system in India. Clin Res Regul Aff 2016;33:4-8.
5. Tandon VR, Mahajan V, Khajuria V, Gillani Z. Under-reporting of adverse drug reactions: a challenge for pharmacovigilance in India. *Indian J Pharmacol*. 2015;47(1):65-71. doi:10.4103/0253-7613.150344 (assessed 08 April 2021)
6. Hazell L, Shakir SAW. Under-reporting of adverse drug reactions: A systematic review. Drug Saf. 2006; 29 (5) :385-96.366
7. Castberg I, Reimers A, Sandvik P, Aamo TO, Spiqset O. Adverse drug reactions of antidepressants and antipsychotics: Experience, knowledge and attitudes among Norwegian psychiatrists. Nord J Psychiatry. 2006;60:227–33.
8. Indian Pharmacopoeia Commission, Ministry of Health and family Welfare, Govt. of India. www.ipc.gov.in (assessed 08 April 2021)
9. ADR form PvPI. Available from: http://www.cdsco.nic.in/writereaddata/ADR%20form%20PvPI.pdf. (assessed 08 April 2021)
10. IA Eland, KJ Belton, AC van Grootheest, AP Meiners, MD Rawlins, and BH Ch Stricker. Attitudinal survey of voluntary reporting of adverse drug reactions British journal of

For peer review only - http://bmjopen.bmj.com/site/about/guidelines.xhtml

clinical Pharmacology1999 Oct; 48(4): 623–627.doi: 10.1046/j.1365-2125.1999.00060.x (assessed 08 April 2021)

11. Schumock GT, Seeger JD, Kong SX. Control charts to monitor rates of adverse drug reactions. Hosp Pharm 1995;30:1088, 1091‐2,1095‐6.
12. The use of the WHO–UMC system for standardised case causality assessment. Accessed from: http://www.WHO-UMC.org/graphics/4409.pdf (assessed 08 April 2021)
13. Hartwig SC, Siegel J, Schneider PJ. Preventability and severity assessment in reporting adverse drug reactions. Am J Hosp Pharm 1992;49:2229‐32.
14. Daulat MP, Abishake VJ, Singh P, Raj B. A prospective study of adverse drug reactions in a tertiary care teaching hospital. Int J Basic Clin Pharmacol 2018;7:1965-69.
15. Ramakrishnaiah H et al. Int J Basic Clin Pharmacol. 2015 Jun;4(3):515-521 International Journal of Basic & Clinical Pharmacology | May-June 2015 | Vol 4 | Issue 3 Page 521
16. Singh P, Agrawal M, Hishikar R, Joshi U, Maheshwari B, Halwai A. Adverse drug reactions at adverse drug reaction monitoring center in Raipur: Analysis of spontaneous reports during 1 year. *Indian J Pharmacol*. 2017;49(6):432-437. doi:10.4103/ijp.IJP_781_16 (assessed 08 April 2021)
17. Thakare VS, Kavitha V D, Langade D. Prospective observational study to evaluate adverse drug reactions pattern in a tertiary level teaching hospital. Natl J Physiol Pharm Pharmacol 2019;9(5):434-437.
18. Landstedt K, Stålsby-Lundborg C, Sharma A, Johansson F, Sharma M. Antibiotic prescriptions to the inpatients having non-bacterial diagnosis at medicine departments of two private sector hospitals in Madhya Pradesh, India: a cross sectional study. *BMJ Open* 2017;7:e012974. doi:10.1136/bmjopen-2016- 012974. Available at URL- http://bmjopen.bmj.com/content/bmjopen/7/4/e012974.full.pdf (assessed 08 April 2021)

For peer review only - http://bmjopen.bmj.com/site/about/guidelines.xhtml

19. Anna Makhowska, Jonatan Sparentofft, Cecilia Stålsby Lundborg, Megha Sharma. Perioperative antibiotic prescribing in surgery departments of two private sector hospitals in Madhya Pradesh, India. ***Perioperative Medicine.*** (2019) 8:10. DOI: https://doi.org/10.1186/s13741-019-0121-3 and https://doi.org/10.1186/s13741-019-0123-1 (assessed 08 April 2021)

20. Wester K, Jönsson AK, Spigset O, Druid H, Hägg S. Incidence of fatal adverse drug reactions: a population based study. Br J Clin Pharmacol. 2008;65:573-9.

21. Gor AP, Desai SV. Adverse drug reactions (adr) in the inpatients of medicine department of a rural tertiary care teaching hospital and influence of pharmacovigilance in reporting ADR. Indian J Pharmacol. 2008;40(1):37-40.

22. Vora MB, Trivedi HR, Shah BK, Tripathi CB. Adverse drug reactions in inpatients of internal medicine wards at a tertiary care hospital: a prospective cohort study. J Pharmacol Pharmacother. 2011;2(1):21-5.

23. Shet A, Antony J, Arumugam K, Kumar Dodderi S, Rodrigues R, DeCosta A (2014) Influence of Adverse Drug Reactions on Treatment Success: Prospective Cohort Analysis of HIV-Infected Individuals Initiating First-Line Antiretroviral Therapy in India. PLoS ONE 9(3): e91028. https://doi.org/10.1371/journal.pone.0091028 (assessed 08 April 2021)

24. Manju R, Maheshwari YN, Ramya JE, Krishnamoorthy K. Adverse drug reaction monitoring of antitubercular drugs during intensive phase at tertiary care medical college hospital: A prospective study. Natl J Physiol Pharm Pharmacol 2020;10(11):976-980.

25. Mukherjee S, Era N, Saha B, Tripathi SK. Adverse drug reaction monitoring in patients on antiretroviral therapy in a tertiary care hospital in Eastern India. *Indian J Pharmacol.* 2017;49(3):223-228. doi:10.4103/ijp.IJP_304_16 (assessed 08 April 2021)

For peer review only - http://bmjopen.bmj.com/site/about/guidelines.xhtml

26. Abah IO, Ncube NBQ, Bradley HA, AgbaJi OO, Kanki P. Antiretroviral Therapy-associated Adverse Drug Reactions and their Effects on Virologic Failure- A Retrospective Cohort Study in Nigeria. *Curr HIV Res*. 2018;16(6):436-446. doi:10.2174/1389450120666190214144609 (assessed 08 April 2021)

27. Nguyen JK, Fouts MM, Kotabe SE, Lo E. Polypharmacy as a risk factor for adverse drug reactions in geriatric nursing home residents. Am J Geriatr Pharmacother. 2006 Mar;4(1):36-41. doi: 10.1016/j.amjopharm.2006.03.002. PMID: 16730619. (assessed 08 April 2021)

28. 2. R., S., B. N. J., J. K. P., and S. L. V. POLYPHARMACY INDUCED DRUG INTERACTIONS, ADVERSE DRUG REACTIONS (ADR) AND MEDICATION ERRORS IN TERTIARY CARE SOUTH INDIAN HOSPITAL. International Journal of Pharmacy and Pharmaceutical Sciences, Vol. 11, no. 2, Feb. 2019, pp. 88-93, doi:10.22159/ijpps.2019v11i2.29508. (assessed 08 April 2021)

29. Gor AP, Saksena M. Adverse drug reactions of nonsteroidal anti-inflammatory drugs in orthopedic patients. *J Pharmacol Pharmacother*. 2011;2(1):26-29. doi:10.4103/0976-500X.77104 (assessed 08 April 2021)

30. Kathiria JM, Sattigere BM, Desai PM, Patel SP. A study of adverse drug reactions in patients admitted to intensive care unit of a tertiary care teaching rural hospital. Int J Pharm Pharm Sci. 2013;5(1):160-3.

31. Sharma SK. Zidovudine-induced anaemia in HIV/AIDS. Indian J Med Res. 2010;132:359-61.

32. Shah SP, Desai MK, Dikshit RK. Analysis of cutaneous adverse drug reactions at a tertiary care hospital: a prospective study. Trop J Pharm Res. 2011;10(4):517-22.

For peer review only - http://bmjopen.bmj.com/site/about/guidelines.xhtml

33. Vijendra R, Pundarikaksha HP, Gopal MG, Girish K, Vasundara K, Jyothi R. A prospective study of cutaneous adverse drug reaction in a tertiary care hospital. Natl J Basic Med Sci. 2013;3(1):44-51.

34. Hussain MM, Girhepunje K, Pal R, Siddiqua SS. Incidence of adverse drug reactions in a tertiary care hospital: a systematic review and meta-analysis of prospective studies. Pharm Lettr. 2010;2(3):358-68.

35. Hema NG, Bhuvana KB, Virupaksha HM. Critical Assessment of Adverse drug Reactions to Antitubercular Drugs in a Government Teaching Hospital. IJBMS, 2013; 4(2):60-67.

36. Arulmani R, Rajendran SD, Suresh B. Adverse drug reaction monitoring in a secondary care hospital in South India. Br J Clin Pharmacol. 2008;65(2):210-6.

37. Modi A, Desai M, Shah S, Shah B. Analysis of Cutaneous Adverse Drug Reactions Reported at the Regional ADR Monitoring Center. *Indian J Dermatol*. 2019;64(3):250. doi:10.4103/ijd.IJD_682_16 https://www.ncbi.nlm.nih.gov/pmc/articles/PMC6537693/ (assessed 08 April 2021)

38. Laskar JI, Chakravarty P, Dewan B. A study on incidence of adverse drug reactions with commonly prescribed drugs and causality assessment in Silchar Medical College and Hospital. Int J Basic Clin Pharmacol 2017;6:1175-83.

39. Tandon VR, Mahajan V, Khajuria V, Gillani Z. Under-reporting of adverse drug reactions: a challenge for pharmacovigilance in India. *Indian J Pharmacol*. 2015;47(1):65-71. doi:10.4103/0253-7613.150344

For peer review only - http://bmjopen.bmj.com/site/about/guidelines.xhtml

Figure 1 Frequency of type of ADRs reported per year at an AMC in Central India

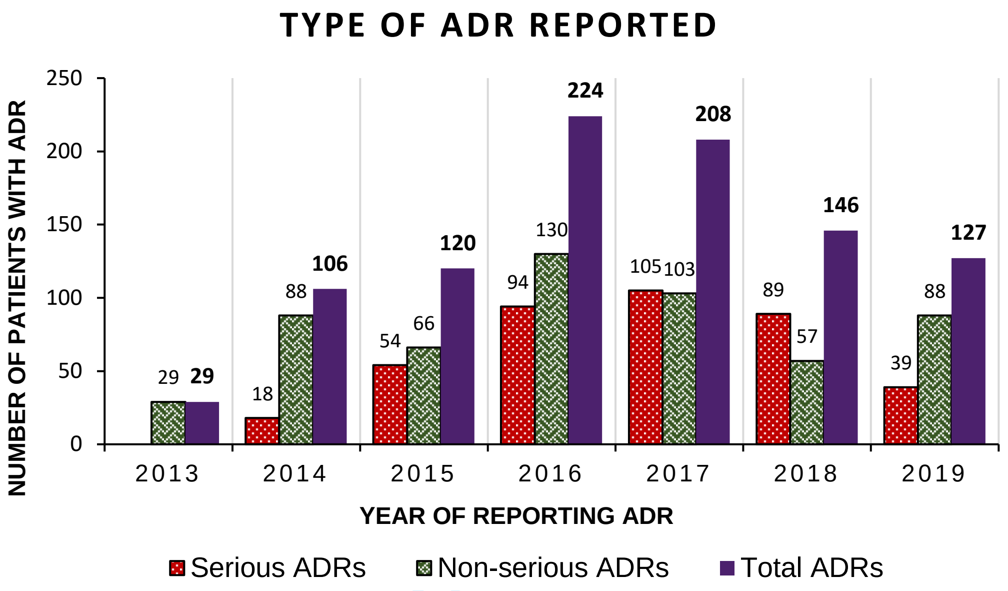

ADR=Adverse Drug Reaction, AMC=ADR Monitoring Centre

Figure 2 Occurrence of ADRs related to the pharmacological drug classes at an AMC in Central India

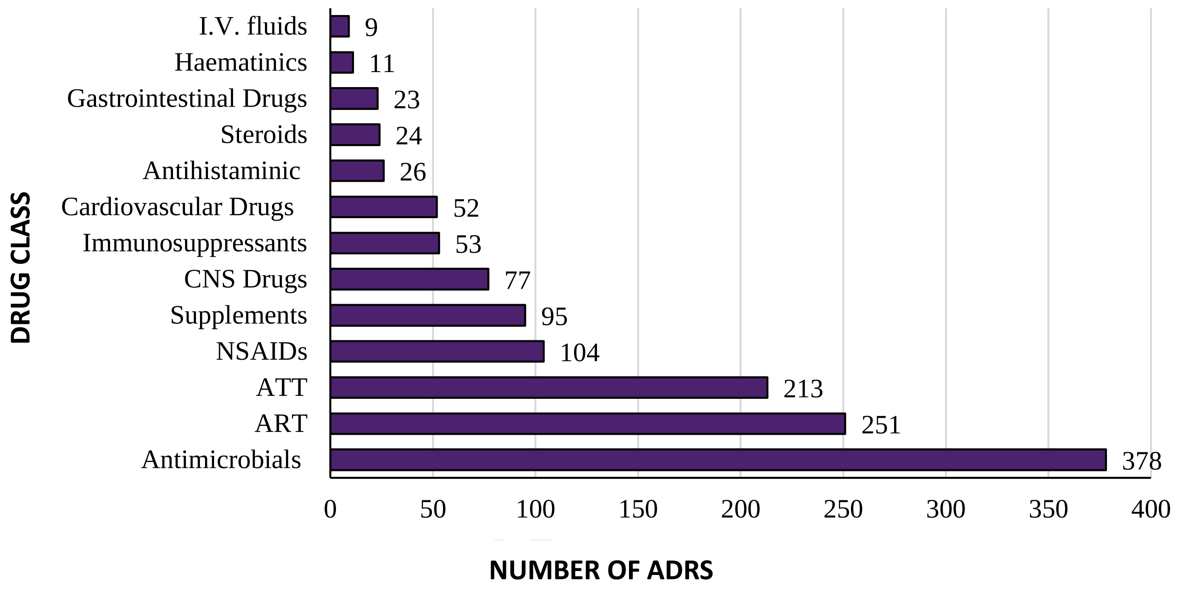

ADR=Adverse Drug Reaction, AMC=ADR Monitoring Centre ART=Antiretroviral therapy, ATT=Antitubercular therapy, CNS=Central Nervous System, NSAID= Non-steroidal anti-inflammatory drugs, I.V. fluids= Intravenous fluid

For peer review only

Figure 3: Pharmacological substances implicated in causing ADRs at an AMC in Central India

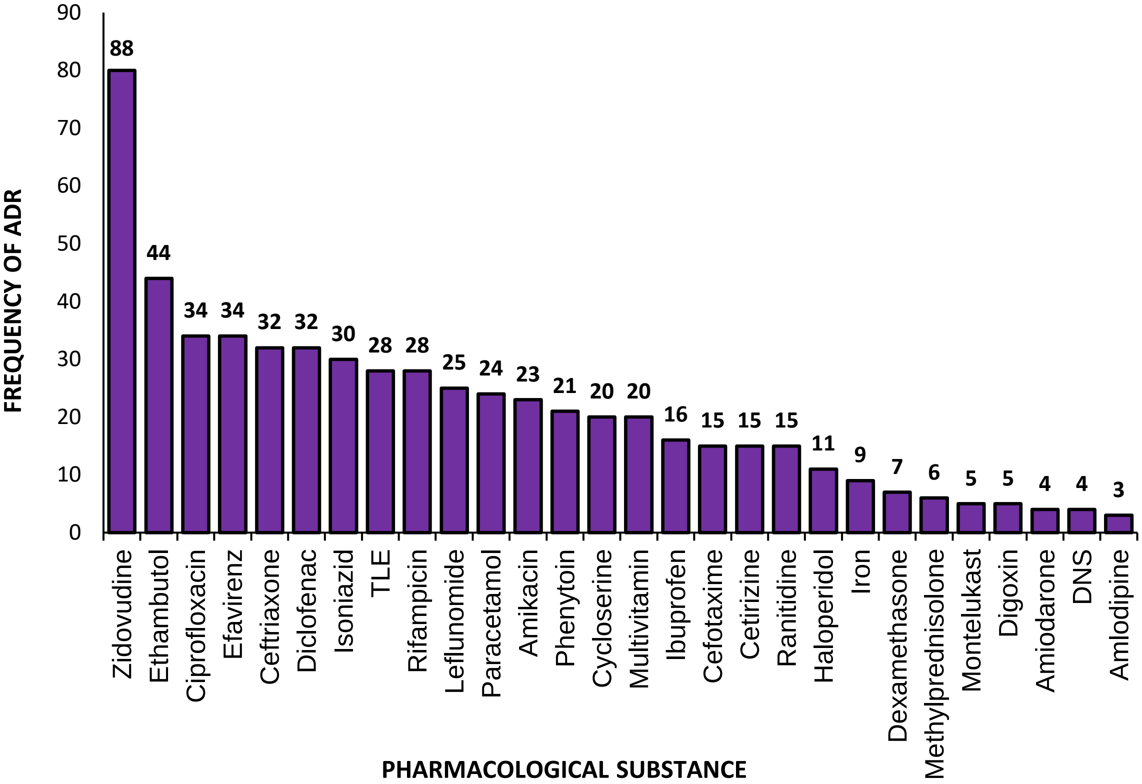

ADR=Adverse Drug Reaction, AMC=ADR Monitoring Centre, DNS=Dextrose Normal Saline, T/L/E=Tenofovir/Lamivudine/Efaviren

For peer review only - http://bmjopen.bmj.com/site/about/guidelines.xhtml

ANNEXURE – I

Version-1.3

SUSPECTED ADVERSE DRUG REACTION REPORTING FORM

For VOLUNTARY reporting of Adverse Drug Reaction by Healthcare Professionals

INDIAN PHARMACOPOEIA COMMISSION(National Coordination Centre-Pharmacovigilance Programme of India)
Ministry of Health & Family Welfare, Government of India Sector-23, Raj Nagar, Ghaziabad-201002

A. PATIENT INFORMATION

1. Patient Initials ________	2. Age at the time of Event or Date of Birth ________	3. M ☐ F ☐ Other ☐ / 4. Weight________Kgs

Reg. No. /IPD No. /OPD No. /CR No. :
AMC Report No. :
Worldwide Unique No. :

B. SUSPECTED ADVERSE REACTION

5. Event/Reaction start date (dd/mm/yyyy)
6. Event/Reaction stop date (dd/mm/yyyy)
6 (A). Onset Lag Time
7. Describe Event/Reaction with treatment details, if any

12. Relevant tests/ laboratory data with dates

13. Relevant medical/medication history (e.g. allergies, race, pregnancy, smoking, alcohol use, hepatic/renal dysfunction, past surgery etc.)

14. Seriousness of the reaction: No ☐ if Yes ☐(please tick anyone)
☐ Death (dd/mm/yyyy) ☐ Congenital-anomaly
☐ Life threatening ☐ Disability
☐Hospitalization/Prolonged ☐ Other Medically important

15. Outcomes
☐ Recovered ☐ Recovering ☐ Not recovered
☐ Fatal ☐ Recovered with sequelae ☐ Unknown

C. SUSPECTED MEDICATION(S)

S.No	8. Name (Brand/Generic)	Manufacturer (if known)	Batch No. / Lot No.	Exp. Date (if known)	Dose used	Route used	Frequency (OD, BD etc.)	Therapy dates: Date started	Therapy dates: Date stopped	Indication	Causality Assessment
i											
ii											
iii											
iv*											

S.No as per C	9. Action Taken (please tick): Drug withdrawn	Dose increased	Dose reduced	Dose not changed	Not applicable	Unknown	10. Reaction reappeared after reintroduction (please tick): Yes	No	Effect unknown	Dose (if reintroduced)
i										
ii										
iii										
iv										

11. Concomitant medical product including self-medication and herbal remedies with therapy dates (Exclude those used to treat reaction)

S.No	Name (Brand/Generic)	Dose used	Route used	Frequency (OD, BD, etc.)	Therapy dates: Date started	Therapy dates: Date stopped	Indication
i							
ii							
iii*							

Additional Information:

D. REPORTER DETAILS

16. Name and Professional Address:________________________
____
Pin:____________E-mail________________________________
Tel. No. (with STD code)________________________________
Occupation:__________________ Signature:________________

17. Date of this report (dd/mm/yyyy):

Sig. and Name of Receiver-

Confidentiality: The patient's identity is held in strict confidence and protected to the fullest extent. Submission of a report does not constitute an admission that medical personnel or manufacturer or the product caused or contributed to the reaction. Submission of an ADR report does not have any legal implication on the reporter.

*use separate page for more information

For peer review only - http://bmjopen.bmj.com/site/about/guidelines.xhtml

BMJ Open

Surveillance of adverse drug reactions at an adverse drug reaction monitoring centre in Central India: A seven-year surveillance study

Journal:	*BMJ Open*
Manuscript ID	bmjopen-2021-052737.R1
Article Type:	Original research
Date Submitted by the Author:	24-Aug-2021
Complete List of Authors:	Sharma, Megha; Karolinska Institutet, Department of Global Public Health; RD Gardi Medical College, Department of Pharmacology<br>Baghel, Ruchi; RD Gardi Medical College, Department of Pharmacology<br>Thakur, Sunil; RD Gardi Medical College, Department of Pharmacology<br>Adwal, Sandeep; Zydus Medical College and Hospital, Department of Pharmacology
**Primary Subject Heading**:	Pharmacology and therapeutics
Secondary Subject Heading:	Pharmacology and therapeutics, Public health, Medical education and training, Infectious diseases, Global health
Keywords:	CLINICAL PHARMACOLOGY, Health & safety < HEALTH SERVICES ADMINISTRATION & MANAGEMENT, Risk management < HEALTH SERVICES ADMINISTRATION & MANAGEMENT, Adverse events < THERAPEUTICS, Toxicity < THERAPEUTICS, Pharmacology < TROPICAL MEDICINE

SCHOLARONE™
Manuscripts

I, the Submitting Author has the right to grant and does grant on behalf of all authors of the Work (as defined in the below author licence), an exclusive licence and/or a non-exclusive licence for contributions from authors who are: i) UK Crown employees; ii) where BMJ has agreed a CC-BY licence shall apply, and/or iii) in accordance with the terms applicable for US Federal Government officers or employees acting as part of their official duties; on a worldwide, perpetual, irrevocable, royalty-free basis to BMJ Publishing Group Ltd ("BMJ") its licensees and where the relevant Journal is co-owned by BMJ to the co-owners of the Journal, to publish the Work in this journal and any other BMJ products and to exploit all rights, as set out in our licence.

The Submitting Author accepts and understands that any supply made under these terms is made by BMJ to the Submitting Author unless you are acting as an employee on behalf of your employer or a postgraduate student of an affiliated institution which is paying any applicable article publishing charge ("APC") for Open Access articles. Where the Submitting Author wishes to make the Work available on an Open Access basis (and intends to pay the relevant APC), the terms of reuse of such Open Access shall be governed by a Creative Commons licence – details of these licences and which Creative Commons licence will apply to this Work are set out in our licence referred to above.

Other than as permitted in any relevant BMJ Author's Self Archiving Policies, I confirm this Work has not been accepted for publication elsewhere, is not being considered for publication elsewhere and does not duplicate material already published. I confirm all authors consent to publication of this Work and authorise the granting of this licence.

For peer review only

Surveillance of adverse drug reactions at an adverse drug reaction monitoring centre in Central India: A seven-year surveillance study

Authors: Megha Sharma[#*1,2], Ruchi Baghel*[1], Sunil Thakur[1], Sandeep Adwal[3]

[1]Department of Global Public Health- Health Systems and Policy: Medicines, Karolinska Institutet, Stockholm, Sweden. 171 77

[2]Department of Pharmacology, R. D. Gardi Medical College, Ujjain, Madhya Pradesh, India. 456006

[3]Department of Pharmacology, Zydus Medical College and Hospital, Nimnaliya, Dahod, Gujarat. 389151

*Contributed equally

[#] Corresponding author

Email:

Megha Sharma: megha.sharma@ki.se

Ruchi Baghel: drruchibaghel@gmail.com

Sunil Thakur: sunilcsthakur@gmail.com

Sandeep Adwal: adwalsandeep@yahoo.co.in

Corresponding author

[#] Associate Professor, Department of Pharmacology

R. D. Gardi Medical College Ujjain, (MP), India

ZIP/PIN 456006

Keywords: Pharmacovigilance, Adverse drug reactions, ADR Monitoring Centre, spontaneous ADRs, Antimicrobials, Antiretroviral therapy, Antitubercular therapy, India

For peer review only - http://bmjopen.bmj.com/site/about/guidelines.xhtml

For peer review only

Abstract

Objectives

To analyse and present the occurrence and severity of spontaneous ADRs reports prospectively registered at an AMC in Central India.

Setting and data

The survey was conducted between 2013 and 2019 at an ADR Monitoring Centre in Central India. ADRs were recorded using the standard 'Suspected ADR Reporting form'.

Outcome measures: The causality of the ADRs were categorized using the WHO causality assessment scale to assess the relationship between a drug and the occurrence of an ADR.

Results

Totally 1980 spontaneous ADRs were reported involving 960 patients and 1316 drugs prescriptions. The occurrence of ADRs was common among male patients (64%) and patients of age between 19-65 years (81%). Antimicrobials caused 29% ADRs, followed by drugs of Antiretroviral therapy (19%). Zidovudine caused most ADRs (88%) followed by ethambutol and ciprofloxacin. The ADRs of skin and subcutaneous tissue disorders (28%) were most common among all system-organ classes followed by gastrointestinal systems (18%). Four percent of all reported ADRs were severe. A peak of ADR reports was attained in 2016 with 224 reports which decreased to 127 in 2019.

Conclusion

A high number of ADRs caused by antimicrobials is an alarming situation, which adds up to antimicrobial resistance. Judicious use of antimicrobials is yet again proven as need of the hour. Under-reporting of ADRs is evident in our study and is a major factor for the delay in the withdrawal of drugs responsible for causing ADRs. Interventions in terms of training and feedback are suggested to encourage and improve ADR reporting.

For peer review only

Article summary

Strengths and limitations of this study

- This is the first long-term study that presents the ADRs reported from Central India.
- The spontaneous self- and voluntary-reporting study design used is easy and low resource-demanding.
- The data was collected based on spontaneous reporting rather than active surveillance, which would have been a better method to overcome the issue of under-reporting.
- The results can be used to provide feedback and training for awareness and to improve the under-reporting of ADRs.
- There was no option to follow the patient or patient's record to confirm the final outcome.

For peer review only

For peer review only - http://bmjopen.bmj.com/site/about/guidelines.xhtml

Background

Pharmacovigilance is a process of post-marketing surveillance of drugs–that continues throughout the drug lifecycle. It is essential in analysing and managing the risks associated with drugs that are available for the use of the general population [1]. The results of pharmacovigilance certify the effectiveness and safety of drugs in terms of adverse drug reactions (ADRs). The occurrence of ADRs can prolong hospitalization and thereby increase the cost of hospital stay and treatment and initiate the requirement of additional clinical investigations in severe cases. World health organization (WHO) defines 'Pharmacovigilance' as the science and activities relating to the detection, assessment, understanding, and prevention of adverse effects or any other possible drug-related problems, including herbal medicines [2]. The WHO Collaborating Centre (WHOCC) for International Drug Monitoring at Uppsala Monitoring Centre (UMC), Sweden; promotes pharmacovigilance at the national level. The national data from the participating countries are shared with the UMC and compiled to generate a global adverse drug reaction (ADR) database. The ADR database is collected from various countries under the WHOCC ADR monitoring program and compiled together to generate signals or flags. A signal or flag is a presumed risk associated with the use of a drug and is supported by reliable data sources. Based on the signals or flags, recommendations are made for regulatory interventions at the international level in the form of labelling revisions or banning the drug and communicating the evident risks to the policymakers. India is one of the global partners in the global program and participates under the Ministry of family welfare via the Pharmacovigilance Programme of India (PvPI).

The PvPI is a WHO initiative to scrutinize drug-induced mortality and morbidity in India. The PvPI is a collaborative project between WHO and Central Drugs Standard Control Organization (CDSCO), Ministry of Health and Family Welfare in India to prevent the ADRs. Collection, reporting and follow-up of the adverse drug reactions (ADRs) occurring among the

For peer review only - http://bmjopen.bmj.com/site/about/guidelines.xhtml

patients are prime activities included under PvPI. ADR Monitoring Centres (AMCs) are basic units of the PvPI, and it works at the national level intending to identify, analyse, characterize, and estimate the extent of the problem associated with drug use. The program aims to improve the vigilance on drugs, enhance patient safety and achieve better health benefits [3,4]. Reporting ADRs at an institutional level provide valuable information about potential problems during drug usage in healthcare settings. However, the reports of signals or flags generated at UMC are not publicized to the AMCs. Moreover, there is a wide gap between the occurrence and reporting of ADRs globally [5,6]. A study conducted in Norway concludes that ADRs can be prevented by collecting reliable information about their frequencies and possible risk factors. [7] Thus, it is of utmost importance to collect and analyse the prevalence of ADR to; a) identify the potential risks with drugs, b) support management of diseases and, c) rationalise the prescribing practices.

Objectives

The study was conducted to analyse and present the occurrence and severity of spontaneous ADRs reports registered at an AMC in Central India.

Methods

Study design and setting

This non-interventional survey was a part of PvPI and presents the data collected from one of the AMC under PvPI. All ADR reports received from the hospitals and healthcare facilities between July 2013 and December 2019, at the AMC- R. D. Gardi Medical College, Ujjain in Central India were included in the study. The Medical College, established in the year 2000, is the first private medical college in the Madhya Pradesh state of India. The institute is recognised as an AMC since 2012 [8].

Data collection considerations and analysis

For peer review only - http://bmjopen.bmj.com/site/about/guidelines.xhtml

The Suspected ADR Reporting form, recommended by the CDSCO was used as the data collection tool (Annexure I) [9,10]. The suspected ADRs were spontaneously reported by the Health Care Professionals (HCPs), patients or patient's relatives; to our AMC either directly or through the Patient Safety Pharmacovigilance Associate (ST). All ADRs reported between 2013 and 2019 were included in the study.

The data was recorded and computerised in Microsoft Excel, using the protocols and formats provided by the National Coordination Centre of PvPI at the AMC. The data were analysed anonymously, at the group level. The analyses related to the patients' demography, frequency, and route of administration of ADR causative drugs, organ system involved and identification of the unlabelled ADRs were done using the established tools [11]. Appropriate descriptive statistics were used to analyse the quantitative data.

The causality of the reported adverse reactions was categorized into certain, probable, possible, un-assessable/unclassifiable, unlikely, conditional/unclassified using the WHO causality assessment scale [12]. This scale is used for the assessment of the relationship between a drug and the occurrence of an ADR. A serious reaction was characterized as a fatal, life threatening reaction that can prolong hospitalization, and cause a significant persistent disability, might result in a congenital anomaly, and require an intervention to prevent permanent damage or death. ADRs are categorised by Hartwig SC et. al. in seven levels based on their severity. Levels 1 and 2 falls under the mild category whereas levels 3 and 4 are under the moderate category and levels 5, 6 and 7 fall under the severe category [13]. The causal relationship between the suspected drug and the reaction was established using WHO–UMC standardized case causality assessment criteria [12].

Statistical methods

For peer review only - http://bmjopen.bmj.com/site/about/guidelines.xhtml

The data were analysed with Excel, and STATA V.13.1 (Stata Corp, College Station, Texas, USA). The frequencies and percentage of categorical values were calculated. Sum, median, mean, range and SD were calculated for the continuous numerical values. percentages were rounded to the closest whole number. The independent t-test was used for the comparison of normally distributed and continuous variables. The Chi^2 test was used for comparison of categorical values and p values <0.001 were considered as significant.

Patient and Public Involvement statement

Patients or the public (the patient or family members or a healthcare worker who noticed the ADR) were involved to report the ADRs in our research. The ADRs were recorded with the consent of the patient under Patient and Public Involvement. The person reporting an ADR could directly contact the pharmacovigilance team member and give the details of the ADR. The protocol of the collaborative project (WHO Collaborating Centre for International Drug Monitoring at Uppsala Monitoring Centre, Sweden) was followed.

Ethics statement

The study was approved by the Institutional Ethics Committee (IEC) of Ruxmaniben Deepchand Gardi Medical College, Ujjain, with approval number IEC Ref. No -68/2019. The personal identification variables were masked before analyses and analyses were conducted in groups to maintain full confidentiality. Since the results do not contain any personal information, taking consent from the patients was exempted. This was a non-interventional study where no influence was imposed on the treatment or medical care. Treating consultants were not approached regarding the reported ADRs as this was not the aim of the study. Full confidentiality was maintained throughout the analyses and the analyses were performed in groups to affirm patients' confidentiality. Necessary communication was accomplished with PvPI for permission to use the data for publication.

For peer review only - http://bmjopen.bmj.com/site/about/guidelines.xhtml

Results

Spontaneous ADR reports were collected and analysed during seven years at the AMC. A total of 1980 ADR reports were generated from 960 patients and were associated with 1316 prescriptions of drugs. The occurrence of ADRs dominated among males 64% (618) than female patients (Table 1). The maximum number of ADRs were reported were from the breadwinner group patients ageing between 19 and 65 years (81%), and the mean age of the patients was 38.02848 ± 0.5691212. A peak of ADR reports was attained in 2016 with 224 reports which decreased to 127 in 2019. The highest number of ADRs were reported in the year 2016 (224) while the maximum number of serious ADRs were reported in the year 2017 (105, Figure 1) and in the year 2018, the reports of serious ADRs exceeded the non-serious ADRs.

Table.1 Demographic parameter and route of drug administration among the patients developing ADRs at an AMC in Central India

Parameters		Number of Patients with ADRs, n (%)	P-value
**Total ADRs**		**960**	
**Age group**			
	**0-18** years	89 (9)	
	**19-65** years	773 (81)	0.0001
	**>65** years	98 (10)	
**Sex**			
	**Male**	618 (64)	0.0001
	**Female**	342 (36)	
**Route of drug administration**			
	**Oral**	987 (75)	0.0001
	**Parenteral**	308 (23)	
	**Topical**	21 (2)	

ADR=Adverse Drug Reaction, AMC=ADR Monitoring Centre, n=number

Figure 1 Frequency of type of ADRs reported per year at an AMC in Central India

Figure 2 summarises the number of ADRs associated with pharmacological drugs classes. In total, 1316 drugs were implicated in causing the ADRs and antimicrobials were associated with

For peer review only - http://bmjopen.bmj.com/site/about/guidelines.xhtml

the maximum number of the ADRs (29%) followed by the drugs of Antiretroviral therapy (ART, 19%) and Antitubercular therapy (ATT, 16%). The most common substances implicated for 88 ADRs were zidovudine followed by efavirenz (34 ADRs), ethambutol (44 ADRs) and ciprofloxacin (34 ADRs, Figure 3).

Figure 2 Occurrence of ADRs related to the pharmacological drug classes at an AMC in Central India

Figure 3 Pharmacological substances implicated in causing ADRs at an AMC in Central India

Table 2 presents the types of ADRs reported in various system organ classes (SOC). The ADRs of skin and subcutaneous tissue disorders (28%) were most common among all SOCs followed by gastrointestinal systems (18%).

Table 2 Frequency of ADR symptoms based on the involvement of organ system at an AMC in Central India

SOC (System Organ Class)	ADR symptoms n (%) 1980 (100)	Most common ADRs (n)
Skin and Subcutaneous Tissue Disorder	548 (28)	Rash (231), Generalized Pruritis (113), Fixed drug eruption (64), Burning sensation (19)
Gastrointestinal Disorders	365 (18)	Vomiting (91), Nausea (87), Abdominal discomfort (71), Diarrhoea (52)
Nervous System Disorder	219 (11)	Dizziness (61), Headache (50), Paraesthesia (17), Peripheral neuropathy (12)
General Disorders	193 (10)	Multidrug resistance (35), Shivering (33), Generalized oedema (21), Weakness generalized (18)
Psychiatric Disorder	129 (7)	Anxiety (33), Sedation (24), Drowsiness (17), Psychosis (9)
Musculoskeletal and Connective Tissue Disorders	82 (4)	Myalgia (29), Tremors (17), Joint pain (7)
Respiratory Disorder	64 (3%)	Cough (18), Breathing difficulty (16), Dyspnoea (14)
Blood and Lymphatic System Disorder	60 (3%)	Anaemia (47), Thrombocytopenia (6)
Cardiac Disorder	45 (2%)	Tachycardia (19), Chest pain (16), Bradycardia (5)
Hepatobiliary Disorder	41 (2%)	Hepatotoxicity (39)
Nutritional and Metabolic Disorder	40 (2%)	Loss of appetite (28), Pallor (3), Weight gain (3)
Ear Disorder	31 (2%)	Impaired hearing (14), Tinnitus (8), Vertigo (8)
Vascular Disorder	31(2%)	Hypotension (16), Epistaxis (4), Hypertension (3)

For peer review only - http://bmjopen.bmj.com/site/about/guidelines.xhtml

For peer review only

Eye Disorder	26(1%)	Impaired vision (16), Eye irritation (2), Lacrimation (2)
Injury, Poisoning and Procedural Complications	25(1%)	Injection site reaction (12), Extravasation (6)
Renal Disorder	25(1%)	Increased serum creatinine (6), Haematuria (4), Dysuria (3)
Endocrine Disorders	23(1%)	Gynaecomastia (8), Moon face (5), Hypoglycaemia (3)
Immune System Disorder	19 (1%)	Allergic dermatitis (8), Allergic reactions (4), Anaphylactic reaction (4)
Electrolyte Imbalance	7(0.35%)	Hypokalemia (2), Hypocalcaemia (1), Hyperuricaemia (2)
Reproductive and Breast Disorder	7(0.35%)	Vaginal haemorrhage (2), Breast tenderness (1)

ADR=Adverse Drug Reaction, AMC=ADR Monitoring Centre, n=number

Table 3 shows the list of drugs that were most frequently associated with the ADR symptoms. Leflunomide followed by gliclazide, and paracetamol caused rashes. A fixed-dose combination of antibiotics, cefoperazone with sulbactam was most commonly associated with generalized pruritis. Zidovudine caused anaemia and the combination of tenofovir, lamivudine and efavirenz caused gynaecomastia. Antibiotics were tagged as suspected drugs for the ADRs in 204 reports. The most common indications for prescribing antibiotics among the 204 cases were multidrug-resistant tuberculosis (34 ADRs), bacterial infections (20 ADRs), and surgical prophylaxis (18 ADRs). Most patients were resistant to the fixed-dose combination of antibiotics i.e., ceftriaxone and sulbactam. In a majority of the cases (99%) antibiotics were prescribed for an appropriate indication i.e., an infection.

Table 3 Most frequently associated drugs with ADR symptoms at an AMC in Central India

ADR Symptom (n)	Name of the drug, suspected for the ADR symptom (n)
Rash (231)	Leflunomide (40), Gliclazide (21), Paracetamol (20)
Generalized pruritis (113)	Cefoperazone with sulbactam (12), Cefotaxime (8), Levofloxacin (8), Diclofenac (7)
Vomiting (91)	Tenofovir (22), Quinine (19)
Nausea (87)	Zidovudine (23), TLE (18), Quinine (11)
Abdominal pain (71)	Zidovudine (9), Aspirin (8), Leflunomide (4)
Dizziness (61)	TLE (21), Ranitidine (7), Quinine (6)
Anaemia (47)	Zidovudine (43)
Hepatotoxicity (39)	ATT (21)
Multi Drug Resistance (35)	Ceftriaxone with tazobactam (15), Amikacin (9)
Anxiety (33)	TLE (12), Quinine (7)
Myalgia (29)	Zidovudine (173)
Injection site reaction (12)	Artesunate (3), Cefotaxime (3), Ceftriaxone (2)
Gynaecomastia (8)	TLE (7)

For peer review only

Increased serum creatinine (6)	Tenofovir (5)

n=number of reports, TLE=A combination of tenofovir, lamivudine and efavirenz, ATT= Antitubercular treatment

Table 4 shows the severity and causality assessment of reported ADRs. Four percent of all reported ADRs were severe and the rest were mild to moderate. Overall, 26 drugs were associated with 36 serious ADRs. Electrolyte imbalance, and reproductive and breast disorders caused seven ADR cases, while immune system disorder caused 19 ADR cases. The outcome was fatal in 12 ADR episodes, while 13 recovered with sequelae, 774 cases (55%) either recovered (521) or were recovering (253) at the time of ADR reporting and 90 were not having any sign of recovery till the ADR was reported.

Table 4 Assessment of severity and causality of the ADRs reported at an AMC in Central India

**Type of Severity (HARTWIG SCALE)[8]**	**ADR n (%)**
**Total**	960
Mild	564 (59) *
Moderate	360 (37.5)
Severe	36 (4)
**Causality Assessment (WHO Scale)[9]**	
Certain	93 (5)
Probable/Likely	846 (43)
Possible	951 (48)
Unlikely	22 (1)
**Inaccessible** / Unclassifiable	68 (3)

*Statistically significant p value>0.0001; ADR=Adverse Drug Reaction, n=number of ADRs

Discussion

There is a wide gap between the occurrence and reporting of ADRs, not only in India but also worldwide. The present study illustrates the ADRs reported at an AMC in Central India for the first time and present a list of the causative drugs for the burden of ADRs. The study highlighted antimicrobials as the most common causative drugs for a high burden of ADRs followed by the ART drugs and the ATT drugs at the AMC. The occurrence of ADRs was more common in male patients (64%) and among patients of the breadwinner age group (19-65 years, 81%).

For peer review only

For peer review only - http://bmjopen.bmj.com/site/about/guidelines.xhtml

Zidovudine was the agent that caused most ADRs (88) followed by ethambutol, ciprofloxacin and efavirenz.

Reporting of spontaneous ADRs is a collaborative study conducted with the help of inputs from healthcare providers or a person close to the patients. The maximum number of ADRs were reported in the year 2016 (224). The number of reported ADRs decreased after achieving a peak in the year 2016. The maximum number of serious ADRs were reported in 2017 and the non-serious ADRs in 2016. Although the data collection started in July 2013, yet the number of reported ADRs is lower for half a year. These low numbers might be due to the initial stage of the study when the collaborators were being sensitized for data collection. However, the reduction in the number of reported ADRs from 2016 onwards must act as a stimulus to plan future studies to identify the factors responsible for under-reporting including qualitative method of data collection. Extensive efforts are needed for the corrective measures to improve the reporting. In our study, significantly more ADRs were documented in the male patients than the female patients which are consistent with the earlier studies [14,15] whereas, few studies have also reported female preponderance [16,17]. However, the gender impact on the ADR occurrence cannot be explained here and might be an incidental finding and has no effect on the occurrence of reporting of the ADRs. In our study, the ADRs were predominantly reported in patients of age group between 19-65 years which is also similar to a study conducted by Daulat et al [14] and Thakare et al [17]. This is because it is a wide age group range, and this is likely the major population that attends hospital more frequently and receives drug therapy.

The results based on pharmacovigilance data are crucial to generate signals and alerts. However, signals are generated based on a substantial number of reports after following the protocol. Yet, the frequencies of ADRs reported by some of the drugs in the present study are alarming. The pharmacological drug classes implicated for causing ADRs shows that antimicrobials caused the maximum number of ADRs (29%). This might be because

For peer review only - http://bmjopen.bmj.com/site/about/guidelines.xhtml

antimicrobials are one of the most prescribed drug classes specifically in LMICs [18,19]. It is also documented that this class is prescribed for unindicated conditions providing an opportunity to develop antimicrobial resistance [15,20-22]. The ADR reports associated with antibiotics can facilitate the development of appropriate policies and guidelines for the judicious use of antibiotics and thereby combat antimicrobial resistance [23]. A fixed-dose combination of cefoperazone with sulbactam was most commonly associated with generalized pruritis. Among 35 cases of multi-drug resistance, most cases were encountered with a fixed-dose combination of ceftriaxone and sulbactam followed by Amikacin. In our study, the antibiotics were prescribed for an infectious indication in almost all reported cases.

Besides antibiotics, the other commonly prescribed pharmacological drug classes implicated for ADRs were ART, ATT, non-steroidal anti-inflammatory drugs (NSAIDs), CNS and CVS drugs in descending order of causing ADRs. The ART and ATT drugs, despite their efficacy, are continuously found associated with a wide range of potential adverse effects [24]. The occurrence of ADRs related to ART and ATT drugs may result in poor compliance with the treatment that can lead to the development of resistance and treatment failure including virologic failures [25-27]. No common drug class could be implicated for causing serious ADRs events.

In the present study, the most common drugs causing rash were leflunomide followed by gliclazide and paracetamol. While zidovudine was most notorious for already labelled as associated ADRs such as anaemia, myalgia, nausea and abdominal pain. Anaemia is found to be associated with both the dose and stage of disease and myopathy is the most significant yet, manageable type of late adverse reaction [28].

The combination of tenofovir, lamivudine and efavirenz was most commonly associated with vomiting, dizziness, anxiety and gynaecomastia. Among these ADRs association with

For peer review only - http://bmjopen.bmj.com/site/about/guidelines.xhtml

dizziness draws attention. It has been found that those patients who did not report such ADRs to the attending physicians end up having severe diseases. The debilitating nature of the symptoms and awareness levels of the patients about, what should report to the attending doctor, plays an important role in reporting ADRs. Therefore, increasing awareness of patients is of utmost importance via asking direct questions about the ADRs caused by the flagged drugs [29,30]. In addition, a plan could be developed to disseminate the findings of the pharmacovigilance surveillance to the prescribers so that they can avoid prescribing the drugs responsible for ADRs, wherever possible. In cases where it is not possible to avoid the drug, for example in ART, the prescribers can counsel patients based on the associations found via pharmacovigilance. This will help to improve patient's compliance and will motivate patients to seek early advice in case of occurrence of ADRs.

Polypharmacy is prevalent globally and the occurrence of ADR is directly proportional to the number of prescribed drugs thus, polypharmacy must be discouraged [31,32]. However, in the present study single drug was attributed for most of the ADRs (73%). It was observed that in most of the reports the drug that was most suspected of being associated with the specific ADR was reported and not the whole treatment. NSAIDs are often a constituent of many rational and irrational fixed-dose combinations and this could be a cause of the high incidence of the ADRs related to the NSAIDs [33]. This was reflected in our study where besides antimicrobials and ART drugs the antiepileptics and NSAIDs also caused substantial ADRs [19-22, 34-38]. In the present study, the most prescribed pharmacological substances among the patients having ADRs were zidovudine, ethambutol and ciprofloxacin while in other studies ceftriaxone, zidovudine, and phenytoin sodium were the most prescribed drugs [34-38]. Zidovudine and ethambutol are part of multidrug ATT and ART regimes, respectively. Factors including the dose of the drugs, drug-drug interactions as well as genetic differences of the study population could also affect the number of adverse drug reactions caused by drugs [39].

For peer review only - http://bmjopen.bmj.com/site/about/guidelines.xhtml

Most of the patients had mild ADRs in our study, as observed in other Indian studies [15,17]. ADRs of skin and subcutaneous tissue disorders (28%) were most common among all SOCs. Skin rashes were the most common complaints followed by gastrointestinal complaints (18%), nervous system disorders (11%), general disorders (10%) and psychiatric disorders (7%). Skin and subcutaneous tissues are among the most targeted organs for ADRs. The disorders are reported more frequently because these develop within one week of drug administration and can easily be spotted when the patients are still under the close supervision of the healthcare providers [15,40,41]. Timely recognition of skin ADRs is crucial for its classification, management, and causality assessment.

The causality assessment was conducted based on the WHO probability scale. According to the assessment, most cases could be categorized as 'Possible' (48) followed by 'Probable' (43). ADRs are rarely categorised as 'Certain' as it requires to re-challenge the patient with the same causative drug, which is not ethical, hence the assessment frequently goes to the Possible and Probable category [15,42]. Though 26 drugs were associated with serious, life-threatening ADRs, yet none of the drug classes could be implicated for serious ADRs. This was because of insufficient data due to the under-reporting of ADRs, and the wide range of drugs prescribed. An adequate ADR reporting is required to preserve the clinical information that could prevent significant damage to patients, plan and implement measures to reduce their impact on public health and consequently minimize the burden on the health care system. Under-reporting a challenge in pharmacovigilance when other AMC contributed with 212 ADRs per month our AMC reported an average of 19 ADRs per month in 2016 while dropped to 11 in 2019 [43].

Early identification and management of ADRs will not only improve patient safety, quality of life but also reduce the cost of treatment. Labelling of new ADRs will help to prepare the prescribing policies and modify the prescribing patterns. Reduction in prescribing of the flagged drugs will reduce the prevalence of ADRs. We believe that disseminating results to the

For peer review only - http://bmjopen.bmj.com/site/about/guidelines.xhtml

prescribers through feedback might reduce prescribing of causative drugs and improve reporting of ADRs. A knowledge, attitude and practice-based study conducted in Norway found that ADRs can be prevented by collecting reliable information about their frequencies and possible risk factors [7]. Therefore, we propose to develop and implement strategies for the community but specifically for the HCWs to improve reporting and monitoring of ADRs. We also propose to disseminate the study results in the form of a feedback method to improve reporting. This might also help in the reduction of prescription of the drugs causing most of the ADRs. The methods of the feedback study could be shared and tested at other similar settings as well.

Strengths and limitations of this study

This is the first long term study that presents ADR data reported from an AMC in Central India. The spontaneous self- and voluntary-reporting study design used is easy and low resource-demanding. The results of the present study could be used to implicate drugs that most notoriously caused the ADRs. Our study has few limitations as well. Since the ADRs were recorded spontaneously, there was no option to follow the patient or patient's record to confirm the final outcome, later. The data was collected based on spontaneous reporting rather than active surveillance, which would have been a better method. Another limitation is that we could not estimate the precise incidence of suspected ADRs attributable to a particular drug. However, this was due to fewer reported cases of ADRs and could not be controlled.

Conclusion

The maximum number of ADRs were reported from the breadwinner group (males and 19-65 years). Antimicrobials caused the maximum number of ADRs. This highlights an alarming situation in addition to antimicrobial resistance. Prudent use of antimicrobials is once again proven as need of the hour. ADRs of skin and subcutaneous tissue disorders were most frequent

For peer review only - http://bmjopen.bmj.com/site/about/guidelines.xhtml

among all SOCs. Most of the ADRs were mild and belonged to the ‘Possible’ group of probability scale. However, under-reporting of ADRs at our AMC is evident and a major factor for the pause in performing severity and causality assessment. Thus, the prevalence of ADRs reported in the present study can be perceived as the tip of an iceberg.

To deal with the problem of under-reporting we suggest continuous training programmes for HCPs, seeking the collaboration of nearby health care facilities, and most importantly giving feedback to the HCWs to impart confidence, awareness and knowledge about the ADR causing drugs and will encourage reporting ADR events.

Future implications

Our results indicate the need to develop and test a model of motivational educational- approach focusing on health care workers and the community to increase ADR reporting in the settings associated with our AMC. Neither the global pharmacovigilance nor the PvPI has designed a plan, to analyse and provide feedback on the ADR data to the prescribers. Providing reliable and balanced information, and a valid assessment of the risk-benefit profile of the marketed drugs to the healthcare professionals at a local level is essential. This knowledge might improve patients’ safety while prescribing drugs, support patient care and public health programs in the settings. Therefore, we propose to develop a model with a strategy to provide feedback and knowledge to improve ADR reporting. Furthermore, providing feedback to the prescribers and sensitizing them about the flagged drugs will be an encouragement for reporting ADRs. HCPs will be assured that reporting ADRs has no legal implications.

Modification of the organizational structure, training of HCPs and availability of resources can improve early detection of ADRs. We also suggest planning and start an ADR awareness and training campaign in collaboration with HCPs of the hospitals in the periphery, district, and private hospitals to motivate them to report ADRs. However, the long-term sustainability of

For peer review only - http://bmjopen.bmj.com/site/about/guidelines.xhtml

these measures is most crucial as the effectiveness of interventions diminishes with time. The use of a digital platform such as SMS and E-mails might play an important role in reminding and keeping them motivated and sensitized to report ADRs timely.

Acknowledgement

The authors acknowledge the active support of the consultants and physicians in data collection. The Authors extend thanks to the IPC PvPI, Ghaziabad for encouraging and permitting the publication. Thanks are due to the management of the AMC- RDGMC for their support. The authors also acknowledge Mr Amit Pawar for his assistance during the study.

Author Contributions

MS and RB conceptualized the study and designed the analyses. ST, MS, RB and SA collected the data. MS and RB performed the analyses and drafted the manuscript. All authors contributed to and approved the present form of the manuscript.

Funding statement

This research received no specific grant from any funding agency in the public, commercial, or not-for-profit sectors

Competing interest

The authors declare no competing interests.

Data Sharing statement

As per the protocol of the institutional ethics committee to maintain full confidentiality, the data can be made available through a request form addressing, The Chairman, Ethics Committee, R.D. Gardi Medical College, Agar Road, Ujjain, Madhya Pradesh, India 456006

For peer review only

(email: iecrdgmc@yahoo.in, uctharc@sancharnet.in), giving all details of the article. Quote the IEC approval number: 68/2019 along with the request.

Licence statement

The Corresponding Author has the right to grant on behalf of all authors and does grant on behalf of all authors, an exclusive licence on a worldwide basis to the BMJ Publishing Group Ltd ("BMJ"), and its Licensees to permit this article (if accepted) to be published in The BMJ's editions and any other BMJ products and to exploit all subsidiary rights.

The TRIPOD checklist indicating the page and line numbers of our manuscript is included with the cover letter.

For peer review only

References

1. Vivekanandan K, Thota P, Janarthanan VV, Singh GN. Pharmacovigilante's in the pharmacovigilance programme of India: Ideal qualities and skills. J Young Pharm 2016;8:291-2. (Assessed 08 April 2021)
2. Nirojini PS, Yemineni R, Nadenla RR. Monitoring and Reporting of Adverse Drug Reactions in A South Indian Tertiary Care Hospital. Int J Pharm Sci Rev Res. 2014;24(1):259-62.
3. Datta S, Sengupta S. An evaluation of knowledge, attitude, and practice of adverse drug reaction reporting in a tertiary care teaching hospital of Sikkim. Perspect Clin Res 2015;6:200-6.
4. Mittal N, Mittal R, Gupta MC. An overview of the pharmacovigilance system in India. Clin Res Regul Aff 2016;33:4-8.
5. Tandon VR, Mahajan V, Khajuria V, Gillani Z. Under-reporting of adverse drug reactions: a challenge for pharmacovigilance in India. Indian J Pharmacol. 2015;47(1):65-71. doi:10.4103/0253-7613.150344 (Assessed 08 April 2021)
6. Hazell L, Shakir SAW. Under-reporting of adverse drug reactions: A systematic review. Drug Saf. 2006; 29 (5) :385-96.366
7. Castberg I, Reimers A, Sandvik P, Aamo TO, Spiqset O. Adverse drug reactions of antidepressants and antipsychotics: Experience, knowledge and attitudes among Norwegian psychiatrists. Nord J Psychiatry. 2006;60:227–33.
8. Indian Pharmacopoeia Commission, Ministry of Health and family Welfare, Govt. of India. www.ipc.gov.in (Assessed 08 April 2021)
9. ADR form PvPI. Available from: http://www.cdsco.nic.in/writereaddata/ADR%20form%20PvPI.pdf. (Assessed 08 April 2021)

For peer review only - http://bmjopen.bmj.com/site/about/guidelines.xhtml

10. IA Eland, KJ Belton, AC van Grootheest, AP Meiners, MD Rawlins, and BH Ch Stricker. Attitudinal survey of voluntary reporting of adverse drug reactions British journal of clinical Pharmacology1999 Oct; 48(4): 623–627.doi: 10.1046/j.1365-2125.1999.00060.x (Assessed 08 April 2021)

11. Schumock GT, Seeger JD, Kong SX. Control charts to monitor rates of adverse drug reactions. Hosp Pharm 1995;30:1088, 1091‑2,1095‑6.

12. The use of the WHO–UMC system for standardised case causality assessment. Accessed from: http://www.WHO-UMC.org/graphics/4409.pdf (Assessed 08 April 2021)

13. Hartwig SC, Siegel J, Schneider PJ. Preventability and severity assessment in reporting adverse drug reactions. Am J Hosp Pharm 1992;49:2229‑32.

14. Daulat MP, Abishake VJ, Singh P, Raj B. A prospective study of adverse drug reactions in a tertiary care teaching hospital. Int J Basic Clin Pharmacol 2018;7:1965-69.

15. Ramakrishnaiah H et al. Int J Basic Clin Pharmacol. 2015 Jun;4(3):515-521 International Journal of Basic & Clinical Pharmacology.

16. Singh P, Agrawal M, Hishikar R, Joshi U, Maheshwari B, Halwai A. Adverse drug reactions at adverse drug reaction monitoring center in Raipur: Analysis of spontaneous reports during 1 year. Indian J Pharmacol. 2017;49(6):432-437. doi:10.4103/ijp.IJP_781_16 (Assessed 08 April 2021)

17. Thakare VS, Kavitha V D, Langade D. Prospective observational study to evaluate adverse drug reactions pattern in a tertiary level teaching hospital. Natl J Physiol Pharm Pharmacol 2019;9(5):434-437.

18. Landstedt K, Stålsby-Lundborg C, Sharma A, Johansson F, Sharma M. Antibiotic prescriptions to the inpatients having non-bacterial diagnosis at medicine departments of two private sector hospitals in Madhya Pradesh, India: a cross sectional study. *BMJ Open*

For peer review only - http://bmjopen.bmj.com/site/about/guidelines.xhtml

2017;7:e012974. doi:10.1136/bmjopen-2016- 012974. Available at URL- http://bmjopen.bmj.com/content/bmjopen/7/4/e012974.full.pdf (Assessed 08 April 2021)

19. Anna Makhowska, Jonatan Sparentofft, Cecilia Stålsby Lundborg, Megha Sharma. Perioperative antibiotic prescribing in surgery departments of two private sector hospitals in Madhya Pradesh, India. Perioperative Medicine. (2019) 8:10. DOI: https://doi.org/10.1186/s13741-019-0121-3 and https://doi.org/10.1186/s13741-019-0123-1 (Assessed 08 April 2021)
20. Wester K, Jönsson AK, Spigset O, Druid H, Hägg S. Incidence of fatal adverse drug reactions: a population based study. Br J Clin Pharmacol. 2008;65:573-9.
21. Gor AP, Desai SV. Adverse drug reactions (adr) in the inpatients of medicine department of a rural tertiary care teaching hospital and influence of pharmacovigilance in reporting ADR. Indian J Pharmacol. 2008;40(1):37-40.
22. Vora MB, Trivedi HR, Shah BK, Tripathi CB. Adverse drug reactions in inpatients of internal medicine wards at a tertiary care hospital: a prospective cohort study. J Pharmacol Pharmacother. 2011;2(1):21-5.
23. Habarugira, JMV, Figueras, A. Pharmacovigilance network as an additional tool for the surveillance of antimicrobial resistance. Pharmacoepidemiol Drug Saf. 2021; 30: 1123–1131. https://doi.org/10.1002/pds.5249
24. Shet A, Antony J, Arumugam K, Kumar Dodderi S, Rodrigues R, DeCosta A (2014) Influence of Adverse Drug Reactions on Treatment Success: Prospective Cohort Analysis of HIV-Infected Individuals Initiating First-Line Antiretroviral Therapy in India. PLoS ONE 9(3): e91028. https://doi.org/10.1371/journal.pone.0091028 (Assessed 08 April 2021)

For peer review only - http://bmjopen.bmj.com/site/about/guidelines.xhtml

25. Manju R, Maheshwari YN, Ramya JE, Krishnamoorthy K. Adverse drug reaction monitoring of antitubercular drugs during intensive phase at tertiary care medical college hospital: A prospective study. Natl J Physiol Pharm Pharmacol 2020;10(11):976-980.

26. Mukherjee S, Era N, Saha B, Tripathi SK. Adverse drug reaction monitoring in patients on antiretroviral therapy in a tertiary care hospital in Eastern India. *Indian J Pharmacol*. 2017;49(3):223-228. doi:10.4103/ijp.IJP_304_16 (Assessed 08 April 2021)

27. Abah IO, Ncube NBQ, Bradley HA, AgbaJi OO, Kanki P. Antiretroviral Therapy-associated Adverse Drug Reactions and their Effects on Virologic Failure- A Retrospective Cohort Study in Nigeria. Curr HIV Res. 2018;16(6):436-446. doi:10.2174/1389450120666190214144609 (Assessed 08 April 2021)

28. Rachlis A, Fanning MM. Zidovudine toxicity. Clinical features and management. Drug Saf. 1993 Apr;8(4):312-20. doi: 10.2165/00002018-199308040-00005. PMID: 8481217. (Assessed 10 August 2021)

29. Khoza-Shangase K. VESTIBULAR FUNCTION IN A GROUP OF ADULTS WITH HIV/AIDS ON HAART. Afr J Infect Dis. 2017;12(1):7-14. Published 2017 Nov 15. doi:10.21010/ajid.v12i1.2 (assessed 10 August 2021)

30. Molla Y, Wubetu M, Dessie B. Anti-Tuberculosis Drug Induced Hepatotoxicity and Associated Factors among Tuberculosis Patients at Selected Hospitals, Ethiopia. Hepat Med. 2021;13:1-8 https://doi.org/10.2147/HMER.S290542 (Assessed 10 August 2021)

31. Nguyen JK, Fouts MM, Kotabe SE, Lo E. Polypharmacy as a risk factor for adverse drug reactions in geriatric nursing home residents. Am J Geriatr Pharmacother. 2006 Mar;4(1):36-41. doi: 10.1016/j.amjopharm.2006.03.002. PMID: 16730619. (Assessed 08 April 2021)

32. R., Siddarama, Bharath Naidu J., Joshisree K. P., and Sahithi Lakshmi V. POLYPHARMACY INDUCED DRUG INTERACTIONS, ADVERSE DRUG

For peer review only - http://bmjopen.bmj.com/site/about/guidelines.xhtml

REACTIONS (ADR) AND MEDICATION ERRORS IN TERTIARY CARE SOUTH INDIAN HOSPITAL. International Journal of Pharmacy and Pharmaceutical Sciences, Vol. 11, no. 2, Feb. 2019, pp. 88-93, doi:10.22159/ijpps.2019v11i2.29508. (Assessed 09 August 2021)

33. Gor AP, Saksena M. Adverse drug reactions of nonsteroidal anti-inflammatory drugs in orthopedic patients. J Pharmacol Pharmacother. 2011;2(1):26-29. doi:10.4103/0976-500X.77104 (Assessed 08 April 2021)

34. Kathiria JM, Sattigere BM, Desai PM, Patel SP. A study of adverse drug reactions in patients admitted to intensive care unit of a tertiary care teaching rural hospital. Int J Pharm Pharm Sci. 2013;5(1):160-3.
35. Sharma SK. Zidovudine-induced anaemia in HIV/AIDS. Indian J Med Res. 2010;132:359-61.
36. Shah SP, Desai MK, Dikshit RK. Analysis of cutaneous adverse drug reactions at a tertiary care hospital: a prospective study. Trop J Pharm Res. 2011;10(4):517-22.
37. Vijendra R, Pundarikaksha HP, Gopal MG, Girish K, Vasundara K, Jyothi R. A prospective study of cutaneous adverse drug reaction in a tertiary care hospital. Natl J Basic Med Sci. 2013;3(1):44-51.
38. Hussain MM, Girhepunje K, Pal R, Siddiqua SS. Incidence of adverse drug reactions in a tertiary care hospital: a systematic review and meta-analysis of prospective studies. Pharm Lettr. 2010;2(3):358-68.
39. Hema NG, Bhuvana KB, Virupaksha HM. Critical Assessment of Adverse drug Reactions to Antitubercular Drugs in a Government Teaching Hospital. IJBMS, 2013; 4(2):60-67.
40. Arulmani R, Rajendran SD, Suresh B. Adverse drug reaction monitoring in a secondary care hospital in South India. Br J Clin Pharmacol. 2008;65(2):210-6.

For peer review only

41. Modi A, Desai M, Shah S, Shah B. Analysis of Cutaneous Adverse Drug Reactions Reported at the Regional ADR Monitoring Center. Indian J Dermatol. 2019;64(3):250. doi:10.4103/ijd.IJD_682_16 https://www.ncbi.nlm.nih.gov/pmc/articles/PMC6537693/ (Assessed 08 April 2021)

42. Laskar JI, Chakravarty P, Dewan B. A study on incidence of adverse drug reactions with commonly prescribed drugs and causality assessment in Silchar Medical College and Hospital. Int J Basic Clin Pharmacol 2017;6:1175-83.

43. Tandon VR, Mahajan V, Khajuria V, Gillani Z. Under-reporting of adverse drug reactions: a challenge for pharmacovigilance in India. Indian J Pharmacol. 2015;47(1):65-71. doi:10.4103/0253-7613.150344

For peer review only

Figure 1 Frequency of type of ADRs reported per year at an AMC in Central India

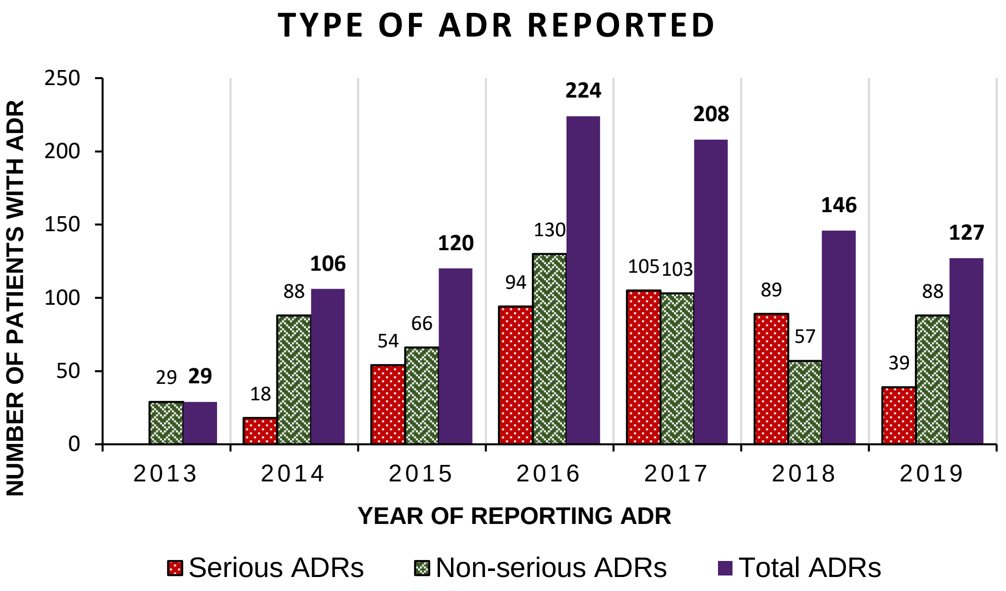

ADR=Adverse Drug Reaction, AMC=ADR Monitoring Centre

Figure 2 Occurrence of ADRs related to the pharmacological drug classes at an AMC in Central India

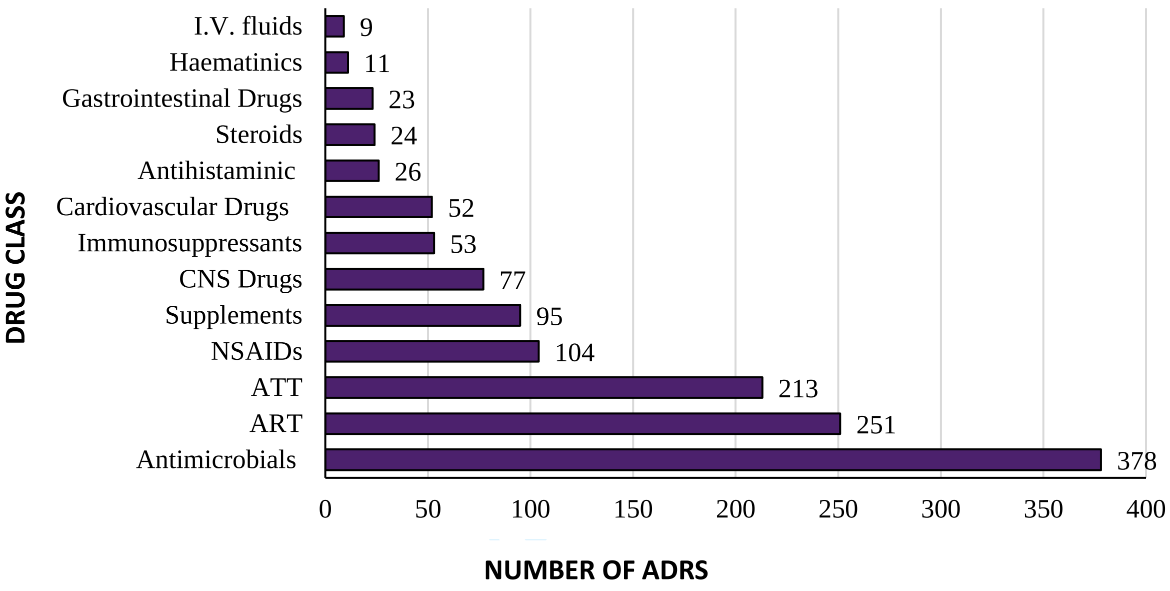

ADR=Adverse Drug Reaction, AMC=ADR Monitoring Centre ART=Antiretroviral therapy, ATT=Antitubercular therapy, CNS=Central Nervous System, NSAID= Non-steroidal anti-inflammatory drugs, I.V. fluids= Intravenous fluid

For peer review only - http://bmjopen.bmj.com/site/about/guidelines.xhtml

For peer review only

Figure 3: Pharmacological substances implicated in causing ADRs at an AMC in Central India

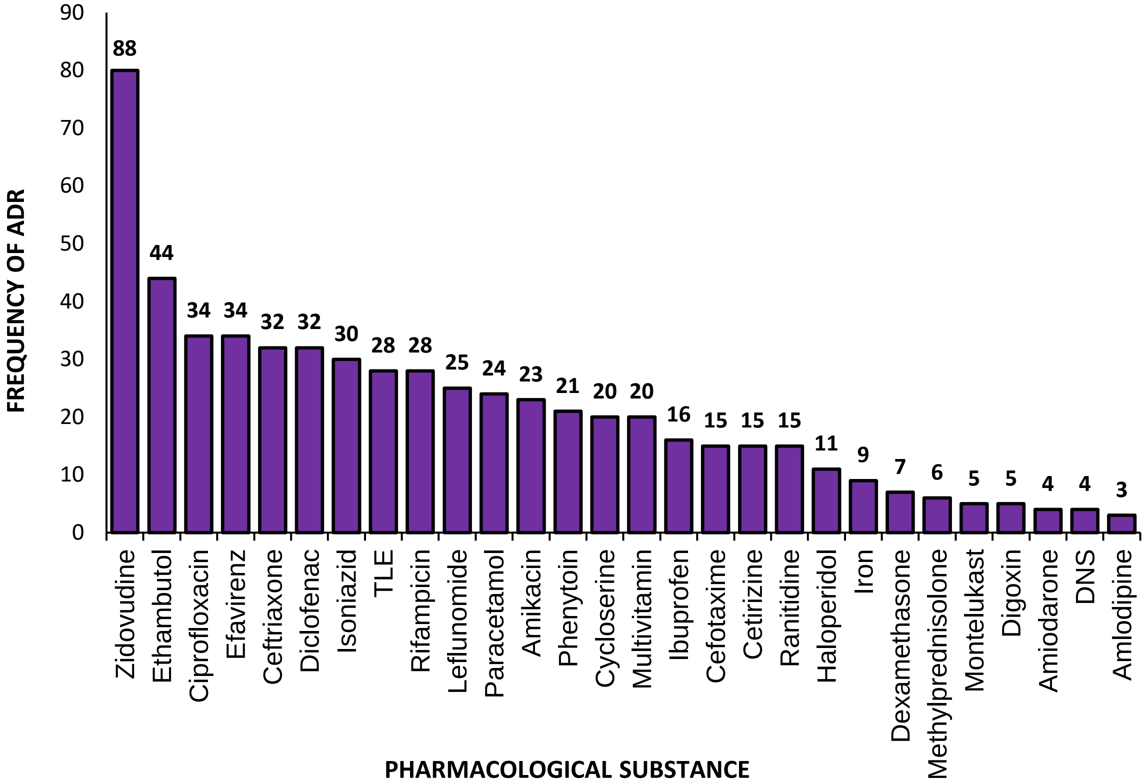

ADR=Adverse Drug Reaction, AMC=ADR Monitoring Centre, DNS=Dextrose Normal Saline, T/L/E=Tenofovir/Lamivudine/ Efaviren

ANNEXURE – I

Version-1.3

SUSPECTED ADVERSE DRUG REACTION REPORTING FORM

For VOLUNTARY reporting of Adverse Drug Reaction by Healthcare Professionals

INDIAN PHARMACOPOEIA COMMISSION (National Coordination Centre-Pharmacovigilance Programme of India)
Ministry of Health & Family Welfare, Government of India Sector-23, Raj Nagar, Ghaziabad-201002

A. PATIENT INFORMATION

1. Patient Initials ________	2. Age at the time of Event or Date of Birth ________	3. M ☐ F ☐ Other ☐<br>4. Weight________Kgs

Reg. No. /IPD No. /OPD No. /CR No. :

AMC Report No. :

Worldwide Unique No. :

B. SUSPECTED ADVERSE REACTION

5. Event/Reaction start date (dd/mm/yyyy)

6. Event/Reaction stop date (dd/mm/yyyy)

6 (A). Onset Lag Time

7. Describe Event/Reaction with treatment details, if any

12. Relevant tests/ laboratory data with dates

13. Relevant medical/medication history (e.g. allergies, race, pregnancy, smoking, alcohol use, hepatic/renal dysfunction, past surgery etc.)

14. Seriousness of the reaction: No ☐ if Yes ☐(please tick anyone)

☐ Death (dd/mm/yyyy) ☐ Congenital-anomaly
☐ Life threatening ☐ Disability
☐Hospitalization/Prolonged ☐ Other Medically important

15. Outcomes

☐ Recovered ☐ Recovering ☐ Not recovered
☐ Fatal ☐ Recovered with sequelae ☐ Unknown

C. SUSPECTED MEDICATION(S)

S.No	8. Name (Brand/Generic)	Manufacturer (if known)	Batch No. / Lot No.	Exp. Date (if known)	Dose used	Route used	Frequency (OD, BD etc.)	Therapy dates		Indication	Causality Assessment
								Date started	Date stopped		
i											
ii											
iii											
iv*											

S.No as per C	9. Action Taken (please tick)						10. Reaction reappeared after reintroduction (please tick)			
	Drug withdrawn	Dose increased	Dose reduced	Dose not changed	Not applicable	Unknown	Yes	No	Effect unknown	Dose (if reintroduced)
i										
ii										
iii										
iv										

11. Concomitant medical product including self-medication and herbal remedies with therapy dates (Exclude those used to treat reaction)

S.No	Name (Brand/Generic)	Dose used	Route used	Frequency (OD, BD, etc.)	Therapy dates		Indication
					Date started	Date stopped	
i							
ii							
iii*							

Additional Information:

D. REPORTER DETAILS

16. Name and Professional Address:______________________________
____
Pin:____________E-mail______________________________
Tel. No. (with STD code)______________________________
Occupation:__________________ Signature:________________

17. Date of this report (dd/mm/yyyy):

Sig. and Name of Receiver-

Confidentiality: The patient's identity is held in strict confidence and protected to the fullest extent. Submission of a report does not constitute an admission that medical personnel or manufacturer or the product caused or contributed to the reaction. Submission of an ADR report does not have any legal implication on the reporter.

*use separate page for more information

For peer review only - http://bmjopen.bmj.com/site/about/guidelines.xhtml

TRIPOD Checklist: Prediction Model Development

Section/ Topic	Item	Checklist Item	Page
**Title and abstract**			
Title	1	Identify the study as developing and/or validating a multivariable prediction model, the target population, and the outcome to be predicted.	1
Abstract	2	Provide a summary of objectives, study design, setting, participants, sample size, predictors, outcome, statistical analysis, results, and conclusions.	2
**Introduction**			
Background and objectives	3a	Explain the medical context (including whether diagnostic or prognostic) and rationale for developing or validating the multivariable prediction model, including references to existing models.	3
	3b	Specify the objectives, including whether the study describes the development or validation of the model or both.	4
**Methods**			
Source of data	4a	Describe the study design or source of data (e.g., randomized trial, cohort, or registry data), separately for the development and validation data sets, if applicable.	4
	4b	Specify the key study dates, including start of accrual; end of accrual; and, if applicable, end of follow-up.	4
Participants	5a	Specify key elements of the study setting (e.g., primary care, secondary care, general population) including number and location of centres.	4
	5b	Describe eligibility criteria for participants.	4
	5c	Give details of treatments received, if relevant.	4,5
Outcome	6a	Clearly define the outcome that is predicted by the prediction model, including how and when assessed.	4-6
	6b	Report any actions to blind assessment of the outcome to be predicted.	NA
Predictors	7a	Clearly define all predictors used in developing or validating the multivariable prediction model, including how and when they were measured.	NA
	7b	Report any actions to blind assessment of predictors for the outcome and other predictors.	7
Sample size	8	Explain how the study size was arrived at.	NA
Missing data	9	Describe how missing data were handled (e.g., complete-case analysis, single imputation, multiple imputation) with details of any imputation method.	7
Statistical analysis methods	10a	Describe how predictors were handled in the analyses.	7
	10b	Specify type of model, all model-building procedures (including any predictor selection), and method for internal validation.	NA
	10d	Specify all measures used to assess model performance and, if relevant, to compare multiple models.	NA
Risk groups	11	Provide details on how risk groups were created, if done.	NA
**Results**			
Participants	13a	Describe the flow of participants through the study, including the number of participants with and without the outcome and, if applicable, a summary of the follow-up time. A diagram may be helpful.	6
	13b	Describe the characteristics of the participants (basic demographics, clinical features, available predictors), including the number of participants with missing data for predictors and outcome.	8-11
Model development	14a	Specify the number of participants and outcome events in each analysis.	8
	14b	If done, report the unadjusted association between each candidate predictor and outcome.	NA
Model	15a	Present the full prediction model to allow predictions for individuals (i.e., all regression coefficients, and model intercept or baseline survival at a given time point).	NA

For peer review only

TRIPOD Checklist: Prediction Model Development

specification	15b	Explain how to the use the prediction model.	17
Model performance	16	Report performance measures (with CIs) for the prediction model.	NA
**Discussion**			
Limitations	18	Discuss any limitations of the study (such as nonrepresentative sample, few events per predictor, missing data).	2,16
Interpretation	19b	Give an overall interpretation of the results, considering objectives, limitations, and results from similar studies, and other relevant evidence.	12-17
Implications	20	Discuss the potential clinical use of the model and implications for future research.	16-18
**Other information**			
Supplementary information	21	Provide information about the availability of supplementary resources, such as study protocol, Web calculator, and data sets.	NA
Funding	22	Give the source of funding and the role of the funders for the present study.	18

We recommend using the TRIPOD Checklist in conjunction with the TRIPOD Explanation and Elaboration document.

For peer review only

BMJ Open

Surveillance of adverse drug reactions at an adverse drug reaction monitoring centre in Central India: A seven-year surveillance study

Journal:	*BMJ Open*
Manuscript ID	bmjopen-2021-052737.R2
Article Type:	Original research
Date Submitted by the Author:	16-Sep-2021
Complete List of Authors:	Sharma, Megha; Karolinska Institutet, Department of Global Public Health; RD Gardi Medical College, Department of Pharmacology<br>Baghel, Ruchi; RD Gardi Medical College, Department of Pharmacology<br>Thakur, Sunil; RD Gardi Medical College, Department of Pharmacology<br>Adwal, Sandeep; Zydus Medical College and Hospital, Department of Pharmacology
**Primary Subject Heading**:	Pharmacology and therapeutics
Secondary Subject Heading:	Pharmacology and therapeutics, Public health, Medical education and training, Infectious diseases, Global health
Keywords:	CLINICAL PHARMACOLOGY, Health & safety < HEALTH SERVICES ADMINISTRATION & MANAGEMENT, Risk management < HEALTH SERVICES ADMINISTRATION & MANAGEMENT, Adverse events < THERAPEUTICS, Toxicity < THERAPEUTICS, Pharmacology < TROPICAL MEDICINE

SCHOLARONE™
Manuscripts

I, the Submitting Author has the right to grant and does grant on behalf of all authors of the Work (as defined in the below author licence), an exclusive licence and/or a non-exclusive licence for contributions from authors who are: i) UK Crown employees; ii) where BMJ has agreed a CC-BY licence shall apply, and/or iii) in accordance with the terms applicable for US Federal Government officers or employees acting as part of their official duties; on a worldwide, perpetual, irrevocable, royalty-free basis to BMJ Publishing Group Ltd ("BMJ") its licensees and where the relevant Journal is co-owned by BMJ to the co-owners of the Journal, to publish the Work in this journal and any other BMJ products and to exploit all rights, as set out in our licence.

The Submitting Author accepts and understands that any supply made under these terms is made by BMJ to the Submitting Author unless you are acting as an employee on behalf of your employer or a postgraduate student of an affiliated institution which is paying any applicable article publishing charge ("APC") for Open Access articles. Where the Submitting Author wishes to make the Work available on an Open Access basis (and intends to pay the relevant APC), the terms of reuse of such Open Access shall be governed by a Creative Commons licence – details of these licences and which Creative Commons licence will apply to this Work are set out in our licence referred to above.

Other than as permitted in any relevant BMJ Author's Self Archiving Policies, I confirm this Work has not been accepted for publication elsewhere, is not being considered for publication elsewhere and does not duplicate material already published. I confirm all authors consent to publication of this Work and authorise the granting of this licence.

For peer review only

Surveillance of adverse drug reactions at an adverse drug reaction monitoring centre in Central India: A seven-year surveillance study

Authors: Megha Sharma[#*1,2], Ruchi Baghel*[1], Sunil Thakur[1], Sandeep Adwal[3]

[1]Department of Global Public Health- Health Systems and Policy: Medicines, Karolinska Institutet, Stockholm, Sweden. 171 77

[2]Department of Pharmacology, R. D. Gardi Medical College, Ujjain, Madhya Pradesh, India. 456006

[3]Department of Pharmacology, Zydus Medical College and Hospital, Nimnaliya, Dahod, Gujarat. 389151

*Contributed equally

[#] Corresponding author

Email:

Megha Sharma: megha.sharma@ki.se

Ruchi Baghel: drruchibaghel@gmail.com

Sunil Thakur: sunilcsthakur@gmail.com

Sandeep Adwal: adwalsandeep@yahoo.co.in

Corresponding author

[#] Associate Professor, Department of Pharmacology

R. D. Gardi Medical College Ujjain, (MP), India

ZIP/PIN 456006

Keywords: Pharmacovigilance, Adverse drug reactions, ADR Monitoring Centre, spontaneous ADRs, Antimicrobials, Antiretroviral therapy, Antitubercular therapy, India

For peer review only - http://bmjopen.bmj.com/site/about/guidelines.xhtml

Abstract

Objectives

To analyse and present the occurrence and severity of spontaneous ADRs reports prospectively registered at an AMC in Central India.

Setting and data

The survey was conducted between 2013 and 2019 at an ADR Monitoring Centre in Central India. ADRs were recorded using the standard 'Suspected ADR Reporting form'.

Outcome measures: The causality of the ADRs were categorized using the WHO causality assessment scale to assess the relationship between a drug and the occurrence of an ADR.

Results

Totally 1980 spontaneous ADRs were reported involving 960 patients and 1316 drugs prescriptions. The occurrence of ADRs was common among male patients (64%) and patients of age between 19-65 years (81%). Antimicrobials caused 29% ADRs, followed by drugs of Antiretroviral therapy (19%). Zidovudine caused most ADRs (88%) followed by ethambutol and ciprofloxacin. The ADRs of skin and subcutaneous tissue disorders (28%) were most common among all system-organ classes followed by gastrointestinal systems (18%). Four percent of all reported ADRs were severe. A peak of ADR reports was attained in 2016 with 224 reports which decreased to 127 in 2019.

Conclusion

A high number of ADRs caused by antimicrobials is an alarming situation, which adds up to antimicrobial resistance. Judicious use of antimicrobials is yet again proven as need of the hour. Under-reporting of ADRs is evident in our study and is a major factor for the delay in the withdrawal of drugs responsible for causing ADRs. Interventions in terms of training and feedback are suggested to encourage and improve ADR reporting.

For peer review only

Article summary

Strengths and limitations of this study

- This is the first long-term study that presents the ADRs reported from Central India.
- The spontaneous self- and voluntary-reporting study design used is easy and low resource-demanding.
- The data was collected based on spontaneous reporting rather than active surveillance which would have been a better method to overcome the issue of under-reporting.
- There was no option to follow the patient or patient's record to confirm the final outcome, if not mentioned.
- The space provided in ADR form is insufficient to record details of antibiotic susceptibility test reports, crucial to generate signals related to antibiotic resistance.

For peer review only

For peer review only - http://bmjopen.bmj.com/site/about/guidelines.xhtml

Background

Pharmacovigilance is a process of post-marketing surveillance of drugs–that continues throughout the drug lifecycle. It is essential in analysing and managing the risks associated with drugs that are available for the use of the general population [1]. The results of pharmacovigilance certify the effectiveness and safety of drugs in terms of adverse drug reactions (ADRs). The occurrence of ADRs can prolong hospitalization and thereby increase the cost of hospital stay and treatment and initiate the requirement of additional clinical investigations in severe cases. World health organization (WHO) defines 'Pharmacovigilance' as the science and activities relating to the detection, assessment, understanding, and prevention of adverse effects or any other possible drug-related problems, including herbal medicines [2]. The WHO Collaborating Centre (WHOCC) for International Drug Monitoring at Uppsala Monitoring Centre (UMC), Sweden; promotes pharmacovigilance at the national level. The national data from the participating countries are shared with the UMC and compiled to generate a global adverse drug reaction (ADR) database. The ADR database is collected from various countries under the WHOCC ADR monitoring program and compiled together to generate signals or flags. A signal or flag is a presumed risk associated with the use of a drug and is supported by reliable data sources. Based on the signals or flags, recommendations are made for regulatory interventions at the international level in the form of labelling revisions or banning the drug and communicating the evident risks to the policymakers. India is one of the global partners in the global program and participates under the Ministry of family welfare via the Pharmacovigilance Programme of India (PvPI).

The PvPI is a WHO initiative to scrutinize drug-induced mortality and morbidity in India. The PvPI is a collaborative project between WHO and Central Drugs Standard Control Organization (CDSCO), Ministry of Health and Family Welfare in India to prevent the ADRs. Collection, reporting and follow-up of the adverse drug reactions (ADRs) occurring among the

For peer review only - http://bmjopen.bmj.com/site/about/guidelines.xhtml

patients are prime activities included under PvPI. ADR Monitoring Centres (AMCs) are basic units of the PvPI, and it works at the national level intending to identify, analyse, characterize, and estimate the extent of the problem associated with drug use. The program aims to improve the vigilance on drugs, enhance patient safety and achieve better health benefits [3,4]. Reporting ADRs at an institutional level provide valuable information about potential problems during drug usage in healthcare settings. However, the reports of signals or flags generated at UMC are not publicized to the AMCs. Moreover, there is a wide gap between the occurrence and reporting of ADRs globally [5,6]. A study conducted in Norway concludes that ADRs can be prevented by collecting reliable information about their frequencies and possible risk factors. [7] Thus, it is of utmost importance to collect and analyse the prevalence of ADR to; a) identify the potential risks with drugs, b) support management of diseases and, c) rationalise the prescribing practices.

Objectives

The study was conducted to analyse and present the occurrence and severity of spontaneous ADRs reports registered at an AMC in Central India.

Methods

Study design and setting

This non-interventional survey was a part of PvPI and presents the data collected from one of the AMC under PvPI. All ADR reports received from the hospitals and healthcare facilities between July 2013 and December 2019, at the AMC- R. D. Gardi Medical College, Ujjain in Central India were included in the study. The Medical College, established in the year 2000, is the first private medical college in the Madhya Pradesh state of India. The institute is recognised as an AMC since 2012 [8].

Data collection considerations and analysis

For peer review only - http://bmjopen.bmj.com/site/about/guidelines.xhtml

The Suspected ADR Reporting form, recommended by the CDSCO was used as the data collection tool (Annexure I) [9,10]. The suspected ADRs were spontaneously reported by the Health Care Professionals (HCPs), patients or patient's relatives; to our AMC either directly or through the Patient Safety Pharmacovigilance Associate (ST). All ADRs reported between 2013 and 2019 were included in the study.

The data was recorded and computerised in Microsoft Excel, using the protocols and formats provided by the National Coordination Centre of PvPI at the AMC. The data were analysed anonymously, at the group level. The analyses related to the patients' demography, frequency, and route of administration of ADR causative drugs, organ system involved and identification of the unlabelled ADRs were done using the established tools [11]. Appropriate descriptive statistics were used to analyse the quantitative data.

The causality of the reported adverse reactions was categorized into certain, probable, possible, un-assessable/unclassifiable, unlikely, conditional/unclassified using the WHO causality assessment scale [12]. This scale is used for the assessment of the relationship between a drug and the occurrence of an ADR. A serious reaction was characterized as a fatal, life threatening reaction that can prolong hospitalization, and cause a significant persistent disability, might result in a congenital anomaly, and require an intervention to prevent permanent damage or death. ADRs are categorised by Hartwig SC et. al. in seven levels based on their severity. Levels 1 and 2 falls under the mild category whereas levels 3 and 4 are under the moderate category and levels 5, 6 and 7 fall under the severe category [13]. The causal relationship between the suspected drug and the reaction was established using WHO–UMC standardized case causality assessment criteria [12].

Statistical methods

For peer review only - http://bmjopen.bmj.com/site/about/guidelines.xhtml

The data were analysed with Excel, and STATA V.13.1 (Stata Corp, College Station, Texas, USA). The frequencies and percentage of categorical values were calculated. Sum, median, mean, range and SD were calculated for the continuous numerical values. percentages were rounded to the closest whole number. The independent t-test was used for the comparison of normally distributed and continuous variables. The Chi^2 test was used for comparison of categorical values and p values <0.001 were considered as significant.

Patient and Public Involvement statement

Patients or the public (the patient or family members or a healthcare worker who noticed the ADR) were involved to report the ADRs in our research. The ADRs were recorded with the consent of the patient under Patient and Public Involvement. The person reporting an ADR could directly contact the pharmacovigilance team member and give the details of the ADR. The protocol of the collaborative project (WHO Collaborating Centre for International Drug Monitoring at Uppsala Monitoring Centre, Sweden) was followed.

Ethics statement

The study was approved by the Institutional Ethics Committee (IEC) of Ruxmaniben Deepchand Gardi Medical College, Ujjain, with approval number IEC Ref. No -68/2019. The personal identification variables were masked before analyses and analyses were conducted in groups to maintain full confidentiality. Since the results do not contain any personal information, taking consent from the patients was exempted. This was a non-interventional study where no influence was imposed on the treatment or medical care. Treating consultants were not approached regarding the reported ADRs as this was not the aim of the study. Full confidentiality was maintained throughout the analyses and the analyses were performed in groups to affirm patients' confidentiality. Necessary communication was accomplished with PvPI for permission to use the data for publication.

For peer review only - http://bmjopen.bmj.com/site/about/guidelines.xhtml

Results

Spontaneous ADR reports were collected and analysed during seven years at the AMC. A total of 1980 ADR reports were generated from 960 patients and were associated with 1316 prescriptions of drugs. The occurrence of ADRs dominated among males 64% (618) than female patients (Table 1). The maximum number of ADRs were reported were from the breadwinner group patients ageing between 19 and 65 years (81%), and the mean age of the patients was 38.02848 ± 0.5691212. A peak of ADR reports was attained in 2016 with 224 reports which decreased to 127 in 2019. The highest number of ADRs were reported in the year 2016 (224) while the maximum number of serious ADRs were reported in the year 2017 (105, Figure 1) and in the year 2018, the reports of serious ADRs exceeded the non-serious ADRs.

Table.1 Demographic parameter and route of drug administration among the patients developing ADRs at an AMC in Central India

Parameters	Number of Patients with ADRs, n (%)	P-value
**Total ADRs**	**960**	
**Age group**		
**0-18** years	89 (9)	
**19-65** years	773 (81)	0.0001
**>65** years	98 (10)	
**Sex**		
**Male**	618 (64)	0.0001
**Female**	342 (36)	
**Route of drug administration**		
**Oral**	987 (75)	0.0001
**Parenteral**	308 (23)	
**Topical**	21 (2)	

ADR=Adverse Drug Reaction, AMC=ADR Monitoring Centre, n=number

Figure 1 Frequency of type of ADRs reported per year at an AMC in Central India

Figure 2 summarises the number of ADRs associated with pharmacological drugs classes. In total, 1316 drugs were implicated in causing the ADRs and antimicrobials were associated with

For peer review only - http://bmjopen.bmj.com/site/about/guidelines.xhtml

the maximum number of the ADRs (29%) followed by the drugs of Antiretroviral therapy (ART, 19%) and Antitubercular therapy (ATT, 16%). The most common substances implicated for 88 ADRs were zidovudine followed by efavirenz (34 ADRs), ethambutol (44 ADRs) and ciprofloxacin (34 ADRs, Figure 3).

Figure 2 Occurrence of ADRs related to the pharmacological drug classes at an AMC in Central India

Figure 3 Pharmacological substances implicated in causing ADRs at an AMC in Central India

Table 2 presents the types of ADRs reported in various system organ classes (SOC). The ADRs of skin and subcutaneous tissue disorders (28%) were most common among all SOCs followed by gastrointestinal systems (18%).

Table 2 Frequency of ADR symptoms based on the involvement of organ system at an AMC in Central India

SOC (System Organ Class)	ADR symptoms n (%) 1980 (100)	Most common ADRs (n)
Skin and Subcutaneous Tissue Disorder	548 (28)	Rash (231), Generalized Pruritis (113), Fixed drug eruption (64), Burning sensation (19)
Gastrointestinal Disorders	365 (18)	Vomiting (91), Nausea (87), Abdominal discomfort (71), Diarrhoea (52)
Nervous System Disorder	219 (11)	Dizziness (61), Headache (50), Paraesthesia (17), Peripheral neuropathy (12)
General Disorders	193 (10)	Multi-drug resistance (35), Shivering (33), Generalized oedema (21), Weakness generalized (18)
Psychiatric Disorder	129 (7)	Anxiety (33), Sedation (24), Drowsiness (17), Psychosis (9)
Musculoskeletal and Connective Tissue Disorders	82 (4)	Myalgia (29), Tremors (17), Joint pain (7)
Respiratory Disorder	64 (3%)	Cough (18), Breathing difficulty (16), Dyspnoea (14)
Blood and Lymphatic System Disorder	60 (3%)	Anaemia (47), Thrombocytopenia (6)
Cardiac Disorder	45 (2%)	Tachycardia (19), Chest pain (16), Bradycardia (5)
Hepatobiliary Disorder	41 (2%)	Hepatotoxicity (39)
Nutritional and Metabolic Disorder	40 (2%)	Loss of appetite (28), Pallor (3), Weight gain (3)
Ear Disorder	31 (2%)	Impaired hearing (14), Tinnitus (8), Vertigo (8)
Vascular Disorder	31(2%)	Hypotension (16), Epistaxis (4), Hypertension (3)

For peer review only - http://bmjopen.bmj.com/site/about/guidelines.xhtml

Eye Disorder	26(1%)	Impaired vision (16), Eye irritation (2), Lacrimation (2)
Injury, Poisoning and Procedural Complications	25(1%)	Injection site reaction (12), Extravasation (6)
Renal Disorder	25(1%)	Increased serum creatinine (6), Haematuria (4), Dysuria (3)
Endocrine Disorders	23(1%)	Gynaecomastia (8), Moon face (5), Hypoglycaemia (3)
Immune System Disorder	19 (1%)	Allergic dermatitis (8), Allergic reactions (4), Anaphylactic reaction (4)
Electrolyte Imbalance	7(0.35%)	Hypokalemia (2), Hypocalcaemia (1), Hyperuricaemia (2)
Reproductive and Breast Disorder	7(0.35%)	Vaginal haemorrhage (2), Breast tenderness (1)

ADR=Adverse Drug Reaction, AMC=ADR Monitoring Centre, n=number

Table 3 shows the list of drugs that were most frequently associated with the ADR symptoms. Leflunomide followed by gliclazide, and paracetamol caused rashes. A fixed-dose combination of antibiotics, cefoperazone with sulbactam was most commonly associated with generalized pruritis. Zidovudine caused anaemia and the combination of tenofovir, lamivudine and efavirenz caused gynaecomastia. Antibiotics were tagged as suspected drugs for the ADRs in 204 reports. The most common indications for prescribing antibiotics among the 204 cases were multi-drug resistant tuberculosis (34 ADRs), bacterial infections (20 ADRs), and surgical prophylaxis (18 ADRs). Most patients were resistant to the fixed-dose combination of antibiotics i.e., ceftriaxone and sulbactam. In a majority of the cases (99%), antibiotics were prescribed either as post-surgical prophylaxis to the patients specified with a surgical procedure or to the trauma patients or patients with unclean and infected wounds as prophylaxis for suspicion of bacterial infection.

Table 3 Most frequently associated drugs with ADR symptoms at an AMC in Central India

ADR Symptom (n)	Name of the drug, suspected for the ADR symptom (n)
Rash (231)	Leflunomide (40), Gliclazide (21), Paracetamol (20)
Generalized pruritis (113)	Cefoperazone with sulbactam (12), Cefotaxime (8), Levofloxacin (8), Diclofenac (7)
Vomiting (91)	Tenofovir (22), Quinine (19)
Nausea (87)	Zidovudine (23), TLE (18), Quinine (11)
Abdominal pain (71)	Zidovudine (9), Aspirin (8), Leflunomide (4)
Dizziness (61)	TLE (21), Ranitidine (7), Quinine (6)
Anaemia (47)	Zidovudine (43)
Hepatotoxicity (39)	ATT (21)

For peer review only - http://bmjopen.bmj.com/site/about/guidelines.xhtml

Multi-drug resistance (35)	Ceftriaxone with tazobactam (15), Amikacin (9)
Anxiety (33)	TLE (12), Quinine (7)
Myalgia (29)	Zidovudine (173)
Injection site reaction (12)	Artesunate (3), Cefotaxime (3), Ceftriaxone (2)
Gynaecomastia (8)	TLE (7)
Increased serum creatinine (6)	Tenofovir (5)

n=number of reports, TLE=A combination of tenofovir, lamivudine and efavirenz, ATT= Antitubercular treatment

Table 4 shows the severity and causality assessment of reported ADRs. Four percent of all reported ADRs were severe and the rest were mild to moderate. Overall, 26 drugs were associated with 36 serious ADRs. Electrolyte imbalance, and reproductive and breast disorders caused seven ADR cases, while immune system disorder caused 19 ADR cases. The outcome was fatal in 12 ADR episodes, while 13 recovered with sequelae, 774 cases (55%) either recovered (521) or were recovering (253) at the time of ADR reporting and 90 were not having any sign of recovery till the ADR was reported.

Table 4 Assessment of severity and causality of the ADRs reported at an AMC in Central India

Type of Severity (HARTWIG SCALE)[8]	ADR n (%)
**Total**	960
Mild	564 (59) *
Moderate	360 (37.5)
Severe	36 (4)
**Causality Assessment (WHO Scale)[9]**	
Certain	93 (5)
Probable/Likely	846 (43)
Possible	951 (48)
Unlikely	22 (1)
**Inaccessible** / Unclassifiable	68 (3)

*Statistically significant p value>0.0001; ADR=Adverse Drug Reaction, n=number of ADRs

Discussion

There is a wide gap between the occurrence and reporting of ADRs, not only in India but also worldwide. The present study illustrates the ADRs reported at an AMC in Central India for the first time and present a list of the causative drugs for the burden of ADRs. The study highlighted

For peer review only - http://bmjopen.bmj.com/site/about/guidelines.xhtml

antimicrobials as the most common causative drugs for a high burden of ADRs followed by the ART drugs and the ATT drugs at the AMC. The occurrence of ADRs was more common in male patients (64%) and among patients of the breadwinner age group (19-65 years, 81%). Zidovudine was the agent that caused most ADRs (88) followed by ethambutol, ciprofloxacin and efavirenz.

Reporting of spontaneous ADRs is a collaborative study conducted with the help of inputs from healthcare providers or a person close to the patients. The maximum number of ADRs were reported in the year 2016 (224). The number of reported ADRs decreased after achieving a peak in the year 2016. The maximum number of serious ADRs were reported in 2017 and the non-serious ADRs in 2016. Although the data collection started in July 2013, yet the number of reported ADRs is lower for half a year. These low numbers might be due to the initial stage of the study when the collaborators were being sensitized for data collection. However, the reduction in the number of reported ADRs from 2016 onwards must act as a stimulus to plan future studies to identify the factors responsible for under-reporting including qualitative method of data collection. Extensive efforts are needed for the corrective measures to improve the reporting. In our study, significantly more ADRs were documented in the male patients than the female patients which are consistent with the earlier studies [14,15] whereas, few studies have also reported female preponderance [16,17]. However, the gender impact on the ADR occurrence cannot be explained here and might be an incidental finding and has no effect on the occurrence of reporting of the ADRs. In our study, the ADRs were predominantly reported in patients of age group between 19-65 years which is also similar to a study conducted by Daulat et al [14] and Thakare et al [17]. This is because it is a wide age group range, and this is likely the major population that attends hospital more frequently and receives drug therapy.

The results based on pharmacovigilance data are crucial to generate signals and alerts. However, signals are generated based on a substantial number of reports after following the

For peer review only - http://bmjopen.bmj.com/site/about/guidelines.xhtml

protocol. Yet, the frequencies of ADRs reported by some of the drugs in the present study are alarming. The pharmacological drug classes implicated for causing ADRs shows that antimicrobials including antibiotics caused the maximum number of ADRs (29%). This might be because antimicrobials are one of the most prescribed drug classes specifically in LMICs [18,19]. It is also documented that this class is prescribed for unindicated conditions providing an opportunity to develop antimicrobial resistance [15,20-22]. The ADR reports associated with antibiotics can facilitate the development of appropriate policies and guidelines for the judicious use of antibiotics and thereby combat antimicrobial resistance [23]. A fixed-dose combination of cefoperazone with sulbactam was most commonly associated with generalized pruritis. Among 35 cases of multi-drug resistance, most cases were encountered with a fixed-dose combination of ceftriaxone and sulbactam followed by Amikacin. Judicious selection of antibiotics based on the type of infection, local resistance pattern and prescribing guidelines is recommended [24]. However, in our study, the antibiotics were prescribed empirically as prophylaxis in a majority of the cases for suspicion of bacterial infection.

Initiating the treatment by prescribing antibiotics empirically with an intent to send samples for antibiotic susceptibility test (AST) and to modify the treatment based on the laboratory results is a standard procedure. However, the poor practice of sending samples for AST is a global issue and has also been reported earlier from the study settings[24,25]. Moreover, prescribing broad-spectrum antibiotics empirically and for unindicated conditions is one of the major causes of developing antibiotic resistance and has also been reported from the setting[24,26,27]. It is noteworthy that in the present study, the AST samples were sent mostly when the patient do not respond to the prescribed antibiotics. A few ADR reports specify that antibiotic prescriptions were modified based on the AST reports. The most common shifts were made from the combination of ceftriaxone with tazobactam to meropenem or linezolid. However, the availability of too few records limits us to comment any further. Therefore, we

For peer review only - http://bmjopen.bmj.com/site/about/guidelines.xhtml

suggest that every attempt should be made to encourage maximum use of laboratories to narrow down the antibiotic spectrum and minimize the risk of antibiotic resistance.

Besides antibiotics, the other commonly prescribed pharmacological drug classes implicated for ADRs were ART, ATT, non-steroidal anti-inflammatory drugs (NSAIDs), CNS and CVS drugs in descending order of causing ADRs. The ART and ATT drugs, despite their efficacy, are continuously found associated with a wide range of potential adverse effects [28]. The occurrence of ADRs related to ART and ATT drugs may result in poor compliance with the treatment that can lead to the development of resistance and treatment failure including virologic failures [29-31]. No common drug class could be implicated for causing serious ADRs events.

In the present study, the most common drugs causing rash were leflunomide followed by gliclazide and paracetamol. While zidovudine was most notorious for already labelled as associated ADRs such as anaemia, myalgia, nausea and abdominal pain. Anaemia is found to be associated with both the dose and stage of disease and myopathy is the most significant yet, manageable type of late adverse reaction [32].

The combination of tenofovir, lamivudine and efavirenz was most commonly associated with vomiting, dizziness, anxiety and gynaecomastia. Among these ADRs association with dizziness draws attention. It has been found that those patients who did not report such ADRs to the attending physicians end up having severe diseases. The debilitating nature of the symptoms and awareness levels of the patients about, what should report to the attending doctor, plays an important role in reporting ADRs. Therefore, increasing awareness of patients is of utmost importance via asking direct questions about the ADRs caused by the flagged drugs [33-34]. In addition, a plan could be developed to disseminate the findings of the pharmacovigilance surveillance to the prescribers so that they can avoid prescribing the drugs

For peer review only - http://bmjopen.bmj.com/site/about/guidelines.xhtml

responsible for ADRs, wherever possible. In cases where it is not possible to avoid the drug, for example in ART, the prescribers can counsel patients based on the associations found via pharmacovigilance. This will help to improve patient's compliance and will motivate patients to seek early advice in case of occurrence of ADRs.

Polypharmacy is prevalent globally and the occurrence of ADR is directly proportional to the number of prescribed drugs thus, polypharmacy must be discouraged [35,36]. However, in the present study single drug was attributed for most of the ADRs (73%). It was observed that in most of the reports the drug that was most suspected of being associated with the specific ADR was reported and not the whole treatment. NSAIDs are often a constituent of many rational and irrational fixed-dose combinations and this could be a cause of the high incidence of the ADRs related to the NSAIDs [37]. This was reflected in our study where besides antimicrobials and ART drugs the antiepileptics and NSAIDs also caused substantial ADRs [19-22, 38-42]. In the present study, the most prescribed pharmacological substances among the patients having ADRs were zidovudine, ethambutol and ciprofloxacin while in other studies ceftriaxone, zidovudine, and phenytoin sodium were the most prescribed drugs [38-42]. Zidovudine and ethambutol are part of multidrug ATT and ART regimes, respectively. Factors including the dose of the drugs, drug-drug interactions as well as genetic differences of the study population could also affect the number of adverse drug reactions caused by drugs [43].

Most of the patients had mild ADRs in our study, as observed in other Indian studies [15,17]. ADRs of skin and subcutaneous tissue disorders (28%) were most common among all SOCs. Skin rashes were the most common complaints followed by gastrointestinal complaints (18%), nervous system disorders (11%), general disorders (10%) and psychiatric disorders (7%). Skin and subcutaneous tissues are among the most targeted organs for ADRs. The disorders are reported more frequently because these develop within one week of drug administration and can easily be spotted when the patients are still under the close supervision of the healthcare

For peer review only - http://bmjopen.bmj.com/site/about/guidelines.xhtml

providers [15,44,45]. Timely recognition of skin ADRs is crucial for its classification, management, and causality assessment.

The causality assessment was conducted based on the WHO probability scale. According to the assessment, most cases could be categorized as 'Possible' (48) followed by 'Probable' (43). ADRs are rarely categorised as 'Certain' as it requires to re-challenge the patient with the same causative drug, which is not ethical, hence the assessment frequently goes to the Possible and Probable category [15,46]. Though 26 drugs were associated with serious, life-threatening ADRs, yet none of the drug classes could be implicated for serious ADRs. This was because of insufficient data due to the under-reporting of ADRs, and the wide range of drugs prescribed. An adequate ADR reporting is required to preserve the clinical information that could prevent significant damage to patients, plan and implement measures to reduce their impact on public health and consequently minimize the burden on the health care system. Under-reporting a challenge in pharmacovigilance when other AMC contributed with 212 ADRs per month our AMC reported an average of 19 ADRs per month in 2016 while dropped to 11 in 2019 [47].

Early identification and management of ADRs will not only improve patient safety, quality of life but also reduce the cost of treatment. Labelling of new ADRs will help to prepare the prescribing policies and modify the prescribing patterns. Reduction in prescribing of the flagged drugs will reduce the prevalence of ADRs. We believe that disseminating results to the prescribers through feedback might reduce prescribing of causative drugs and improve reporting of ADRs. A knowledge, attitude and practice-based study conducted in Norway found that ADRs can be prevented by collecting reliable information about their frequencies and possible risk factors [7]. Therefore, we propose to develop and implement strategies for the community but specifically for the HCWs to improve reporting and monitoring of ADRs. We also propose to disseminate the study results in the form of a feedback method to improve reporting. This might also help in the reduction of prescription of the drugs causing most of the

For peer review only - http://bmjopen.bmj.com/site/about/guidelines.xhtml

ADRs. The methods of the feedback study could be shared and tested at other similar settings as well.

Conclusion

The maximum number of ADRs were reported from the breadwinner group (males and 19-65 years). Antimicrobials caused the maximum number of ADRs. This highlights an alarming situation in addition to antimicrobial resistance. Prudent use of antimicrobials is once again proven as need of the hour. ADRs of skin and subcutaneous tissue disorders were most frequent among all SOCs. Most of the ADRs were mild and belonged to the 'Possible' group of probability scale. However, under-reporting of ADRs at our AMC is evident and a major factor for the pause in performing severity and causality assessment. Thus, the prevalence of ADRs reported in the present study can be perceived as the tip of an iceberg.

To deal with the problem of under-reporting we suggest continuous training programmes for HCPs, seeking the collaboration of nearby health care facilities, and most importantly giving feedback to the HCWs to impart confidence, awareness and knowledge about the ADR causing drugs and will encourage reporting ADR events.

Future implications

Our results indicate the need to develop and test a model of motivational educational- approach focusing on health care workers and the community to increase ADR reporting in the settings associated with our AMC. Neither the global pharmacovigilance nor the PvPI has designed a plan, to analyse and provide feedback on the ADR data to the prescribers. Providing reliable and balanced information, and a valid assessment of the risk-benefit profile of the marketed drugs to the healthcare professionals at a local level is essential. This knowledge might improve patients' safety while prescribing drugs, support patient care and public health programs in the

For peer review only - http://bmjopen.bmj.com/site/about/guidelines.xhtml

settings. Therefore, we propose to develop a model with a strategy to provide feedback and knowledge to improve ADR reporting. Furthermore, providing feedback to the prescribers and sensitizing them about the flagged drugs will be an encouragement for reporting ADRs. HCPs will be assured that reporting ADRs has no legal implications.

Modification of the organizational structure, training of HCPs and availability of resources can improve early detection of ADRs. We also suggest planning and start an ADR awareness and training campaign in collaboration with HCPs of the hospitals in the periphery, district, and private hospitals to motivate them to report ADRs. However, the long-term sustainability of these measures is most crucial as the effectiveness of interventions diminishes with time. The use of a digital platform such as SMS and E-mails might play an important role in reminding and keeping them motivated and sensitized to report ADRs timely.

Acknowledgement

The authors acknowledge the active support of the consultants and physicians in data collection. The Authors extend thanks to the IPC PvPI, Ghaziabad for encouraging and permitting the publication. Thanks are due to the management of the AMC- RDGMC for their support. The authors also acknowledge Mr Amit Pawar for his assistance during the study.

Author Contributions

MS and RB conceptualized the study and designed the analyses. ST, MS, RB and SA collected the data. MS and RB performed the analyses and drafted the manuscript. All authors contributed to and approved the present form of the manuscript.

Funding statement

This research received no specific grant from any funding agency in the public, commercial, or not-for-profit sectors

For peer review only - http://bmjopen.bmj.com/site/about/guidelines.xhtml

Competing interest

The authors declare no competing interests.

Data Sharing statement

As per the protocol of the institutional ethics committee to maintain full confidentiality, the data can be made available through a request form addressing, The Chairman, Ethics Committee, R.D. Gardi Medical College, Agar Road, Ujjain, Madhya Pradesh, India 456006 (email: iecrdgmc@yahoo.in, uctharc@sancharnet.in), giving all details of the article. Quote the IEC approval number: 68/2019 along with the request.

Licence statement

The Corresponding Author has the right to grant on behalf of all authors and does grant on behalf of all authors, an exclusive licence on a worldwide basis to the BMJ Publishing Group Ltd ("BMJ"), and its Licensees to permit this article (if accepted) to be published in The BMJ's editions and any other BMJ products and to exploit all subsidiary rights.

The TRIPOD checklist indicating the page and line numbers of our manuscript is included with the cover letter.

For peer review only - http://bmjopen.bmj.com/site/about/guidelines.xhtml

References

1. Vivekanandan K, Thota P, Janarthanan VV, Singh GN. Pharmacovigilante's in the pharmacovigilance programme of India: Ideal qualities and skills. J Young Pharm 2016;8:291-2. (Assessed 08 April 2021)
2. Nirojini PS, Yemineni R, Nadenla RR. Monitoring and Reporting of Adverse Drug Reactions in A South Indian Tertiary Care Hospital. Int J Pharm Sci Rev Res. 2014;24(1):259-62.
3. Datta S, Sengupta S. An evaluation of knowledge, attitude, and practice of adverse drug reaction reporting in a tertiary care teaching hospital of Sikkim. Perspect Clin Res 2015;6:200-6.
4. Mittal N, Mittal R, Gupta MC. An overview of the pharmacovigilance system in India. Clin Res Regul Aff 2016;33:4-8.
5. Tandon VR, Mahajan V, Khajuria V, Gillani Z. Under-reporting of adverse drug reactions: a challenge for pharmacovigilance in India. Indian J Pharmacol. 2015;47(1):65-71. doi:10.4103/0253-7613.150344 (Assessed 08 April 2021)
6. Hazell L, Shakir SAW. Under-reporting of adverse drug reactions: A systematic review. Drug Saf. 2006; 29 (5) :385-96.366

For peer review only - http://bmjopen.bmj.com/site/about/guidelines.xhtml

7. Castberg I, Reimers A, Sandvik P, Aamo TO, Spiqset O. Adverse drug reactions of antidepressants and antipsychotics: Experience, knowledge and attitudes among Norwegian psychiatrists. Nord J Psychiatry. 2006;60:227–33.
8. Indian Pharmacopoeia Commission, Ministry of Health and family Welfare, Govt. of India. www.ipc.gov.in (Assessed 08 April 2021)
9. ADR form PvPI. Available from: http://www.cdsco.nic.in/writereaddata/ADR%20form%20PvPI.pdf. (Assessed 08 April 2021)
10. IA Eland, KJ Belton, AC van Grootheest, AP Meiners, MD Rawlins, and BH Ch Stricker. Attitudinal survey of voluntary reporting of adverse drug reactions British journal of clinical Pharmacology1999 Oct; 48(4): 623–627.doi: 10.1046/j.1365-2125.1999.00060.x (Assessed 08 April 2021)
11. Schumock GT, Seeger JD, Kong SX. Control charts to monitor rates of adverse drug reactions. Hosp Pharm 1995;30:1088, 1091-2,1095-6.
12. The use of the WHO–UMC system for standardised case causality assessment. Accessed from: http://www.WHO-UMC.org/graphics/4409.pdf (Assessed 08 April 2021)
13. Hartwig SC, Siegel J, Schneider PJ. Preventability and severity assessment in reporting adverse drug reactions. Am J Hosp Pharm 1992;49:2229-32.
14. Daulat MP, Abishake VJ, Singh P, Raj B. A prospective study of adverse drug reactions in a tertiary care teaching hospital. Int J Basic Clin Pharmacol 2018;7:1965-69.
15. Ramakrishnaiah H et al. Int J Basic Clin Pharmacol. 2015 Jun;4(3):515-521 International Journal of Basic & Clinical Pharmacology.
16. Singh P, Agrawal M, Hishikar R, Joshi U, Maheshwari B, Halwai A. Adverse drug reactions at adverse drug reaction monitoring center in Raipur: Analysis of spontaneous reports during 1 year. Indian J Pharmacol. 2017;49(6):432-437. doi:10.4103/ijp.IJP_781_16 (Assessed 08 April 2021)

For peer review only - http://bmjopen.bmj.com/site/about/guidelines.xhtml

17. Thakare VS, Kavitha V D, Langade D. Prospective observational study to evaluate adverse drug reactions pattern in a tertiary level teaching hospital. Natl J Physiol Pharm Pharmacol 2019;9(5):434-437.

18. Landstedt K, Stålsby-Lundborg C, Sharma A, Johansson F, Sharma M. Antibiotic prescriptions to the inpatients having non-bacterial diagnosis at medicine departments of two private sector hospitals in Madhya Pradesh, India: a cross sectional study. *BMJ Open* 2017;7:e012974. doi:10.1136/bmjopen-2016- 012974. Available at URL- http://bmjopen.bmj.com/content/bmjopen/7/4/e012974.full.pdf (Assessed 08 April 2021)

19. Anna Makhowska, Jonatan Sparentofft, Cecilia Stålsby Lundborg, Megha Sharma. Perioperative antibiotic prescribing in surgery departments of two private sector hospitals in Madhya Pradesh, India. Perioperative Medicine. (2019) 8:10. DOI: https://doi.org/10.1186/s13741-019-0121-3 and https://doi.org/10.1186/s13741-019-0123-1 (Assessed 08 April 2021)

20. Wester K, Jönsson AK, Spigset O, Druid H, Hägg S. Incidence of fatal adverse drug reactions: a population based study. Br J Clin Pharmacol. 2008;65:573-9.

21. Gor AP, Desai SV. Adverse drug reactions (adr) in the inpatients of medicine department of a rural tertiary care teaching hospital and influence of pharmacovigilance in reporting ADR. Indian J Pharmacol. 2008;40(1):37-40.

22. Vora MB, Trivedi HR, Shah BK, Tripathi CB. Adverse drug reactions in inpatients of internal medicine wards at a tertiary care hospital: a prospective cohort study. J Pharmacol Pharmacother. 2011;2(1):21-5.

23. Habarugira, JMV, Figueras, A. Pharmacovigilance network as an additional tool for the surveillance of antimicrobial resistance. Pharmacoepidemiol Drug Saf. 2021; 30: 1123–1131. https://doi.org/10.1002/pds.5249

For peer review only - http://bmjopen.bmj.com/site/about/guidelines.xhtml

24. World Health Organization. Global guidelines for the prevention of surgical site infection 2016. Available from: http://apps.who.int/iris/bitstream/10665/250680/1/9789241549882-eng.pdf?ua=1. (Assessed 09 September 2021)

25. Sharma M, Damlin AL, Sharma A, Stålsby Lundborg C. Antibiotic prescribing in medical intensive care units--a comparison between two private sector hospitals in Central India. Infect Dis (Lond). 2015 May;47(5):302-9. doi: 10.3109/00365548.2014.988747. Epub 2015 Feb 24. PMID: 25708090. (Assessed 09 September 2021)

26. Landstedt K, Sharma A, Johansson F, et al. Antibiotic prescriptions for inpatients having non-bacterial diagnosis at medicine departments of two private sector hospitals in Madhya Pradesh, India: a cross-sectional study. BMJ Open 2017;7:e012974. doi:10.1136/bmjopen-2016-012974 (Assessed 09 September 2021)

27. National Treatment Guidelines for Antimicrobial Use in Infectious Diseases, NATIONAL CENTRE FOR DISEASE CONTROL Directorate General of Health Services Ministry of Health & Family Welfare Government of India. https://ncdc.gov.in/WriteReadData/l892s/File622.pdf (Assessed 09 September 2021)

28. Shet A, Antony J, Arumugam K, Kumar Dodderi S, Rodrigues R, DeCosta A (2014) Influence of Adverse Drug Reactions on Treatment Success: Prospective Cohort Analysis of HIV-Infected Individuals Initiating First-Line Antiretroviral Therapy in India. PLoS ONE 9(3): e91028. https://doi.org/10.1371/journal.pone.0091028 (Assessed 08 April 2021)

29. Manju R, Maheshwari YN, Ramya JE, Krishnamoorthy K. Adverse drug reaction monitoring of antitubercular drugs during intensive phase at tertiary care medical college hospital: A prospective study. Natl J Physiol Pharm Pharmacol 2020;10(11):976-980.

For peer review only - http://bmjopen.bmj.com/site/about/guidelines.xhtml

30. Mukherjee S, Era N, Saha B, Tripathi SK. Adverse drug reaction monitoring in patients on antiretroviral therapy in a tertiary care hospital in Eastern India. *Indian J Pharmacol*. 2017;49(3):223-228. doi:10.4103/ijp.IJP_304_16 (Assessed 08 April 2021)

31. Abah IO, Ncube NBQ, Bradley HA, AgbaJi OO, Kanki P. Antiretroviral Therapy-associated Adverse Drug Reactions and their Effects on Virologic Failure- A Retrospective Cohort Study in Nigeria. Curr HIV Res. 2018;16(6):436-446. doi:10.2174/1389450120666190214144609 (Assessed 08 April 2021)

32. Rachlis A, Fanning MM. Zidovudine toxicity. Clinical features and management. Drug Saf. 1993 Apr;8(4):312-20. doi: 10.2165/00002018-199308040-00005. PMID: 8481217. (Assessed 10 August 2021)

33. Khoza-Shangase K. VESTIBULAR FUNCTION IN A GROUP OF ADULTS WITH HIV/AIDS ON HAART. Afr J Infect Dis. 2017;12(1):7-14. Published 2017 Nov 15. doi:10.21010/ajid.v12i1.2 (assessed 10 August 2021)

34. Molla Y, Wubetu M, Dessie B. Anti-Tuberculosis Drug Induced Hepatotoxicity and Associated Factors among Tuberculosis Patients at Selected Hospitals, Ethiopia. Hepat Med. 2021;13:1-8 https://doi.org/10.2147/HMER.S290542 (Assessed 10 August 2021)

35. Nguyen JK, Fouts MM, Kotabe SE, Lo E. Polypharmacy as a risk factor for adverse drug reactions in geriatric nursing home residents. Am J Geriatr Pharmacother. 2006 Mar;4(1):36-41. doi: 10.1016/j.amjopharm.2006.03.002. PMID: 16730619. (Assessed 08 April 2021)

36. R., Siddarama, Bharath Naidu J., Joshisree K. P., and Sahithi Lakshmi V. POLYPHARMACY INDUCED DRUG INTERACTIONS, ADVERSE DRUG REACTIONS (ADR) AND MEDICATION ERRORS IN TERTIARY CARE SOUTH INDIAN HOSPITAL. International Journal of Pharmacy and Pharmaceutical Sciences,

For peer review only - http://bmjopen.bmj.com/site/about/guidelines.xhtml

Vol. 11, no. 2, Feb. 2019, pp. 88-93, doi:10.22159/ijpps.2019v11i2.29508. (Assessed 09 August 2021)

37. Gor AP, Saksena M. Adverse drug reactions of nonsteroidal anti-inflammatory drugs in orthopedic patients. J Pharmacol Pharmacother. 2011;2(1):26-29. doi:10.4103/0976-500X.77104 (Assessed 08 April 2021)

38. Kathiria JM, Sattigere BM, Desai PM, Patel SP. A study of adverse drug reactions in patients admitted to intensive care unit of a tertiary care teaching rural hospital. Int J Pharm Pharm Sci. 2013;5(1):160-3.

39. Sharma SK. Zidovudine-induced anaemia in HIV/AIDS. Indian J Med Res. 2010;132:359-61.

40. Shah SP, Desai MK, Dikshit RK. Analysis of cutaneous adverse drug reactions at a tertiary care hospital: a prospective study. Trop J Pharm Res. 2011;10(4):517-22.

41. Vijendra R, Pundarikaksha HP, Gopal MG, Girish K, Vasundara K, Jyothi R. A prospective study of cutaneous adverse drug reaction in a tertiary care hospital. Natl J Basic Med Sci. 2013;3(1):44-51.

42. Hussain MM, Girhepunje K, Pal R, Siddiqua SS. Incidence of adverse drug reactions in a tertiary care hospital: a systematic review and meta-analysis of prospective studies. Pharm Lettr. 2010;2(3):358-68.

43. Hema NG, Bhuvana KB, Virupaksha HM. Critical Assessment of Adverse drug Reactions to Antitubercular Drugs in a Government Teaching Hospital. IJBMS, 2013; 4(2):60-67.

44. Arulmani R, Rajendran SD, Suresh B. Adverse drug reaction monitoring in a secondary care hospital in South India. Br J Clin Pharmacol. 2008;65(2):210-6.

45. Modi A, Desai M, Shah S, Shah B. Analysis of Cutaneous Adverse Drug Reactions Reported at the Regional ADR Monitoring Center. Indian J Dermatol. 2019;64(3):250.

For peer review only - http://bmjopen.bmj.com/site/about/guidelines.xhtml

doi:10.4103/ijd.IJD_682_16 https://www.ncbi.nlm.nih.gov/pmc/articles/PMC6537693/ (Assessed 08 April 2021)

46. Laskar JI, Chakravarty P, Dewan B. A study on incidence of adverse drug reactions with commonly prescribed drugs and causality assessment in Silchar Medical College and Hospital. Int J Basic Clin Pharmacol 2017;6:1175-83.

47. Tandon VR, Mahajan V, Khajuria V, Gillani Z. Under-reporting of adverse drug reactions: a challenge for pharmacovigilance in India. Indian J Pharmacol. 2015;47(1):65-71. doi:10.4103/0253-7613.150344

For peer review only - http://bmjopen.bmj.com/site/about/guidelines.xhtml

Figure 1 Frequency of type of ADRs reported per year at an AMC in Central India

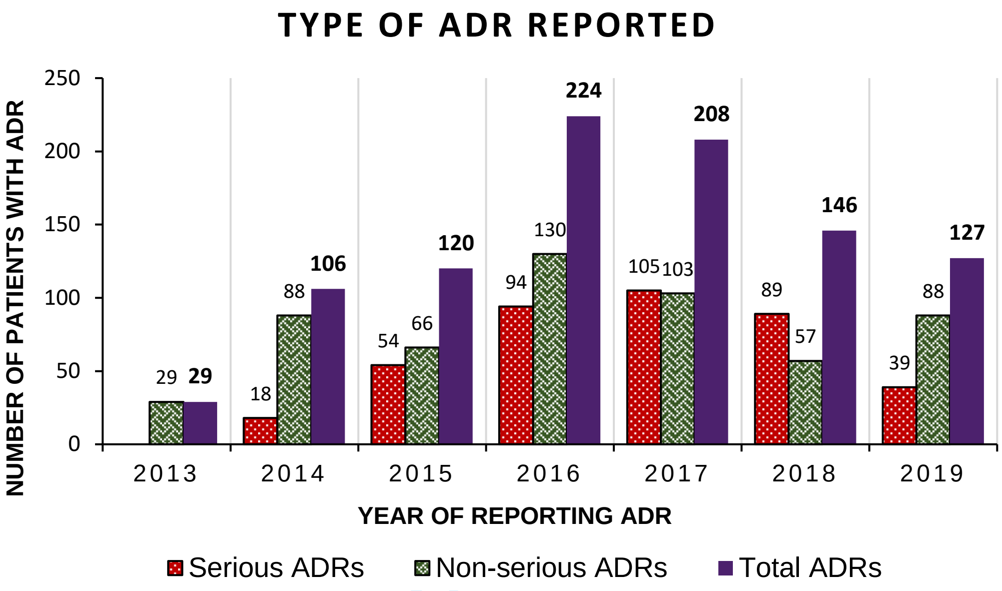

ADR=Adverse Drug Reaction, AMC=ADR Monitoring Centre

Figure 2 Occurrence of ADRs related to the pharmacological drug classes at an AMC in Central India

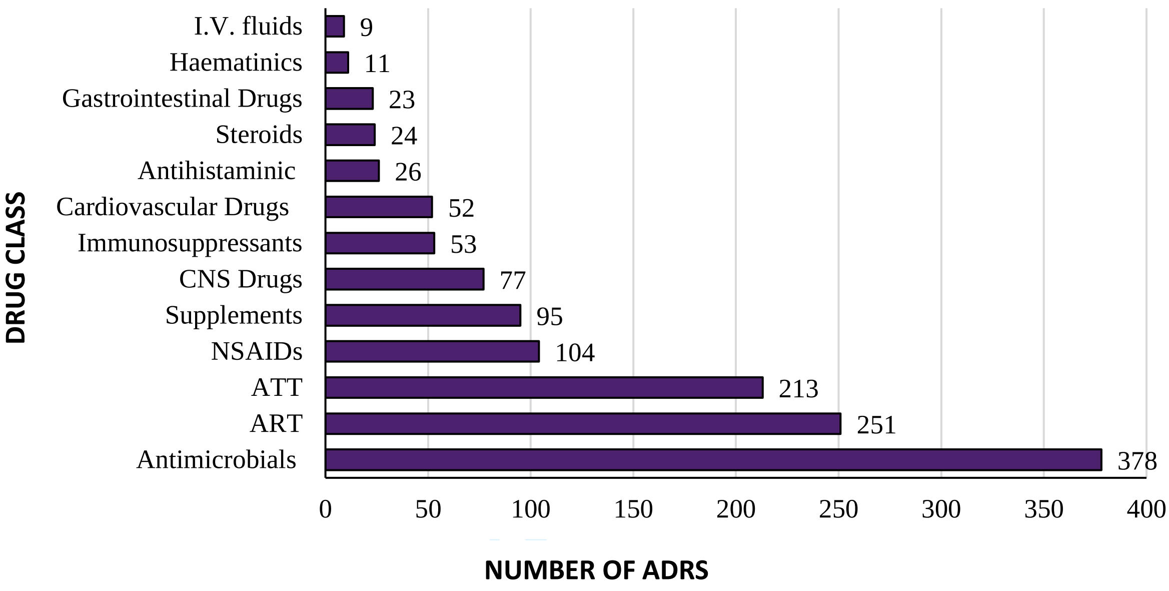

ADR=Adverse Drug Reaction, AMC=ADR Monitoring Centre ART=Antiretroviral therapy, ATT=Antitubercular therapy, CNS=Central Nervous System, NSAID= Non-steroidal anti-inflammatory drugs, I.V. fluids= Intravenous fluid

For peer review only

For peer review only

Figure 3: Pharmacological substances implicated in causing ADRs at an AMC in Central India

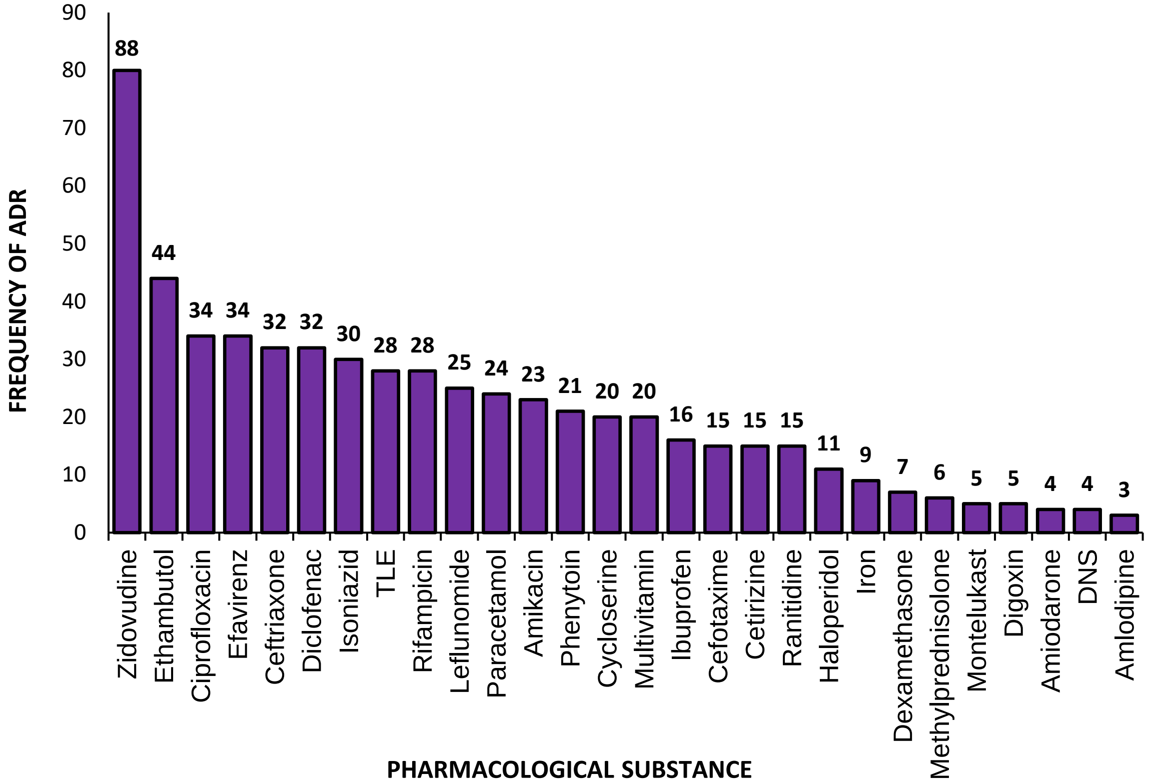

ADR=Adverse Drug Reaction, AMC=ADR Monitoring Centre, DNS=Dextrose Normal Saline, T/L/E=Tenofovir/Lamivudine/Efaviren

ANNEXURE – I

Version-1.3

SUSPECTED ADVERSE DRUG REACTION REPORTING FORM

For VOLUNTARY reporting of Adverse Drug Reaction by Healthcare Professionals

INDIAN PHARMACOPOEIA COMMISSION (National Coordination Centre-Pharmacovigilance Programme of India)
Ministry of Health & Family Welfare, Government of India Sector-23, Raj Nagar, Ghaziabad-201002

A. PATIENT INFORMATION

1. Patient Initials ________	2. Age at the time of Event or Date of Birth ________	3. M ☐ F ☐ Other ☐<br>4. Weight________Kgs

Reg. No. /IPD No. /OPD No. /CR No. :

AMC Report No. :

Worldwide Unique No. :

B. SUSPECTED ADVERSE REACTION

5. Event/Reaction start date (dd/mm/yyyy)

6. Event/Reaction stop date (dd/mm/yyyy)

6 (A). Onset Lag Time

7. Describe Event/Reaction with treatment details, if any

12. Relevant tests/ laboratory data with dates

13. Relevant medical/medication history (e.g. allergies, race, pregnancy, smoking, alcohol use, hepatic/renal dysfunction, past surgery etc.)

14. Seriousness of the reaction: No ☐ if Yes ☐(please tick anyone)

☐ Death (dd/mm/yyyy) ☐ Congenital-anomaly
☐ Life threatening ☐ Disability
☐Hospitalization/Prolonged ☐ Other Medically important

15. Outcomes

☐ Recovered ☐ Recovering ☐ Not recovered
☐ Fatal ☐ Recovered with sequelae ☐ Unknown

C. SUSPECTED MEDICATION(S)

S.No	8. Name (Brand/Generic)	Manufacturer (if known)	Batch No. / Lot No.	Exp. Date (if known)	Dose used	Route used	Frequency (OD, BD etc.)	Therapy dates		Indication	Causality Assessment
								Date started	Date stopped		
i											
ii											
iii											
iv*											

S.No as per C	9. Action Taken (please tick)						10. Reaction reappeared after reintroduction (please tick)			
	Drug withdrawn	Dose increased	Dose reduced	Dose not changed	Not applicable	Unknown	Yes	No	Effect unknown	Dose (if reintroduced)
i										
ii										
iii										
iv										

11. Concomitant medical product including self-medication and herbal remedies with therapy dates (Exclude those used to treat reaction)

S.No	Name (Brand/Generic)	Dose used	Route used	Frequency (OD, BD, etc.)	Therapy dates		Indication
					Date started	Date stopped	
i							
ii							
iii*							

Additional Information:

D. REPORTER DETAILS

16. Name and Professional Address:________________________
____
Pin:___________E-mail________________________________
Tel. No. (with STD code)______________________________
Occupation:__________________ Signature:_______________

17. Date of this report (dd/mm/yyyy):

Sig. and Name of Receiver-

Confidentiality: The patient's identity is held in strict confidence and protected to the fullest extent. Submission of a report does not constitute an admission that medical personnel or manufacturer or the product caused or contributed to the reaction. Submission of an ADR report does not have any legal implication on the reporter.

*use separate page for more information

TRIPOD Checklist: Prediction Model Development

Section/ Topic	Item	Checklist Item	Page
**Title and abstract**			
Title	1	Identify the study as developing and/or validating a multivariable prediction model, the target population, and the outcome to be predicted.	1
Abstract	2	Provide a summary of objectives, study design, setting, participants, sample size, predictors, outcome, statistical analysis, results, and conclusions.	2
**Introduction**			
Background and objectives	3a	Explain the medical context (including whether diagnostic or prognostic) and rationale for developing or validating the multivariable prediction model, including references to existing models.	3
	3b	Specify the objectives, including whether the study describes the development or validation of the model or both.	4
**Methods**			
Source of data	4a	Describe the study design or source of data (e.g., randomized trial, cohort, or registry data), separately for the development and validation data sets, if applicable.	4
	4b	Specify the key study dates, including start of accrual; end of accrual; and, if applicable, end of follow-up.	4
Participants	5a	Specify key elements of the study setting (e.g., primary care, secondary care, general population) including number and location of centres.	4
	5b	Describe eligibility criteria for participants.	4
	5c	Give details of treatments received, if relevant.	4,5
Outcome	6a	Clearly define the outcome that is predicted by the prediction model, including how and when assessed.	4-6
	6b	Report any actions to blind assessment of the outcome to be predicted.	NA
Predictors	7a	Clearly define all predictors used in developing or validating the multivariable prediction model, including how and when they were measured.	NA
	7b	Report any actions to blind assessment of predictors for the outcome and other predictors.	7
Sample size	8	Explain how the study size was arrived at.	NA
Missing data	9	Describe how missing data were handled (e.g., complete-case analysis, single imputation, multiple imputation) with details of any imputation method.	7
Statistical analysis methods	10a	Describe how predictors were handled in the analyses.	7
	10b	Specify type of model, all model-building procedures (including any predictor selection), and method for internal validation.	NA
	10d	Specify all measures used to assess model performance and, if relevant, to compare multiple models.	NA
Risk groups	11	Provide details on how risk groups were created, if done.	NA
**Results**			
Participants	13a	Describe the flow of participants through the study, including the number of participants with and without the outcome and, if applicable, a summary of the follow-up time. A diagram may be helpful.	6
	13b	Describe the characteristics of the participants (basic demographics, clinical features, available predictors), including the number of participants with missing data for predictors and outcome.	8-11
Model development	14a	Specify the number of participants and outcome events in each analysis.	8
	14b	If done, report the unadjusted association between each candidate predictor and outcome.	NA
Model	15a	Present the full prediction model to allow predictions for individuals (i.e., all regression coefficients, and model intercept or baseline survival at a given time point).	NA

TRIPOD Checklist: Prediction Model Development

specification	15b	Explain how to the use the prediction model.	17
Model performance	16	Report performance measures (with CIs) for the prediction model.	NA
**Discussion**			
Limitations	18	Discuss any limitations of the study (such as nonrepresentative sample, few events per predictor, missing data).	2,16
Interpretation	19b	Give an overall interpretation of the results, considering objectives, limitations, and results from similar studies, and other relevant evidence.	12-17
Implications	20	Discuss the potential clinical use of the model and implications for future research.	16-18
**Other information**			
Supplementary information	21	Provide information about the availability of supplementary resources, such as study protocol, Web calculator, and data sets.	NA
Funding	22	Give the source of funding and the role of the funders for the present study.	18

We recommend using the TRIPOD Checklist in conjunction with the TRIPOD Explanation and Elaboration document.

For peer review only
